# Peptidomimetic Star Polymers for Targeting Biological Ion Channels

**DOI:** 10.1371/journal.pone.0152169

**Published:** 2016-03-23

**Authors:** Rong Chen, Derong Lu, Zili Xie, Jing Feng, Zhongfan Jia, Junming Ho, Michelle L. Coote, Yingliang Wu, Michael J. Monteiro, Shin-Ho Chung

**Affiliations:** 1 Research School of Biology, Australian National University, Canberra ACT 2601, Australia; 2 Australian Institute for Bioengineering and Nanotechnology, The University of Queensland, Brisbane QLD 4072, Australia; 3 College of Life Sciences, Wuhan University, Wuhan 430072, China; 4 ARC Centre of Excellence for Electromaterials Science, Research School of Chemistry, Australian National University, Canberra ACT 2601, Australia; Indiana University School of Medicine, UNITED STATES

## Abstract

Four end-functionalized star polymers that could attenuate the flow of ionic currents across biological ion channels were first *de novo* designed computationally, then synthesized and tested experimentally on mammalian K^+^ channels. The 4-arm ethylene glycol conjugate star polymers with lysine or a tripeptide attached to the end of each arm were specifically designed to mimic the action of scorpion toxins on K^+^ channels. Molecular dynamics simulations showed that the lysine side chain of the polymers physically occludes the pore of Kv1.3, a target for immuno-suppression therapy. Two of the compounds tested were potent inhibitors of Kv1.3. The dissociation constants of these two compounds were computed to be 0.1 μM and 0.7 μM, respectively, within 3-fold to the values derived from subsequent experiments. These results demonstrate the power of computational methods in molecular design and the potential of star polymers as a new infinitely modifiable platform for ion channel drug discovery.

## Introduction

Peptide-based drugs have attracted growing attention as available small molecule drugs often suffer from poor specificity and significant side effects. Although peptide therapeutics have great potentials in the treatment of many diseases, the main drawback is their short lifetime *in vivo* due to rapid degradation by proteases, low stability in plasma and rapid clearance from circulation [1]. The introduction of synthetic scaffolds decorated with peptide or peptide fragments (i.e., peptidomimetics) overcomes many of these stability issues [2]. Poly(L-lysine) dendrimers, for example, with multivalent lysine groups on the peripheral layer of the dendrimer are in clinical trials as an antiviral topical ointment [3, 4]. Dendritic polymers decorated with epitopes have also been shown to be an effective self-adjuvanting vaccine [5, 6]. The next great challenge is to *a priori* design synthetic peptides to enhance polyvalent interactions to enable selective binding and targeting to ion channels.

Malfunction of ion channels is implicated in the development of a host of human diseases such as neurological, muscular and immunological disorders. Various ion channels have been identified as pharmaceutical targets [7, 8], and a range of currently available drugs such as local anesthetics and anticonvulsants modulate ion channel function [9]. Many natural polypeptides isolated from the venom of arachnids, reptiles and marine invertebrates modulate the function of ion channels, either by physically occluding the ion conduction pathway or by interfering with their gating mechanisms. As some of these venom peptides are highly specific inhibitors for certain channel isoforms, extensive effort has been made to develop novel drugs using venom peptides as scaffolds [10, 11]. However, these toxins are relatively expensive to manufacture, and thus the cost for drug development can be high [12]. Also, the immune system may generate antibodies to compromise the efficacy of the peptide toxins.

In the past two decades, there have been rapid advances in determination of the tertiary structures of ion channels [13–15] and venom peptides [16] by X-ray crystallography and solution NMR. These structures have enabled theoretical modelling of peptide-channel interactions in atomic detail [17]. With the development of new analytical methods and increasing computational power, the binding affinity of a given toxin to a specific channel can be computed to within one order of magnitude to the value determined experimentally (see Table A in S1 File) [17]. The mechanisms by which peptide toxins selectively inhibit several isoforms of voltage-gated K^+^ (Kv) channels have been elucidated from both theoretical and experimental perspectives [17, 18]. The understanding of toxin action on a molecular level would enable the rational design of toxin analogues as novel ion channel modulators and drug scaffolds.

**Fig 1:**
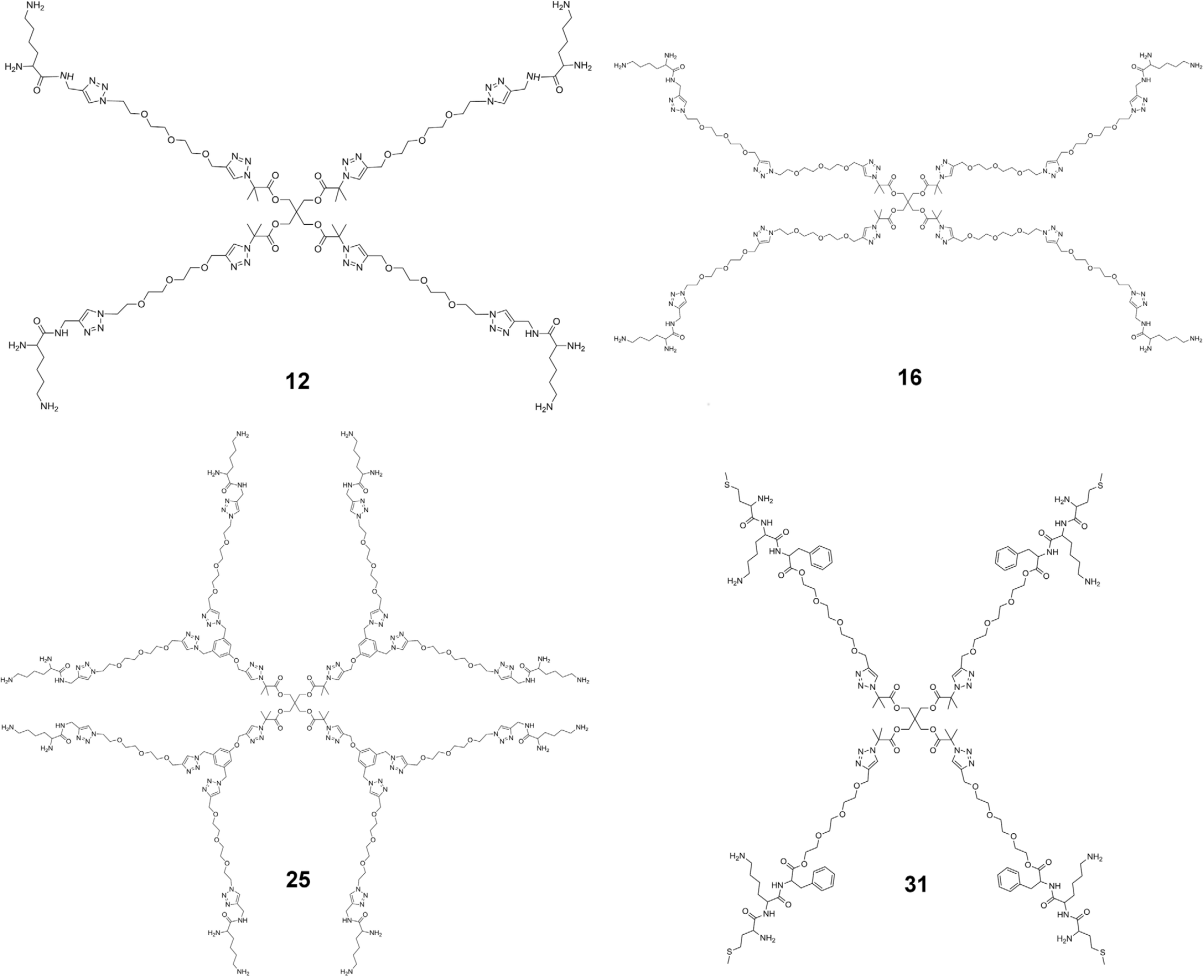
Structure of the 4 peptidomimetics designed and synthesized.

Here we report the *de novo* design of 4-arm star-like peptidomimetic polymers (see Fig 1 for their structures) as potent inhibitors of the voltage-gated K^+^ channel Kv1.3, a target for autoimmune diseases [19, 20]. Stars **12** and **16** comprise of an ethylene glycol (EG) inner core of different lengths and lysine groups on the ends of each arm. Star **25** comprises of EG 8-arms in the second generational layer and with a peripheral layer of lysine groups, whereas star **31** consists of a more hydrophobic triple-amino acid sequence attached to the end-groups of the EG arms. Each structure is designed to bind to certain sites within the ion channel and physically occlude the permeation pathway of Kv1.3. We use potential of mean force (PMF) to predict the binding constants (*K*_d_) of the polymers, and verify this in our subsequent experiments. The work described here highlights the *de novo* design of stable synthetic peptide mimics to interact and inhibit ion channel pathways.

## Methods

### Molecular dynamics

The equilibrated structure of the pore domain of human Kv1.3 channel embedded in a lipid bilayer and a box of explicit water was taken from our previous study [21]. The S2 and S4 ion binding sites of the selectivity filter were occupied by two K^+^ ions, and the S1 and S3 sites by two water molecules, consistent with the crystal structure of a scorpion toxin in complex with a highly-related K^+^ channel [18]. Each star polymer was placed 10 Å above the outer vestibule of Kv1.3 and then docked to the channel using molecular dynamics (MD), in which a flat-bottom distance restraint was applied to slowly pull one of the lysine side chains of the compound into the selectivity filter [22]. The upper bound of the distance restraint, applied to the nitrogen atom of a lysine side chain of the star and the carbonyl group of Gly446 in the filter, was gradually reduced to 3 Å over the first 5 ns. The simulation was then extended to 20 ns, allowing the complex to stabilize.

The PMF profiles were derived using the umbrella sampling method. During the umbrella sampling, the center of mass (COM) of the polymer was confined within a cylinder of 8 Å in radius. The reaction coordinate, *z*, was the COM distance between the channel backbone and the polymer along the channel axis. The PMF profile was constructed using the WHAM method [23] implemented by Grossfield [24]. The *K*_d_ value was computed using the following equation [17]:

$$K_{\text{d}}^{-1}=1000\pi R^{2}N_{\text{A}}\int_{z_{\min}}^{z_{\max}}\exp[-W(z)/kT]\;\text{d}z$$ (1)

where *R* is the radius of the cylinder, *N*_A_ is Avogadro's number, *W(z)* is the PMF at *z*, with *z*_min_ and *z*_max_ being the *z* coordinate when the polymer is fully bound to the channel and in the bulk, respectively. The *z* coordinates were saved every 1 ps (500 steps), such that the autocorrelation function of the successive data points is comparable to 1/*e*. This ensures that the successive data points are well independent, which is required for an unbiased estimate of the error [25]. The random error of the PMF was determined from the bootstrapping method. Specifically, 10 sets of pseudo-data were randomly generated from the original data with duplication allowed. A PMF profile was constructed for each pseudo dataset. The standard deviation of the PMFs as a function of the reaction coordinate was calculated and the maximum value was considered as the uncertainly of the PMF profile. More details of our simulation parameters are given in the S1 File.

### Chemical synthesis

We have recently demonstrated the synthesis of lysine decorated polystyrene molecules that formed micelles with a low aggregation number of ~1 [26]. We used this method to produce four low molecular weight star polymers (**12**, **16**, **25**, and **31**). The four star and dendritic-like compounds were synthesized with the peripheral layer consisting of either lysine or a three amino acid sequence (MKF) as shown in Fig 1. The molecules were all constructed convergently using the copper-catalyzed azide-alkyne cycloaddition (CuAAC) reaction and were designed to consist of a common core, a second generational layer of EG and an outer generational layer with either lysine (stars **12**, **16** and **25**) or the MKF tripeptide (star **31**). All the synthetic schemes are detailed in the S1 File.

### Electrophysiology

The four star molecules synthesized were tested on an electrophysiological platform, using cloned mammalian channels. All the six mammalian K^+^ channels (Kv1.1 Kv1.2, Kv1.3, IK, KCNQ1, and hERG) we tested were expressed in HEK293 cells and used for electrophysiology 1–2 days after transfection. Current measurements and data acquisition were performed with an EPC 10 patch clamp amplifier (HEKA Elektronik, Germany), which was controlled by a Patchmaster software (HEKA Elektronik). Each experiment was replicated at least three times. See the S1 File for further details.

## Results and Discussion

### Structure of Kv1.3

**Fig 2:**
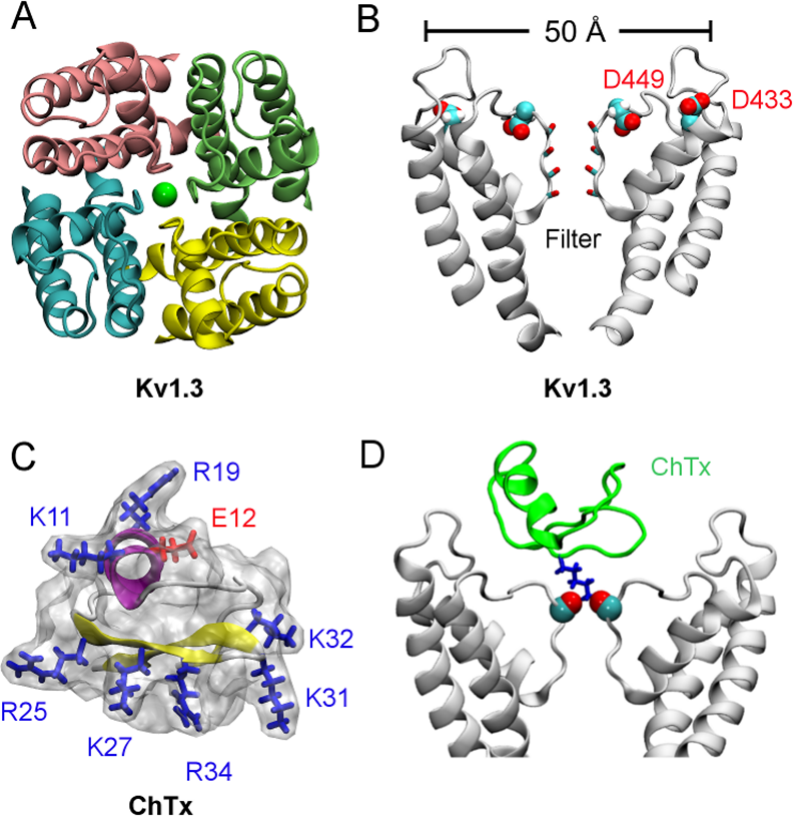
Structure of Kv1.3 and ChTx. In (A) and (B), the view is parallel and perpendicular to the channel axis, respectively. In (A), the green sphere denotes a K^+^ ion in the filter. In (B), only two of the four identical subunits of Kv1.3 are shown. The two helices that extend to the cytoplasmic aspect of the membrane are truncated in the figure. In (C), α-helix of the scorpion toxin ChTx is shown in purple and β-strand in yellow. (D) The crystal structure of ChTx (green) bound to an engineered K^+^ channel (PDB ID 4JTA [18]). The interactions between a lysine residue from the toxin (blue) and two carbonyl groups from the channel filter are highlighted.

The primary structure of the pore domain of Kv1.3 is over 90% identical to that of another voltage-gated K^+^ channel isoform, Kv1.2, whose crystal structure is available [27]. As such, the homology model of the Kv1.3 pore domain could be generated reliably using the structure of Kv1.2 as a template [21]. The pore domain of Kv1.3 as modeled on Kv1.2 shows that a narrow selectivity filter lined by carbonyl groups is positioned in the middle of the homo-tetramer protein (Fig 2A and 2B). The outer vestibule of the channel, with a diameter of approximately 50 Å, carries several rings of acidic residues such as Asp433 and Asp449 (Fig 2B). These acidic residues render the channel susceptible to classical scorpion toxins such as charybdotoxin (ChTx), which potently inhibits several K^+^ channels including Kv1.3 with nanomolar affinities [28]. ChTx consists of a 37-amino-acid peptide carrying seven basic residues and only one acidic residue (Fig 2C). Thus, the positively charged ChTx at neutral pH is attracted by the negatively-charged vestibular wall of Kv1.3. The size of ChTx is in the order of 30–35 Å in each dimension, which fits snuggly with the outer wall of Kv1.3 (Fig 2D).

### Computational design of EG-lysine conjugates

A key lysine residue is common to ChTx and many other scorpion toxins [17]. Both theoretical studies [17] and recent crystallographic data [18] suggest that this lysine residue (K27 for ChTx) protrudes into the extracellular end of the channel filter (Fig 2D), thereby physically occluding the ion permeation pathway. This complex is stabilized by additional hydrogen bonds between the acidic residues lining the vestibular wall of the channel and the basic residues on the toxin. With the common feature of the toxins in mind, we successfully designed and then synthesized four EG-lysine star polymers that mimic the action of ChTx on Kv1.3. The profiles of PMF we constructed predict that two of the compounds would block Kv1.3 at a micromolar affinity.

The EG-lysine conjugate **12** carries four arms, with each arm consisting of three ethylene glycol repeat units and a lysine-like terminus emanating from a 4-arm core structure (Fig 1). The synthetic scheme for **12** is given in Scheme A in S1 File. Each terminus carries two amine groups, and thus two positive charges. The conjugate has an overall charge of +8 at neutral pH. The size of **12** is in the order of 20–40 Å in each dimension, comparable to that of scorpion toxins. The structure of **12** was elongated and flexible (Fig A in S1 File). When simulated in a box of explicit water using MD, the root mean square deviation (RMSD) with reference to the average structure was in the range of 5–11 Å over a simulation period of 30 ns (Fig A in S1 File). This is considerably higher than that of scorpion toxins, which is typically in the range of 1.5–2 Å. Nonetheless, the size and basicity of **12,** and similarly, **16**, **25**, and **31**, resembles that of ChTx and many other scorpion toxins.

**Fig 3:**
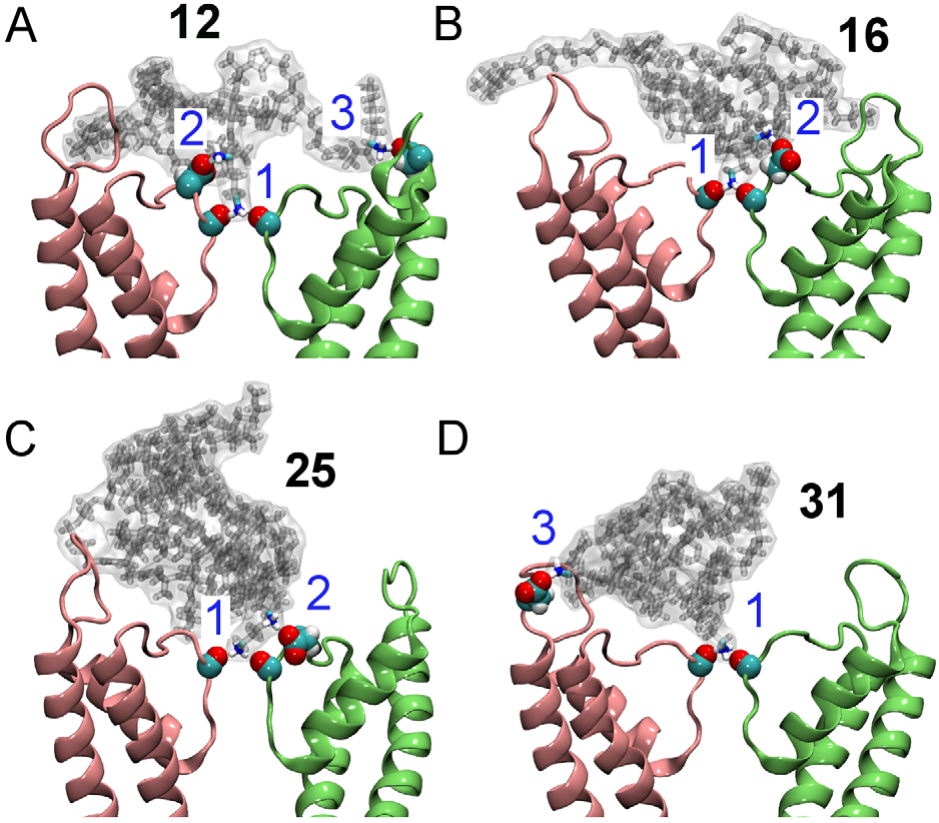
Mimicking the action of scorpion venom peptides using the four peptidomimetic star polymers in Fig 1. The binding modes of the four molecules bound to Kv1.3 as predicted from molecular dynamics are shown. Two channel subunits are shown as pink and lime ribbons. The numbers in blue indicate the three electrostatic contacts between the peptidomimetic and Tyr447 (1), Asp449 (2) and Asp422 (3) of the channel.

Our MD simulations with explicit solvent predicted that all the four compounds block Kv1.3 in a manner similar to that of ChTx (Fig 3). Three strong electrostatic contacts were observed between **12** and the channel (Fig 3). A terminal ammonia group from **12** protrudes into the filter of the channel, forming two hydrogen bonds with the carbonyl groups of Tyr447 in the filter. The second ammonia group from the same arm forms a salt bridge with Asp449 just outside the filter. The third contact is between the amine group of a different arm of the conjugate and Asp422 from the turret of the channel. The interactions of **12** with the filter (Tyr447) and the outer wall (Asp422) of the channel are likely to be crucial for binding, because these two interactions are common to the binding of scorpion toxins to K^+^ channels [17]. On the other hand, the interactions with Asp449, which is much less common, are expected to be less important. Structural changes to the channel filter after the binding of **12** were not evident, consistent with the crystallographic data of Banerjee et al. [18]. The backbone RMSD of the filter (residues 440 to 450) with reference to the starting structure was 0.3 to 0.5 Å over the last 10 ns. Coordination of the two K^+^ ions at the S2 and S4 sites by the backbone carbonyl groups of the filter was maintained.

In the other three complexes, fewer electrostatic contacts were observed. In the case of **16** and **25**, the interactions with Tyr447 and Asp449 in the filter region were present, but no salt bridge involving Asp422 from the channel turret was observed. In the complex between **31** and Kv1.3, the salt bridge involving Asp449 was not evident. The interacting residue pairs observed in the four complexes suggest that **12** should be the strongest inhibitor of Kv1.3, because it forms the most contacts with the channel. On the other hand, **16** and **25** were expected to be the least potent blocker of Kv1.3 as they did not form any salt bridge with the outer wall of the channel.

**Fig 4:**
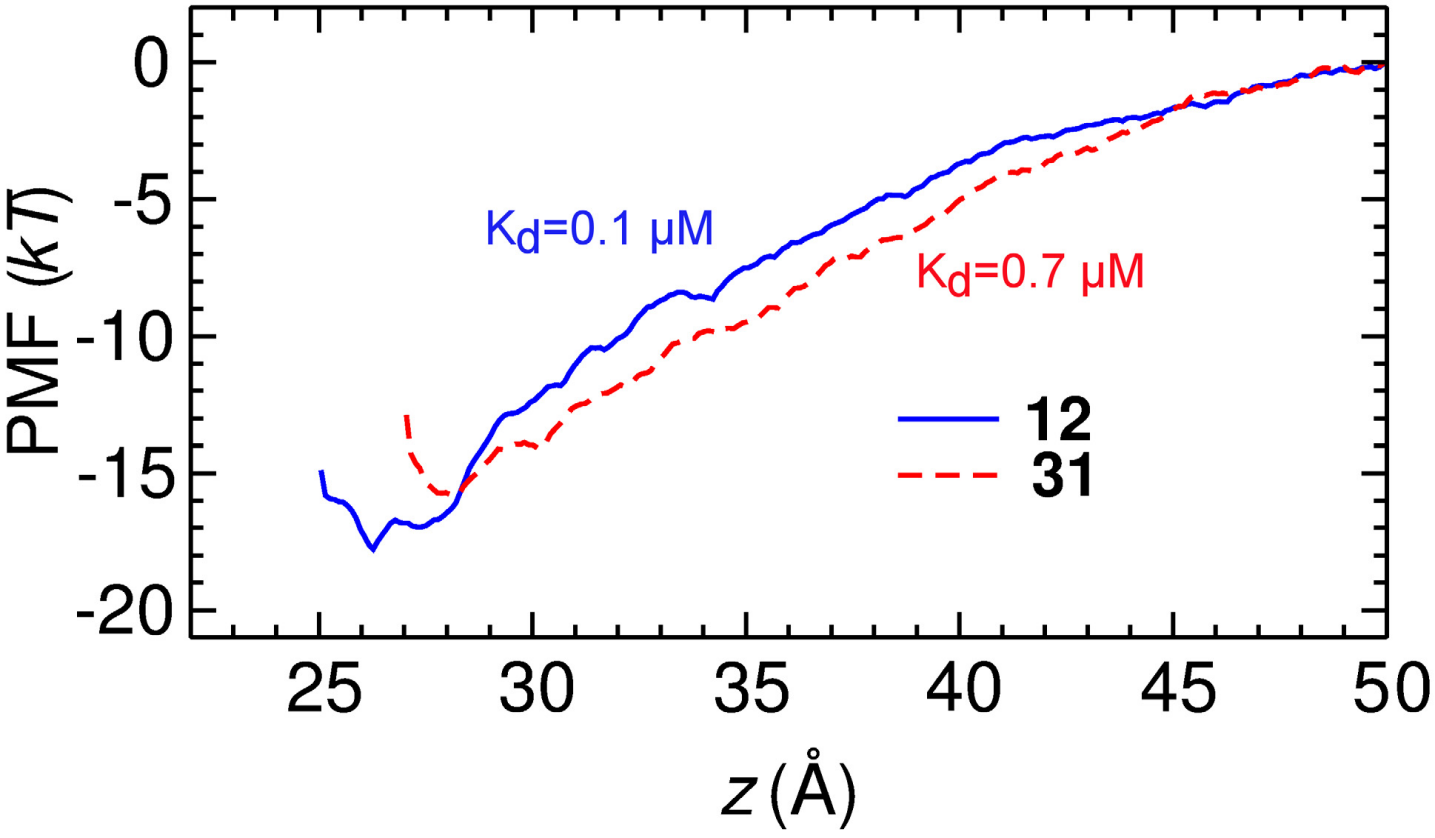
PMF profiles for the binding of compounds 12 and 31 to Kv1.3 as determined from umbrella sampling. The uncertainty of the PMF profiles due to random error is estimated to be 0.4 *kT* in both cases. The reaction coordinate is the centers of mass distance between each compound and the channel backbone in the *z* dimension.

Using PMF calculations, a reliable method for the prediction of toxin affinity as demonstrated both previously [17] and in the present study, we constructed the PMF profiles and the dissociation constants (*K*_d_) of **12** and **31** binding to Kv1.3 (Fig 4). Both profiles converged, as the depth of the profiles did not drift significantly with the simulation time (Fig B in S1 File). The PMF profiles of the two compounds are rather similar, differing by a maximum of 2 *kT* only. By integrating the PMF profiles according to Eq 1, *K*_d_ values of 0.1 μM for **12** and 0.7 μM for **31** were obtained, indicating that our *de novo* designed **12** and **31** are potent blockers of Kv1.3.

### Experimental validation of Kv1.3 block by EG-lysine

**Fig 5:**
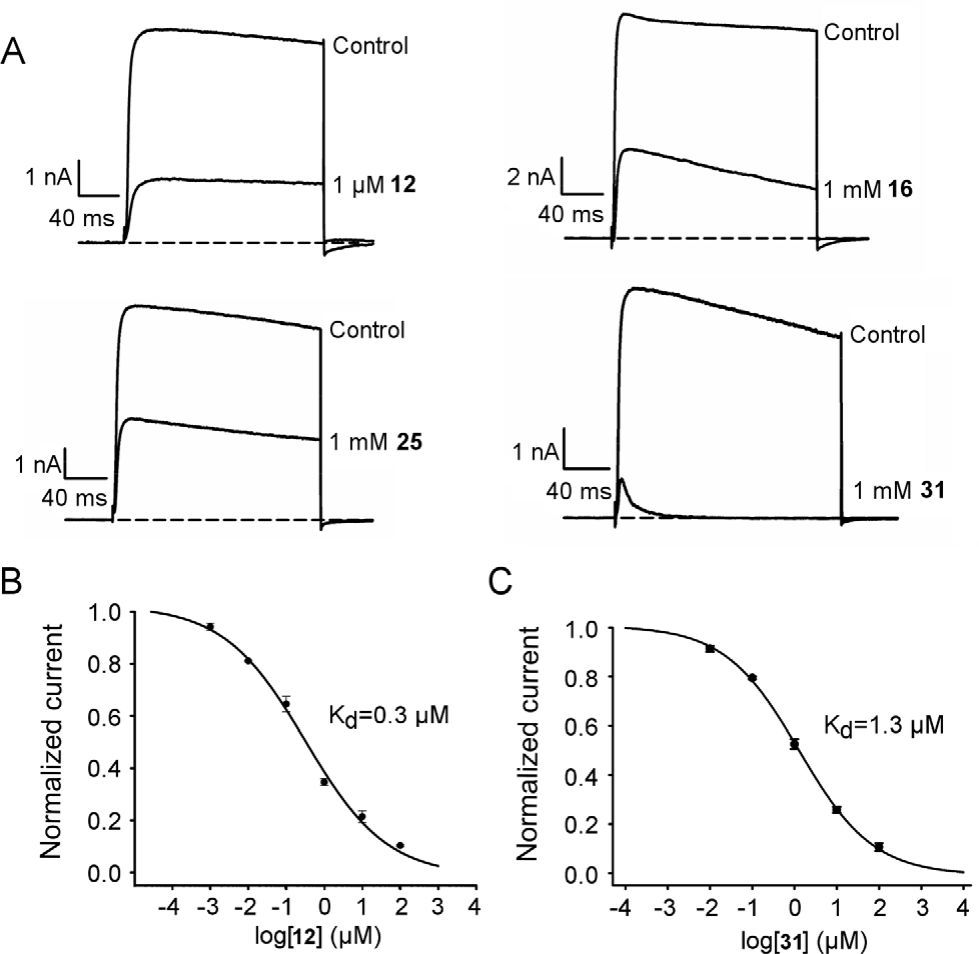
Electrophysiological experiments of the four EG-lysine peptidomimetics. (A) Representative current traces of Kv1.3 showing the attenuation by 1 μM or 1 mM EG-lysine. In (B) and (C), the current-concentration curves of EG-lysine 12 and 31 for Kv1.3 as determined from electrophysiology are shown, respectively.

To validate our computational predictions, electrophysiological measurements on all the four synthesized EG-lysine conjugates were performed. At a concentration of 1 μM, **12** inhibited 65±1% (mean±SEM) of Kv1.3 current (Fig 5A), indicating that **12** is a strong blocker of Kv1.3. In contrast, **16** and **25** did not show strong inhibition on Kv1.3 currents. These two compounds showed inhibition of 62±2% and 59±2% of Kv1.3 currents, respectively, when the concentration was increased to 1 mM. Compound **31** showed stronger inhibition of the Kv1.3 currents than **16** and **25** (83±2% inhibition at 1 mM, Fig 5A). Thus, our initial electrophysiological experiments suggest that **12** and **31** are potent Kv1.3 inhibitors, consistent with the binding modes of these compounds predicted from MD (Fig 3).

In our subsequent experiments, the current-concentration curves of **12** and **31** for Kv1.3 were constructed and the *K*_d_ values derived. The current-concentration curve shows that **12** inhibits 50% of the current at a concentration of 0.3±0.1 μM (Fig 5B), within three-fold to the *K*_d_ value of 0.1 μM predicted from our PMF calculations. Compound **31** inhibited Kv1.3 with a *K*_d_ of 1.3±0.1 μM (Fig 5C), again in good agreement with our calculations (0.7 μM). Although the structure of these compounds is flexible which poses challenges for configurational sampling, the *K*_d_ values predicted from our PMF calculations were in reasonable agreement with the experiments carried out subsequently. This demonstrates that PMF calculations are a reliable method for the prediction of *K*_d_, a central quantity for describing ligand-receptor association.

Specific targeting of ion channels is the key challenge in the development of effective therapeutic agents. The EG-lysine conjugate **12** has the potential to be a therapeutic agent, as it is not only potent but also specific for Kv1.3. At a concentration of 10 μM, **12** inhibited 79±2% of Kv1.3 current and was a much weaker inhibitor for several other K^+^ channel isoforms. The inhibition by 10 μM **12** was 51±2%, 8±2%, 9±1%, 5±1% and 61±2% for Kv1.1, Kv1.2, IK, KCNQ1 and hERG, respectively (Fig C in S1 File). Thus, **12** was most potent for Kv1.3, but was also an effective blocker of hERG, which is critical to the electrical signaling of cardiac myocytes and a target for cardiac arrhythmia [29].

Compound **31** was potent for Kv1.1, KCNQ1 and hERG, but not as potent for Kv1.2 and IK channels. At a concentration of 1 mM, **31** inhibited 77±2%, 70±3%, 88±3% and 83±2% of Kv1.1, KCNQ1, hERG and Kv1.3 currents, respectively (Fig D in S1 File). On the other hand, at the same concentration it only inhibited 57±2% and 36±2% of Kv1.2 and IK currents (Fig D in S1 File). Thus, **31** is a highly potent novel blocker for KCNQ1 and hERG, both of which are insensitive to ChTx.

## Concluding Remarks

Ion channels are implicated in a wide range of diseases, such as hypertension and long QT syndrome [30], and thus have been increasingly regarded as an important drug target [7, 31]. However, the success of early drug development targeting ion channels was limited by the understanding of the diseases at a molecular basis. To date, drug discovery in this area has largely focused on the isolation and modification of naturally occurring venom peptides that are expensive to produce in large quantity [32]. With the structures of various types of ion channels being crystalized, it has become possible to rationally devise compounds aimed at modulating the activity of a specific subfamily of ion channels. By using computational methods it is feasible to design promising lead compounds, which can then be verified experimentally. Moreover, *de novo* design frees one from the need to modify naturally occurring polypeptide-based ion channel modulators. Here we show for the first time that synthetic star molecules can be used as effective and selective channel blockers, whose mechanisms of action are shown to be broadly mirroring those of venom peptides. One of the compounds we designed (compound **12**) has a sub-micromolar affinity for Kv1.3 (*K*_d_ = 0.3 μM) and is more potent than various small drug molecules such as nifedipine (*K*_d_ = 5 μM), diltiazem (*K*_d_ = 27 μM) and resiniferatoxin (*K*_d_ = 3 μM) [28]. It is also selective for Kv1.3 over several other K^+^ channel isoforms, thus providing an excellent template for further development.

The size and shape of EG conjugates can be readily manipulated, and their ends can be functioned with different amino acid sequences. Thus, applying the same principles as those used in the proof-of-concept study reported here, it should be possible to design novel EG-peptide conjugates mimicking peptide blockers of other channels, such as the voltage-gated Na^+^ (Na_V_) and Ca^2+^ (Ca_V_) channels. Na_V_ and Ca_V_ channels are sensitive to conotoxins isolated from cone snail venoms. Several isoforms of Na_V_ and Ca_V_ channels such as Na_V_1.7 and Ca_V_2.2 are well-established targets for the treatment of pain [10, 33]. Novel EG-peptide conjugates specifically constructed to resemble the structure of conotoxins, and mimic their action on Na_V_ and Ca_V_ channels, may prove to be effective analgesics. The compound **31** we designed is selective for hERG, in contrast to the specificity of **12** for Kv1.3, indicating that the specificity profile of the star molecules can be fine-tuned by modifying the sequence of the peptide attached to their ends. Thus, the approach used here of *de novo* design of synthetic peptide mimics can be extended to the drug development of a variety of channels.

## Supporting Information

S1 File Supplementary methods, figures, tables and synthetic schemes are described. (PDF)
