# Supplementary material for: Peptidomimetic Star Polymers for Targeting Biological Ion Channels
Source: PLoS One. 2016 Mar 23;11(3):e0152169. doi: 10.1371/journal.pone.0152169 (PMC4805292; doi:10.1371/journal.pone.0152169)
Supplement: S1 File — (PDF) [file pone.0152169.s001.pdf]

Peptidomimetic Star Polymers for Targeting Biological Ion Channels

Rong Chen,[1*] Derong Lu,[2] Zili Xie,[3] Jing Feng,[3] Zhongfan Jia,[2] Junming Ho,[4] Michelle L. Coote,[4] Yingliang Wu,[3] Michael J. Monteiro,[2*] Shin-Ho Chung[1]

[1]Research School of Biology, Australian National University, Canberra ACT 2601, Australia
[2]Australian Institute for Bioengineering and Nanotechnology, The University of Queensland, Brisbane QLD 4072, Australia
[3]College of Life Sciences, Wuhan University, Wuhan 430072, China
[4]ARC Centre of Excellence for Electromaterials Science, Research School of Chemistry, Australian National University, Canberra ACT 2601, Australia

*Corresponding authors

Emails: rong.chen@anu.edu.au (RC) and m.monteiro@uq.edu.au (MJM)

S1 File

Contents

Supplementary Methods 2

Molecular dynamics 2

PMF calculations 2

Electrophysiology 2

Chemical synthesis: materials 3

Chemical synthesis: methods 4

Supplementary Tables 7

Synthetic Schemes 8

Supplementary Figures 34

References 58

Supplementary Methods

Molecular dynamics

All molecular dynamics (MD) simulations were performed under periodic boundary conditions using NAMD 2.9 [1]. The CHARMM36 force fields for lipids [2] and proteins [3] and the TIP3P model for water [4] were used to describe the interatomic interactions in the system. The parameters of EG-lysine were taken from those of lipids and proteins. Specifically, the core and the ethylene glycol repeats were taken from lipids and the terminal lysine from the lysine amino acid. The switch and cutoff distances for short-range interactions were set to 8.0 Å and 12.0 Å, respectively. The long-range electrostatic interactions were accounted for using the particle mesh Ewald method, with a maximum grid spacing of 1.0 Å. Bond lengths were maintained rigid with the SHAKE [5] and SETTLE [6] algorithms. A time step of 2 fs was used. The temperature and pressure were maintained constant at 300 K on average by using the Langevin dynamics (damping coefficient 1 ps^{-1}), and an average of 1 atm by using Nosé-Hoover Langevin Piston method [7], respectively. The barostat oscillation and damping time scale were set to 200 ps and 100 ps, respectively. The pressure coupling was semiisotropic. Trajectories were saved every 20 ps for analysis.

PMF calculations

PMF calculations were performed using umbrella sampling. The starting structures of the umbrella windows spaced at 0.5 Å intervals were generated by moving EG-lysine along the reaction coordinate (*z*) from the fully-bound position to the extracellular space. The reaction coordinate was the center of mass (COM) distance between the heavy atoms of EG-lysine and the backbone of Kv1.3 along the bilayer normal. The biasing potential of each window was 30 kcal/mol/$Å^2$. A flat-bottom harmonic restraint was applied to maintain the COM of EG-lysine within a cylinder of 8 Å in radius centered on the central channel axis. The restraining potential was always zero when EG-lysine was fully bound to the channel.

Electrophysiology

The pRc/CMV-Kv1.3 vector, generously provided by Prof. Stephan Grissmer (University of Ulm, Ulm, Germany) and Prof. Olaf Pongs (Zentrum für MoLekulare Neurobiologie der Universität Hamburg, Hamburg, Germany), was used directly. The cDNAs encoding human Kv1.2, IKCa, hERG and KCNQ1 channels (from Prof. Stephan Grissmer, University of Ulm, Ulm, Germany and Prof. George Chandy, University of California, Irvine, CA) were subcloned into the vector of

pIRES2-EGFP (Clontech, USA) for coexpression with GFP. Plasmids containing each of the six channels were subsequently transfected into HEK293 cells using the SofastTM Transfection Reagent (Sunma). HEK293 cells were cultured in Dulbecco's modified Eagle's medium (Invitrogen), and incubated at 37 ℃ with 10% fetal bovine serum, 1% penicillin/streptomycin. Cells were transfected using FuGENE transfection reagent (Roche Diagnostics) and used for electrophysiology 1-2 days after transfection. Electrophysiological experiments were performed at 22-25 ℃ using the patch clamp whole cell recording mode. Current measurements and data acquisition were performed with an EPC 10 patch clamp amplifier (HEKA Elektronik, Germany) controlled by a Patchmaster software (HEKA Elektronik). For Kv1.1, Kv1.2, Kv1.3, KCNQ1 and hERG channels, the bath contained 5 mM KCl, 140 mM NaCl, 10 mM HEPES, 2 mM $CaCl_2$, 1 mM $MgCl_2$, 10 mM D-glucose. The pipette solution contained 140mM KCl, 1 mM $MgCl_2$, 1 mM EGTA, 1 mM Na_2ATP, 5 mM HEPES. For the IK channel, the bath contained 130 mM sodium aspartate, 30 mM potassium aspartate, 2 mM $CaCl_2$, 1 mM $MgCl_2$, and 10 mM HEPES. The pipette solution contained 145 mM potassium aspartate, 8.7 mM $CaCl_2$, 2 mM $MgCl_2$, 10 mM EGTA, and 10 mM HEPES to achieve an intracellular free Ca^{2+} concentration of 1 μM. The pH of the bath and pipette solutions was adjusted to 7.4 and 7.2 using NaOH and KOH, respectively. Kv1.1, Kv1.2 and Kv1.3 channel currents were elicited by depolarizing voltage steps of 200 ms and KCNQ1 channel currents were elicited by depolarizing voltage steps of 1s from the holding potential −80 mV to +50 mV. The sweep intervals were 10 s for Kv1.1, Kv1.2, KCNQ1, hERG, and 30 s for Kv1.3 channels. For the IK channel, the membrane potentials were clamped to -120 mV for 50 ms (which was used for the current measurements) followed by a 400 ms voltage ramp from -120 mV to +60 mV and were kept for 5 s between ramps at -40 mV. Data analyses were performed with IGOR software (WaveMetrics, Lake Oswego, OR). K_d values were obtained by fitting a modified Hill equation as displayed below:

$$I_{\text{drug}} / I_{\text{control}} = 1/(1+[\text{drug}]/K_{\text{d}})$$

where I is the peak currents and [drug] is the concentration of drug. Results are shown as the mean ± S.E. of at least three experiments.

Chemical synthesis: materials

The following chemicals were used as received: alumina, activated basic (Aldrich, Brockmann I, standard grade, ~150 mesh, 58 Å), magnesium sulfate ($MgSO_4$: anhydrous, Scharlau, extra pure), sodium chloride (NaCl: Univar, 99.9%), sodium iodide (NaI: Aldrich, 99.5%), sodium azide (NaN_3: Aldrich, 99.5%), 1,1,1-triisopropylsilyl chloride (TIPS-Cl: Aldrich, 99%), ethylmagnesium bromide solution (EtMgBr, Aldrich, 3.0 M in diethyl ether), triethylamine (TEA: Fluka, 98%), TLC plates

(silica gel 60 F254), silica gel 60 (230-400 mesh ATM (SDS)), potassium carbonate (K_2CO_3, analaR, 99.9%), 2-bromoisobutyryl bromide (BIB, Aldrich, 98%), lithium aluminium hydride ($LiAlH_4$, Aldrich, 98%), diphenyl phosphoryl azide (DPPA, Aldrich, 97%), 1,8-diazabicylco[5,4,0]undec-7-ene (DBU, Aldrich, 98%), tetrabutylammonium fluoride hydrate (TBAF, Aldrich, 1.0 M in THF), 18-crown-6 ether (18-C-6, Aldrich, 99%), imidazole (Aldrich, 99%) *N*,*N′*-dicyclohexylcarbodiimide (DCC, Aldrich, 99%), pentaerythritol (Aldrich, 99%), 2-(2-(2-chloroethoxy)ethoxy)ethanol (Aldrich, 96%), 4-(Dimethylamino)pyridine (DMAP, Aldrich, >99%), propargyl bromide (Aldrich, 80wt% in toluene), propargyl amine (Aldrich, 98%), triethlyene glycol (Aldrich, 98%), di-*tert*-butyl dicarbonate (Boc, Aldrich, >99%), L-lysine (Lys, Aldrich, >98%), trifluoroacetic acid (TFA, Aldrich, 99%). The custom peptide Boc-Met-Lys(Boc)-Phe-OH (Boc-MKF-OH) was purchased from Auspep Pty Ltd, the peptide was purified by HPLC chromatogram, and used as received.

The following solvents were used as received: acetone (ChemSupply, AR), dimethyl sulfoxide (DMSO: Labscan, AR grade), dichloromethane (DCM: Labscan, AR grade), ethyl acetate (EtOAc: ChemSupply, AR grade), methanol (MeOH: anhydrous, Lichrosolv, 99.9%, HPLC grade), *N*,*N*-dimethylacetamide (DMAc: Aldrich, HPLC grade), petroleum spirit (BR 40-60 ^{o}C, Univar, AR grade), tetrahydrofuran (THF: Lichrosolv, HPLC grade), and toluene (TOL, Univar, AR grade).

The following initiators, ligands, and metals for the various polymerizations are given below and used as received unless otherwise stated: *N*,*N*,*N*’,*N*’’,*N*’’-pentamethyldiethylenetriamine (PMDETA: Aldrich, 99%), Copper(I)bromide were synthesized in the Monteiro group.

Chemical synthesis: methods

12 was constructed by first coupling through the CuAAC reaction **2** (EG-N_3) and **4** (Lys-≡) to form **5** (Scheme A in S1 File) in a 79% yield. The HO-group on **5** was converted via an etherification reaction to the alkyne **6** in a 27.8% yield. The CuAAC of **6** and **10** (tetra azide core) produced **11** with a purity of 60.9% as determined from the log-normal distribution (LND) simulations [8, 9] of the size exclusion chromatography (SEC) trace (Fig J in S1 File). There was a considerable amount of 3-arm (25.4%), 2-arm (9.1%) and a small amount of 1-arm and unreacted core (**10**). Upon purification of crude **11** by preparative SEC (Fig J in S1 File), 95% of the product consisted of **11** and the other 5% consisted of 3-arm. This was further supported from the MALDI-ToF data, in which the products before purification were in agreement with the LND fit (Fig J in S1 File) and after purification (Fig J in S1 File) the product consisted of predominantly **11** (i.e. **11** + Na^{+}) showed one mass peak at 2986.84 and a small peak at 2389.08 (corresponding to the 3-arm product).

The structure of **11** was further confirmed by the ^{1}H NMR in Fig K in S1 File. The Boc-protecting groups on **11** were then removed using TFA for 6 h at room temperature to give **12**.

16 was designed with a longer spacer (i.e. two EG units per arm) within the second generational layer (Scheme C in S1 File). Compound **13** was synthesized through the CuAAC coupling of **6** and **2** to produce **13**, and then conversion of the HO-group on **14** to an alkyne (see Schemes C in S1 File). The CuAAC reaction between **14** and **10** afforded **15** (crude) in quite a low purity of 35.6 % as determined by the LND fit (Fig N in S1 File). The majority of the impurity was due to **14** (45%), which was used in excess to the azide core **10** (6.3% remaining). There was only a small amount of 1, 2 and 3-arm products, suggesting the excess of **14** drove the reaction to the 4-arm product **15**. After preparative SEC, the purity of **15** increased to 97% with only 3% of the 3-arm product remaining. The MALDI-ToF data confirmed the LND simulations, demonstrating that after preparative SEC the main product consisted of **15** (where M=**15**+Na^+=3841.16) as shown in Fig N in S1 File. The integration of the ^{1}H NMR also showed that **15** consisted of high purity (Fig O in S1 File). Deprotection of Boc-groups on **15** gave **16** with near quantitative conversion.

25 was designed with twice the number of lysine groups in the outer generational layer (Scheme D in S1 File). To accomplish this, the CuAAC reaction of **6** and **21** gave **22** followed by deprotection of Tips by TBAF and obtained as **23** after purification of 49%. **24** was formed through the CuAAC reaction of **10** with excess **23** with a yield of 29.9%. The LND simulation of the SEC traces in Fig R in S1 File showed that **24** was formed with a purity of 43%, with impurities arising from the starting compounds **10** and **23**, and the formation of 1-arm to 3-arm star products. After preparative SEC, the purity increased to 94% with 3% of both 2- and 3-arm star products. This was consistent with the MALDI-ToF data, in which one main mass peak was observed at 6344.96 corresponding to **24** with the Na ion (Fig R in S1 File). Once again the ^{1}H NMR also showed that **24** was produced with a high purity (Fig S in S1 File). Deprotection of Boc-groups on **24** using TFA gave **25** with near quantitative conversion.

31 was designed so that the peripheral layer consisted of a three amino acid sequence of Met-Lys-Phe (MKF) (Scheme E in S1 File). The coupling of **28** (OH-MKF-Boc) with **27** afforded **29** in a yield of 46%. The CuAAC reaction between **10** and excess **29** gave **30** with a purity of 73% (Fig V in S1 File). The impurities consisted of the starting compounds and formation of 1-arm to 3-arm star products. After preparative SEC, the purity increased to 97% with 3% of 3-arm star remaining. The MALDI-ToF further confirmed the high purity with only one major peak after preparative SEC (Fig V in S1 File) shows that a number of species were formed after ionization through the MALDI. These species were consistent with the various oxidized species from the sulfur on the methionine

group (Fig V in S1 File). Deprotection of Boc-groups on **30** using TFA gave **31** with near quantitative conversion.

Analytical Methodologies

Size Exclusion Chromatography (RI-SEC)

All polymer samples were dried prior to analysis in a vacuum oven for 2 days at 25 ^{o}C. The dried polymer was dissolved in tetrahydrofuran (THF) to a concentration of 1 mg mL^{-1} and then filtered through a 0.45 μm PTFE syringe filter. The molecular weight distributions of the polymers was determined through separation on a Waters 2695 separations module, fitted with a Waters 410 refractive index (RI) detector maintained at 35 ^{o}C, a Waters 996 photodiode array detector, and two Ultrastyragel linear columns (7.8 x 300 mm) arranged in series. These columns were maintained at 40 ^{o}C for all analyses and are capable of separating polymers in the molecular weight range of 500 to 4 million g mol^{-1}with high resolution. All samples were eluted at a flow rate of 1.0 mL min^{-1}. Calibration was performed using narrow molecular weight PSTY standards ($PDI_{RI} \leq 1.1$) ranging from 500 to 2 million g mol^{-1}. Data acquisition was performed using Empower software, and molecular weights were calculated relative to polystyrene standards.

Nuclear Magnetic Resonance (NMR)

All NMR spectra were recorded on either a Bruker DRX 400 or 500 MHz spectrometer using an external lock ($CDCl_3$), and all spectra were referenced to the residual nondeuterated solvent ($CHCl_3$). The frequencies were 400 or 500 MHz for ^{1}H NMR spectra, and 100 or 125 MHz for ^{13}C NMR spectra.

Matrix-Assisted Laser Desorption Ionization-Time-of-Flight (MALDI-ToF) Mass Spectrometry

MALDI-ToF MS spectra were obtained using a Bruker MALDI-ToF autoflex III smart beam equipped with a nitrogen laser (337 nm, 200 Hz maximum firing rate) with a mass range of 600-400 000 Da. Spectra were recorded in either reflectron mode (1500-4500 Da) or linear mode (4000-20000 Da). Trans- 2-[3-(4-tert- butylphenyl)-2-methyl-propenylidene] malononitrile (DCTB; 20 mg mL^{-1}in THF) was used as the matrix and Na(CF_3COO) (1 mg mL^{-1} in THF) as the cation source for all the polystyrene samples. The 20 μL sample solution (0.5 mg mL^{-1} in THF), 20 μL DCTB solution and 2 μL Na(CF_3COO) solution were mixed in an eppendorf tube, vortexed and centrifuged. A 1 μL of solution was placed on the target plate spot, evaporated the solvent at ambient condition and run the measurement.

Supplementary Tables

Table A. IC_{50} values of six toxin-channel systems computed and determined experimentally

Toxin	Channel	IC_{50} (nM)		References
		Computed	Experiment	
OSK1	Kv1.3	0.02	0.01	[10]
ShK	Kv1.3	0.17	0.13	[10]
ChTx	Kv1.3	25	2.6	[10]
PIIIA	NavAb	0.02	0.005	[11, 12]
Css4	$Na_V1.2$	20	34	[13]
Cn2	$Na_V1.6$	70	40	[13]

Table B. SEC and MALDI-ToF MS data for all starting materials and dendrimers

Compound	$M_{n,RI}$ [a]	$M_{p,RI}$ [a]	PDI	$M_{w,theo}$ [b]	ΔHDV [c]	$M_{n,MALDI}(+Na^+)$ [d]	Purity % [e]
**6**	410	430	1.03	597	0.693		
**13**	510	515	1.02	809	0.627		
**26**	670	710	1.03	795	0.843		
**10**	430	440	1.03	581	0.737		
**11** [f]	2220	2370	1.03	2964	0.763	2986.84	95 %
**15** [f]	2610	2660	1.03	3820	0.683	3841.97	97%
**23**	1220	1180	1.05	1436	0.848		
**24** [f]	3870	3930	1.03	6323	0.512	6344.96	94%
**30** [f]	3380	3480	1.02	3759	0.899	3782.89	97%

[a] SEC (RI detector) was based on a polystyrene calibration curve; [b] Theoretical molecular weight; [c] Hydrodynamic volume change (ΔHDV = $M_{n,RI}/M_{w,theo}$); [d] Molecular weight determined by MALDI-ToF MS; [e] Determined from log-normal distributions(LND) simulation. [f] Purified by prep-SEC.

Synthetic Schemes

Scheme A. Synthesis of EG-Lysine **12**

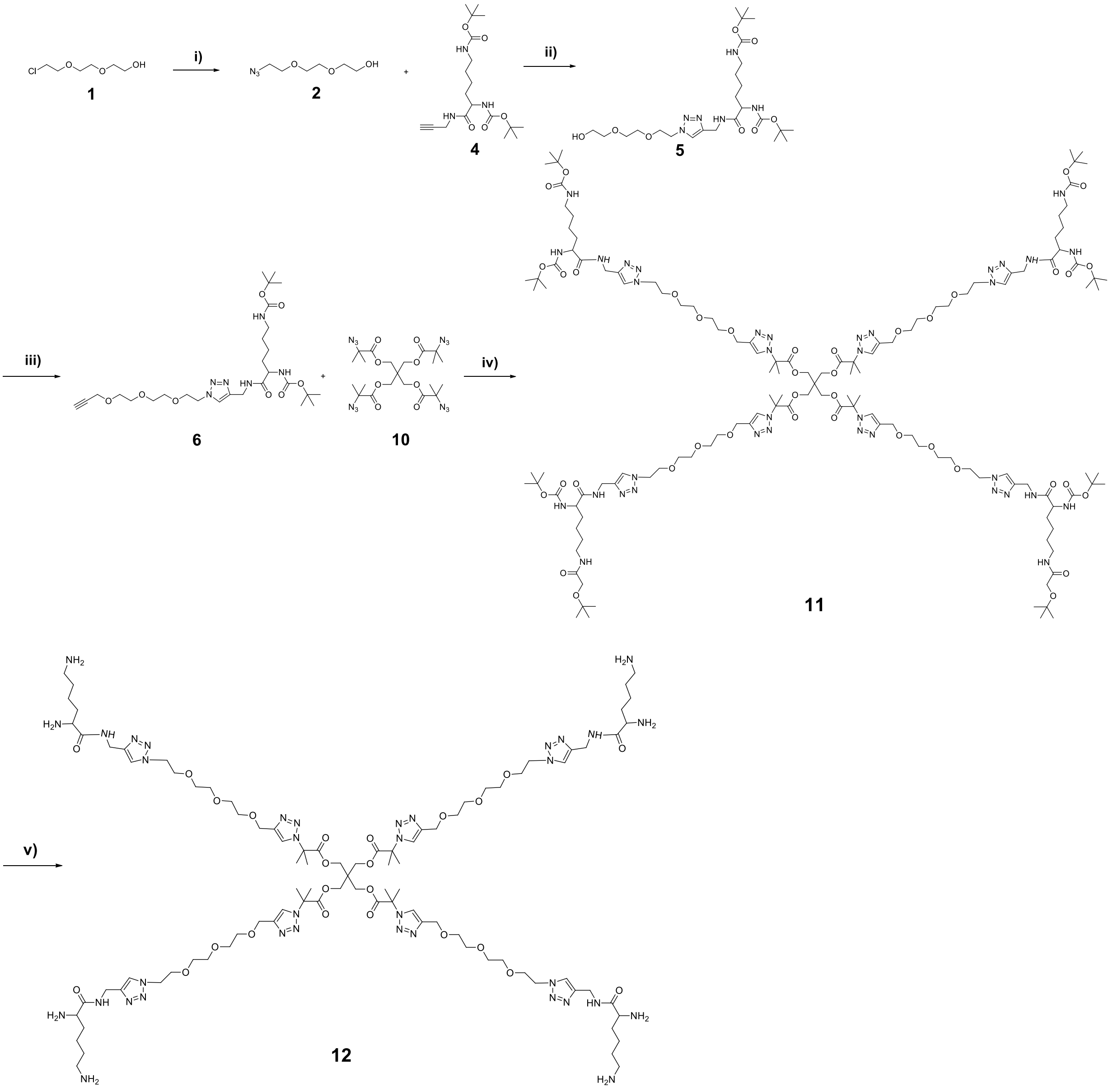

(i) NaN_3, NaI, H_2O, 60 ^oC, 16h; (ii) CuBr, PMDETA, Toluene, DMSO, 25 ^oC, 30 min; (iii) THF, NaH, propargyl bromide, -78 ^oC, 16 h; (iv) CuBr, PMDETA, Toluene, 25 ^oC, 120 min; (v) TFA, DCM, R.T., 6h.

Synthesis of 2-(2-(2-azidoethoxy)ethoxy)ethanol (2)

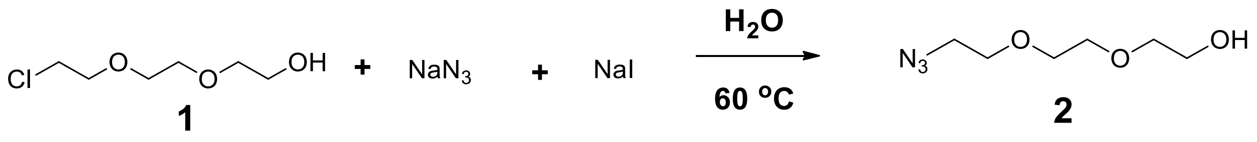

A mixture of 2-(2-(2-chloroethoxy)ethoxy)ethanol (**1**, 5 g, 2.976 x 10^{-2} mol), NaN_3 (19.3 g, 2.976 x 10^{-1} mol), NaI (0.9 g, 6.0 x 10^{-3} mol) and H_2O (30 mL) was added into a 100 mL flask and was then placed into a 60 °C oil bath. The reaction was allowed to react for 16 h with stirring. After removal of the solvent under reduced pressure, the product was diluted in 50 mL DCM, washed with saturated brine (2 x 20 mL) and the organic phase was dried over anhydrous $MgSO_4$. The solvent was concentrated under reduce pressure. 4.47 g viscous oil **2** was obtained with the yield of 86% and used for next step directly without further purification.

^{1}H NMR ($CDCl_3$, 298K, 500 MHz): δ 3.65 (t, 2H, *J*=4.52 Hz, -C***H**₂*OH), 3.59 (s, 6H, -C***H**₂*-), 3.58 (t, 2H, *J*=4.52 Hz, -C***H***$_2$CH$_2$OH), 3.37 (t, 2H, *J*=5.00 Hz, -C***H***$_2$N$_3$), 1.96 (bd, 1H, -O***H***), ^{13}C NMR ($CDCl_3$, 298K, 125 MHz): 50.67, 61.68, 70.03, 70.39, 70.65, 72.62.

Synthesis of alk-Lysine-Boc (4)

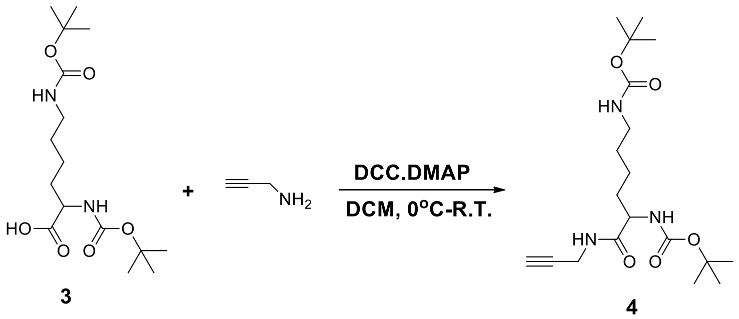

Boc-Lysine-OH (**3**) lysine was synthesized according to reference [14]. To a 500 mL flask, **3** (6.0 g, 0.172 mol), propargyl amine (1.90 g, 0.0345 mol), and DMAP (0.316 g, 2.59 x 10^{-3} mol) were dissolved in 120 mL dry DCM and cooled to 0 °C in an ice-bath. A mixture of DCC and 50 mL DCM was added dropwise into the solution over 30 min. The mixture was allowed to react for 36 h at room temperature. The solid content was removed by filtration and the filtrate was washed by saturated brine (2 x 50 mL). The organic layer was collected, dried over anhydrous $MgSO_4$, the solvent removed *in vacuo* followed by column chromatography using ethyl acetate/petroleum spirit (3/1, v/v, R_f=0.65) as the eluent. Product **4** was obtained as a white solid (2.86 g, yield%=41.7 %). ^{1}H NMR ($CDCl_3$, 298K, 500 MHz): δ 6.91 (s, 1H, CHCCH$_2$N***H***CO-), 5.32 (s, 1H, -CHN*H*CO-), 4.69 (s, 1H, -CH$_2$N***H***CO), 3.97-4.07 (b, 3H, CH$_2$CC***H***$_2$NH- and –CH$_2$C***H***CO-), 3.08 (t, 2H, *J*=6.6 Hz, -CH$_2$C***H***$_2$NH-), 2.20 (t, 1H, *J*=2.5 Hz, ***H***CCCH$_2$-), 1.2-2.0 (m, 24H, C***H***$_2$-Lys and C***H***$_3$-Boc). ^{13}C NMR ($CDCl_3$, 298K, 125 MHz): 22.69, 28.44, 28.53, 29.09, 29.70, 32.23, 40.07, 54.24, 71.60, 79.16, 79.53, 80.10, 155.97, 156.26, 172.22.

Synthesis of OH-EG-Lysine-Boc (5)

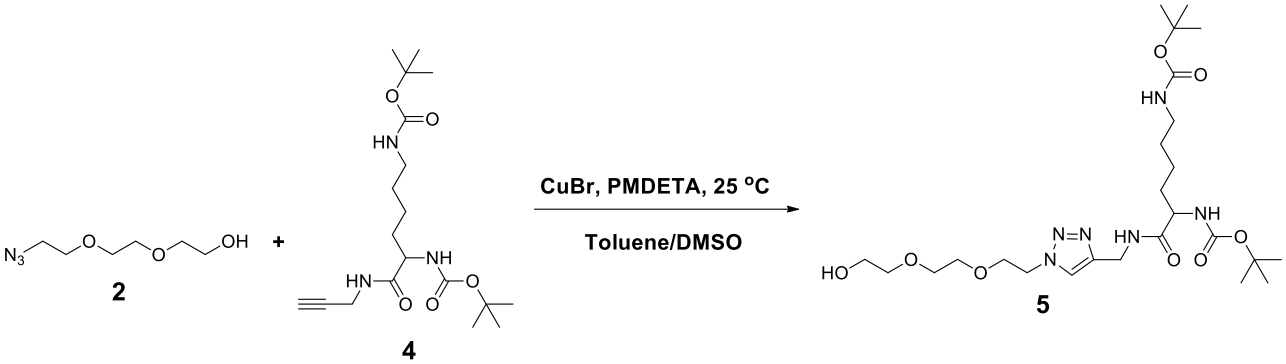

2 (0.684 g, 3.909 x 10^{-3} mol) and **4** (0.650 g, 1.699 x 10^{-3} mol) were placed in a Schlenk tube and dissolved in a mixture of PMDETA (0.0293 g, 1.70 x 10^{-4} mol), toluene (4 mL), DMSO (2 mL). Oxygen was removed from the solution by purging with argon for 30 min. Cu(I)Br (0.024 g, 1.70 x 10^{-4} mol) was added under a positive argon flow and the solution was purged with argon for another 5 minute. The reaction vessel was then sealed and placed in an oil bath at 25 °C and kept stirring for 30min. The mixture was diluted in DCM and passed through activated basic alumina. The solvent was removed under reduced pressure, and the residue was purified by column chromatography with DCM-MeOH (6:1, v/v, R_f=0.55) as eluent. 2.26 g viscous oil **5** was obtained with the yield as 79%.

^{1}H NMR ($CDCl_3$, 298K, 500 MHz): δ 7.85 (s, 1H, -NC***H***C-, triazole ring proton), 7.48 (bd, 1H, -CCH_2N***H***CO-), 5.40 (bd, 1H, -CHN***H***C(O)O-), 4.77 (bd, 1H, -CH_2N***H***C(O)O-), 4.47-4.48 (m, 4H, -CH_2C***H***$_2$N-, -CC***H***$_2$NH-), 4.05 (s, 1H, -C(O)C***H***(CH_2)NH-), 3.81 (t, 2H, *J*=4.90 Hz, -COC***H***$_2CH_2$N-), 3.69 (t, 2H, *J*=4.58 Hz, OHC***H***$_2CH_2$O-), 3.57 (bd, 4H, -OC***H***$_2CH_2$O-, -OCH_2C***H***$_2$O-), 3.51 (t, 2H, *J*=4.55 Hz, $OHCH_2C$***H***$_2$O-), 3.44 (bd, 1H, -O***H***), 3.02 (t, 2H, *J*=6.6 Hz, -CH_2C***H***$_2$NH-), 1.2-2.0 (m, 24H, C***H***$_2$-Lys and C***H***$_3$-Boc). ^{13}C NMR ($CDCl_3$, 298K, 125 MHz): 22.68, 28.40, 28.52, 29.64, 32.32, 34.85, 40.08, 50.38, 54.48, 61.64, 69.21, 70.21, 70.51, 72.60, 79.17, 79.99, 124.03, 144.54, 155.86, 156.30, 172.66.

Synthesis of alk-EG-Lysine-Boc conjugate (6)

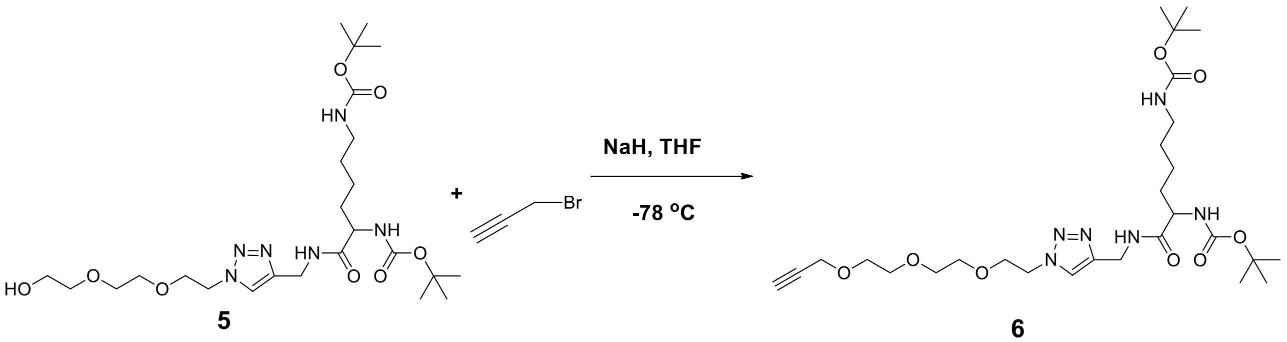

5 (0.6 g, 1.075 x 10^{-3} mol) was dissolved in 10 mL dry THF in a 25 mL two-neck round bottom flask, the flask was connected to the argon line and the solution was cooled to 0°C in an ice-bath. NaH (0.043 g, 1.075 x 10^{-3} mol) (60 % in mineral oil) was added proportionally in the above solution in 5 min. The reaction was allowed stirring for 1 h and there was no bubbling if stop the argon. The reaction vessel was then cooled down to -78 °C in dry ice/acetone mixture. Propargyl bromide (0.237 g, 1.6 x 10^{-3} mol) (80 wt % in toluene) was added to the solution dropwise in 10 min. The reaction was then kept stirring overnight and warmed to RT. The reaction mixture was filtered to remove the salt and concentrated to remove all the solvent and low b.p impurities at RT. The crude brown liquid product was purified by column chromatography with DCM-MeOH (10:1, v/v, R_f=0.45) as eluent. 0.21 g viscous oil **6** was obtained with the yield as 27.8 %.

^{1}H NMR ($CDCl_3$, 298K, 500 MHz): δ 7.72 (s, 1H, -NC***H***C-, triazole ring proton), 6.95 (bd, 1H, -CCH_2N***H***CO-), 5.18 (bd, 1H, -CHN***H***C(O)O-), 4.63 (bd, 1H, -CH_2N***H***C(O)O-), 4.51-4.49 (m, 4H, -CH_2C***H***$_2$N-, -CC***H***$_2$NH-), 4.17 (d, 2H, *J*=2.5 Hz, CHC***H***$_2$O-), 4.05 (s, 1H, -C(O)C***H***(CH_2)NH-), 3.83 (t, 2H, *J*=4.90 Hz, -COC***H***$_2$$CH_2$N-), 3.67 (m, 2H, OHC***H***$_2$$CH_2$O-), 3.62 (m, 2H, $OHCH_2C$***H***$_2$O-), 3.59 (bd, 4H, -OC***H***$_2$$CH_2$O-, -$OCH_2C$***H***$_2$O-), 3.05 (t, 2H, *J*=6.6 Hz, -CH_2C***H***$_2$NH-), 2.43 (t, 1H, *J*=2.3 Hz, C***H***CCH_2-), 2.0-1.2 (m, 24H, C***H***$_2$-Lys and C***H***$_3$-Boc). ^{13}C NMR ($CDCl_3$, 298K, 125 MHz): 22.68, 29.76, 32.31, 34.98, 40.05, 50.37, 54.52, 58.48, 69.18, 69.47, 70.45, 70.56, 70.63, 74.86, 79.19, 79.66, 80.05, 123.48, 144.45, 155.82, 156.30, 172.36.

Scheme B. Synthetic route for tetrafunctional core **(10)**

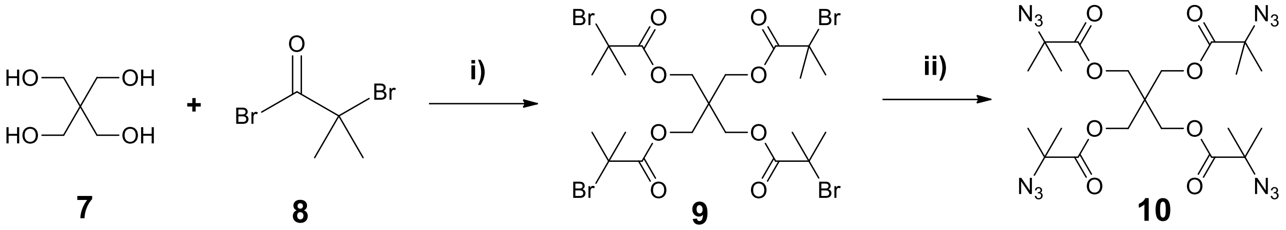

i) THF, TEA, 0°C-R.T., 6 h. ii) NaN_3, DMF, 24 h.

Synthesis of tetra bromo core (9)

A solution containing 2-bromoisobutyryl bromide (**8**, 25.1 g, 0.109 mol) and 80 mL of dry THF was added dropwise to another solution containing pentaerythritol (**7**, 3.0 g, 2.2 x 10^{-2} mol), triethylamine (11.1 g, 0.109 mol) and 220 mL of dry THF at 0 °C. The reaction was allowed to stir for 6 h, filtered to remove the salts, and then the filtrate concentrated by rotary evaporation. The resulted product was dissolved in 300 mL ether and sequentially washed with 10 wt% HCl, saturated $NaHCO_3$ solution, and brine. The organic layer was collected, dried over anhydrous $MgSO_4$ and filtered. The filtrate was concentrated by rotary evaporation. The concentrate was purified by silica gel column chromatography using ethyl acetate/petroleum spirit (1/6, v/v) as the eluent. Product **9** was obtained as white crystals (10.65 g, 66.0 %). R_f (1/6 EtOAc/petroleum spirit) 0.61.

^{1}H NMR ($CDCl_3$, 298K, 400 MHz): δ 1.92 (s, 24H, C***H***$_3$-), 4.30 (s, 8H, -OC***H***$_2$C-). ^{13}C NMR ($CDCl_3$, 298K, 100 MHz): 30.62, 43.64, 55.18, 62.88, 170.89.

Synthesis of tetra azide core (10)

NaN_3 (1.77 g, 2.73 x 10^{-2} mol) was added to a stirred solution of **9** (1.00 g, 1.37 x 10^{-3} mol) in 6 mL of DMF. The reaction mixture was stirred for 24 h at room temperature. The resulted mixture was diluted by 30 mL DCM and passed through short basic alumina column to remove NaN_3. The filtrate was concentrated by rotary evaporation, and dried under high vacuum for 48 h at 25 °C obtained as light yellow powder **10**. The resulted product was used for next step without further purification.

^{1}H NMR ($CDCl_3$, 298K, 500 MHz): δ 1.48 (s, 24H, C***H***$_3$-), 4.27 (s, 8H, -OC***H***$_2$C-). ^{13}C NMR ($CDCl_3$, 298K, 125 MHz): 24.47, 43.09, 62.60, 63.33, 172.18.

Synthesis of 4-arm star-like EG-Lysine-Boc conjugate (11)

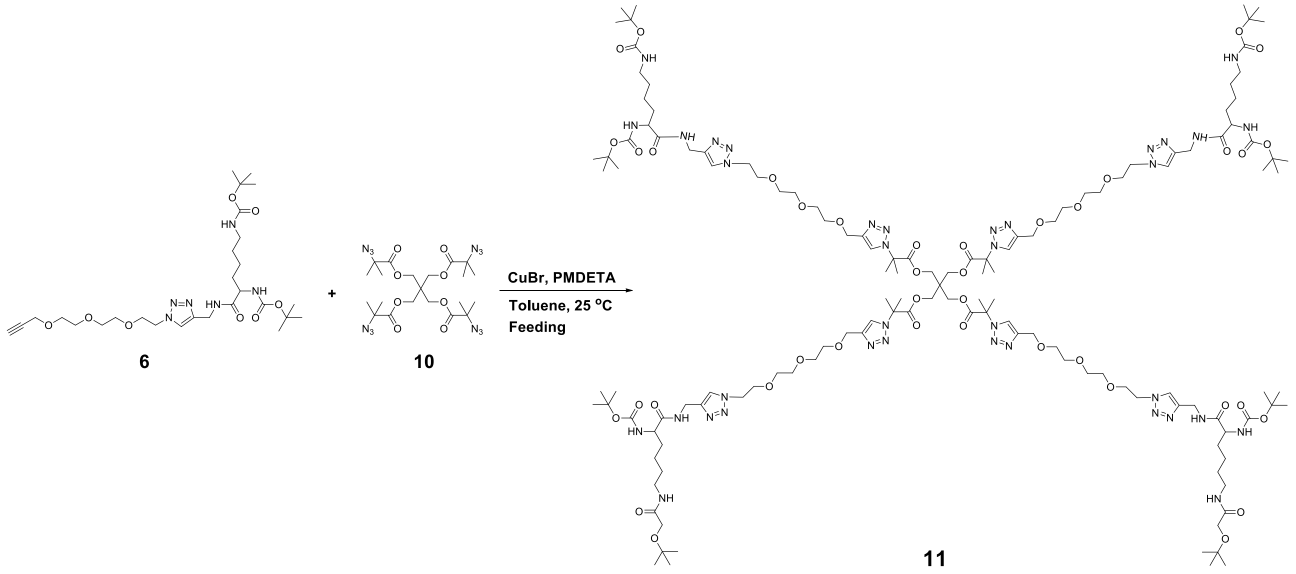

A solution of **6** (0.096 g, 1.613 x 10^{-4} mol) in toluene (5 mL) was purged argon for 30 min to remove oxygen. Meanwhile, a mixture of **10** (21.9 mg, 3.786 x 10^{-5} mol), PMDETA (6.56 mg, 3.786 x 10^{-5} mol) and toluene (5 mL) was degassed by argon for 30 min, to the deoxygenated mixture above, CuBr (5.24 mg, 3.786 x 10^{-5} mol) was added under positive argon flow. Then, the solution of **6** was added via syringe pump, at a flow rate of 0.025 mL min^{-1}, and after feeding the reaction was allowed to react for 2 h. The copper salts were removed by passage through activated basic alumina. The solvent was removed under reduced pressure. The crude product was purified using a Varian Pro-Star preparative SEC system equipped with a manual injector, differential refractive index detector, and single wave-length ultra-violet visible detector. Flow rate was maintained 10 mL min^{-1} and HPLC grade THF was used as the eluent. Separations were achieved using a PL gel 10 mm 1x103 Å, 300 mm x 25 mm preparative SEC column held at 25 °C. After injection, the expected fraction was collected manually with the yield as 18.7 mg. (yielding%=15.9 %).

SEC: (M_n=2220, M_p=2370, PDI=1.025)

MALDI-ToF MS: [**11**+Na^+]=2986.84 (Cal.=2986.67)

Synthesis of 4-arm star-like EG-Lysine conjugate (12)

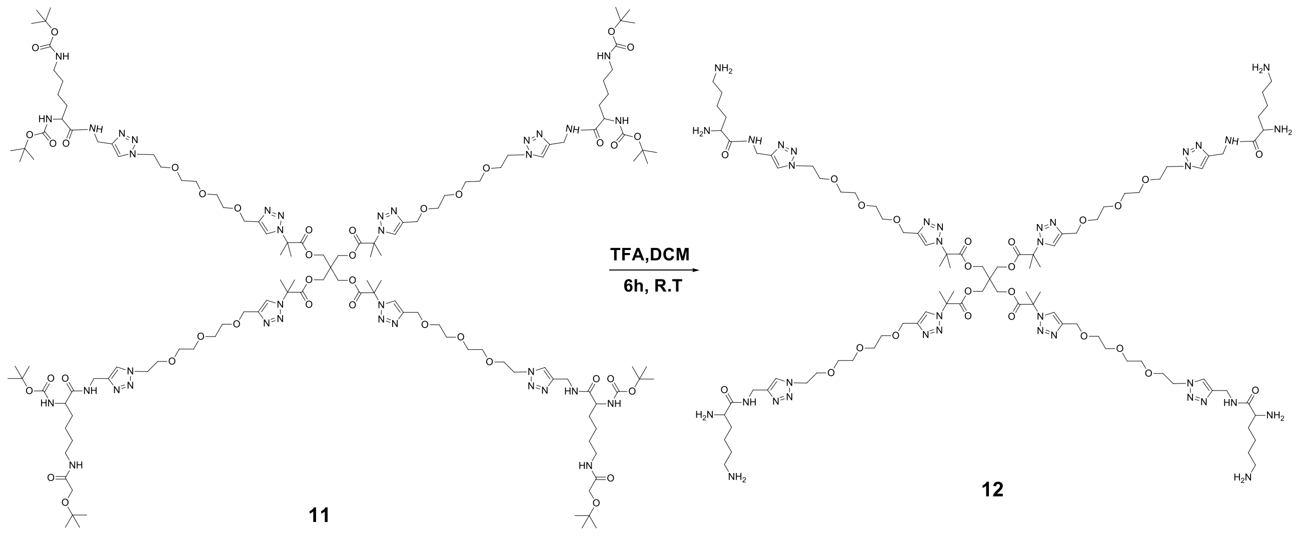

11 (0.015 g) was dissolved in 3 mL mixture of TFA and DCM (1:1, v:v). And the solution was kept stirring for 6 h at room temperature The solvent was remove by reduced pressure, and dried in high vacuum for 24 h, grey yellow oil **12** was produced with near quantitative conversion.

Scheme C. Synthesis of EG-Lysine dendrimer **16**

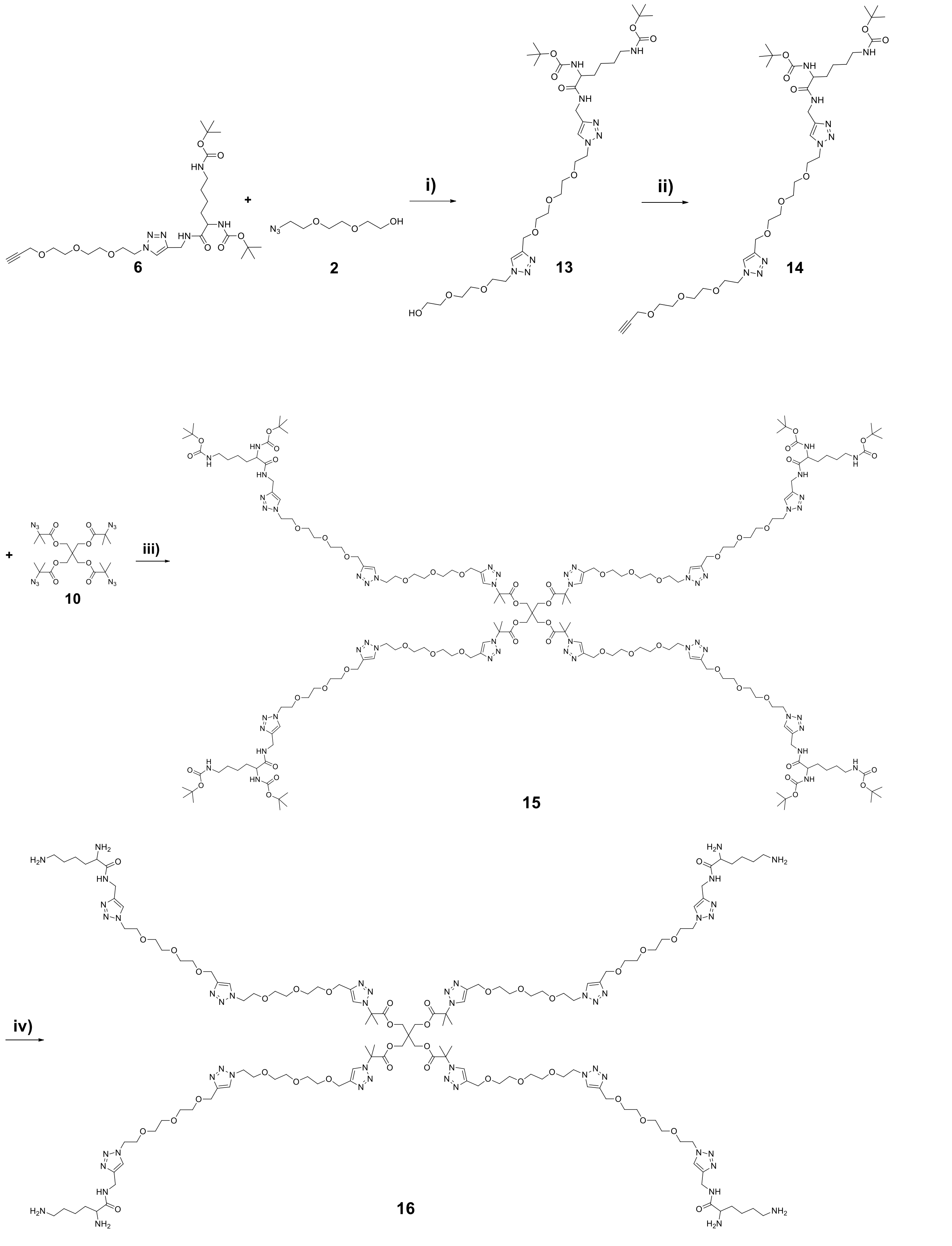

(i) CuBr, PMDETA, Toluene, DMSO, 25 °C, 30 min; (ii) THF, NaH, propargyl bromide, -78°C, 16 h; (iii) CuBr, PMDETA, Toluene,DMSO 25 °C, 120 min; (iv) TFA, DCM, R.T., 6h.

Synthesis of OH-EG-Lysine-Boc conjugate (13)

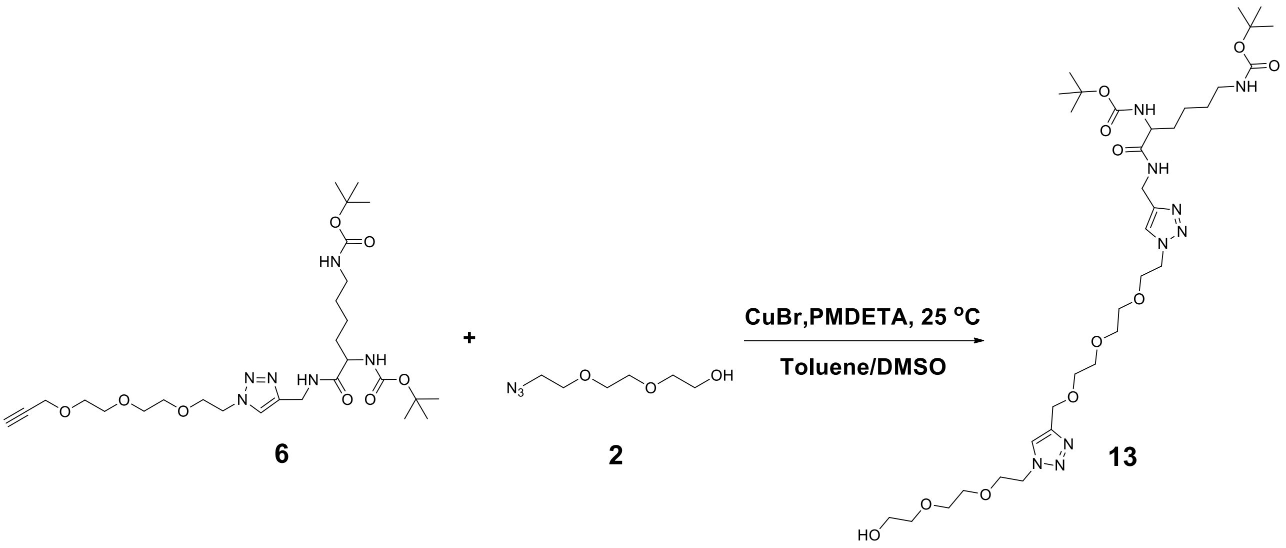

2 (0.095 g, 5.43 x 10^{-4} mol) and **6** (0.21 g, 3.62 x 10^{-4} mol) were placed in a Schlenk tube and dissolved in a mixture of PMDETA (0.0156 g, 9.05 x 10^{-5} mol), toluene (6 mL). Oxygen was removed from the solution by purging with argon for 30 min. Cu(I)Br (0.013 g, 9.05 x 10^{-5} mol) was added under a positive argon flow. The reaction vessel was then sealed and placed in an oil bath at 25 °C and kept stirring for 60min. The mixture was diluted in DCM and passed through activated basic alumina. The solvent was removed under reduced pressure, and the residue was purified by column chromatography with EtOAc-MeOH (4:1, v/v, R_f=0.23) as eluent. 0.16 g viscous oil **13** was obtained with the yield as 59 %.

^{1}H NMR ($CDCl_3$, 298K, 500 MHz): δ 7.79 (s, 1H, -NC***H***CCH$_2$O-, triazole ring proton), 7.68 (s, 1H, -NC***H***C-, triazole ring proton), 7.32 (bd, 1H, -CCH$_2$N***H***CH-), 5.38 (bd, 1H, -CHN***H***C(O)O-), 4.78 (bd, 1H, -CH$_2$N***H***C(O)O-), 4.61 (s, 2H, -CC***H***$_2$CH$_2$O-), 4.49-4.44 (m, 6H, -OCH$_2$C***H***$_2$N-, -CC***H***$_2$NH-), 4.05 (s, 1H, -C(O)C***H***(CH$_2$)NH-), 3.81 (m, 4H, -CH$_2$OC***H***$_2$CH$_2$N-), 3.82-3.54 (m, 16H, -C*H*$_2$- units on EG backbone), 3.02 (s, 2H, -CH$_2$C***H***$_2$NH-), 2.0-1.2 (m, 24H, C***H***$_2$-Lys and C***H***$_3$-Boc). ^{13}C NMR ($CDCl_3$, 298K, 125 MHz): 22.70, 28.54, 29.65, 32.43, 34.99, 40.09, 50.24, 50.33, 54.50, 61.59, 64.55, 69.39, 69.45, 69.69, 70.24, 70.47, 70.49, 70.53, 70.61,79.13, 79.92, 85.07, 123.48, 124.26, 144.61, 144.79, 155.83, 156.29, 171.48, 172.26.

Synthesis of alk-EG-Lysine-Boc conjugate (14)

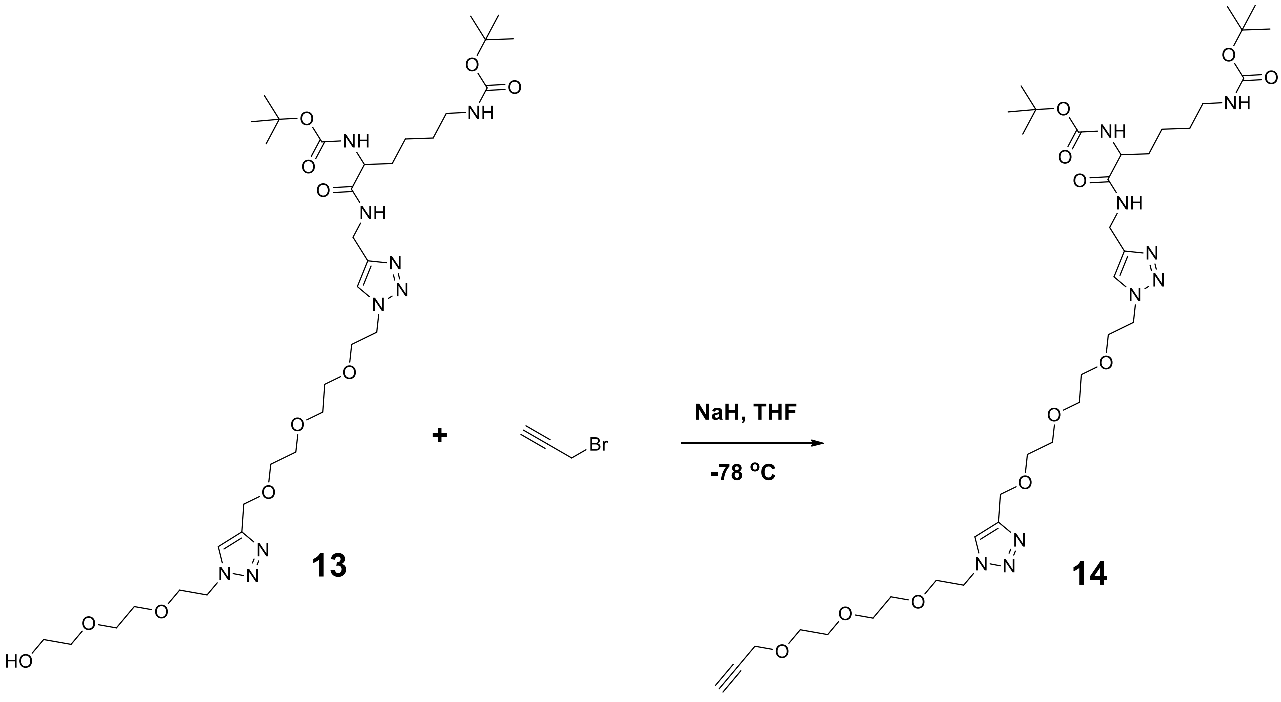

13 (0.11 g, 1.456 x 10^{-4} mol) was dissolved in 4 mL dry THF in a Schlenk tube, the tube was connected to the argon line and the solution was cooled to 0°C in an ice-bath. NaH (0.0059 g, 1.456 x 10^{-4} mol) (60 % in mineral oil) was added in the above solution. The reaction was allowed stirring for 30 min and there was no bubbling if stop the argon. The reaction vessel was then cooled down to -78 ^{o}C in dry ice/acetone mixture. Propargyl bromide (0.026 g, 2.18 x 10^{-4} mol) (80 wt % in toluene) was added to the solution dropwise in 5 min. The reaction was then kept stirring overnight and warmed to RT. The reaction mixture was filtered to remove the salt and concentrated to remove all the solvent and low b.p impurities at RT. The crude brown liquid product was purified by column chromatography with EtOAc-MeOH (6:1, v/v, R_f=0.50) as eluent. 0.02 g viscous oil **14** was obtained with the yield as 17 %.

^{1}H NMR ($CDCl_3$, 298K, 500 MHz): δ 7.78 (s, 2H, -NC***H***CCH_2O-, triazole ring protons), 7.29 (bd, 1H, -CCH_2N***H***CH-), 5.30 (bd, 1H, -CHN***H***C(O)O-), 4.68 (bd, 1H, -CH_2N***H***C(O)O-), 4.64 (s, 2H, -C$C\boldsymbol{H}_2CH_2$O-), 4.49-4.44 (m, 6H, -O$CH_2C\boldsymbol{H}_2$N-, -C$C\boldsymbol{H}_2$NH-), 4.16 (d, 2H, *J*=2.35 Hz, CH$C\boldsymbol{H}_2$O-), 4.09 (s, 1H, -C(O)C***H***(CH_2)NH-), 3.83 (m, 4H, -CH_2O$C\boldsymbol{H}_2CH_2$N-), 3.67-3.55 (m, 16H, -C*H*$_2$- units on EG backbone), 3.02 (s, 2H, -$CH_2C\boldsymbol{H}_2$NH-), 2.43 (t, 1H, *J*=2.3 Hz, C***H***CCH_2-), 2.0-1.2 (m, 24H, C$\boldsymbol{H}_2$-Lys and C$\boldsymbol{H}_3$-Boc).

Synthesis of 4-arm star-like EG-Lysine-Boc conjugate (15)

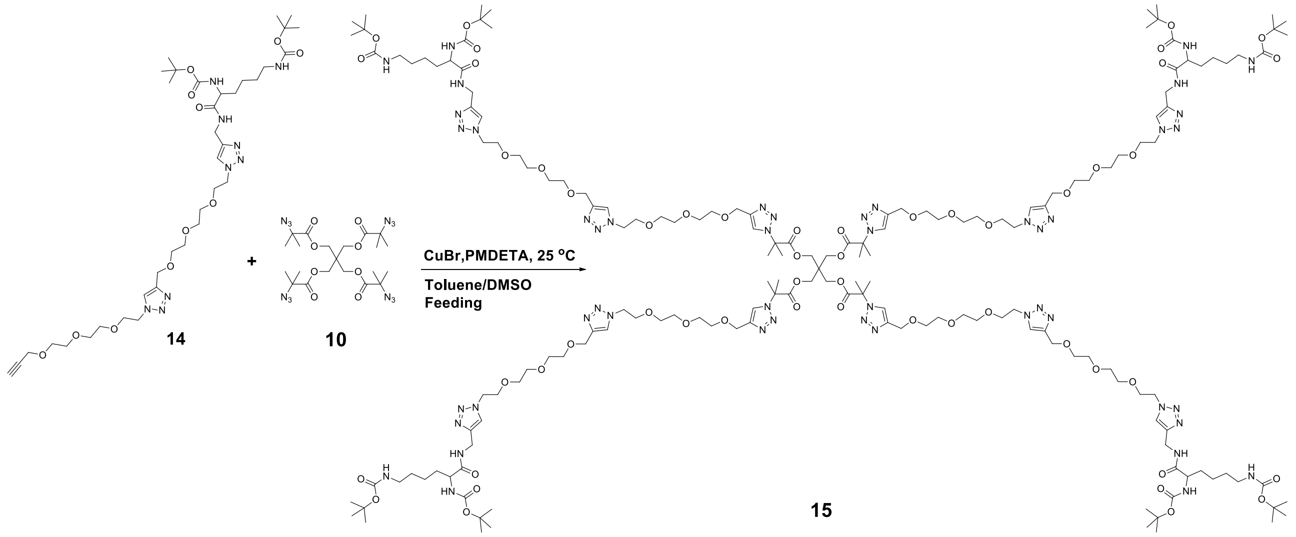

A solution of **14** (19.2 mg, 2.379 x 10^{-5} mol) in toluene (1.6 mL) and DMSO (0.4 mL) was purged with argon for 15 min to remove oxygen. Meanwhile, a mixture of **10** (0.0023 g, 3.966 x 10^{-6} mol), PMDETA (2.74 mg, 1.586 x 10^{-5} mol), toluene (1.6 mL) was degassed by argon for 15 min, to the deoxygenated mixture above, CuBr (2.3 mg, 1.586 x 10^{-5} mol) was added under positive argon flow. Then, the solution of **14** was added via syringe pump, at a flow rate of 0.003 mL min^{-1}, and after feeding the reaction was allowed to react for 2 h. The copper salts were removed by passage through activated basic alumina. The solvent was removed under reduced pressure. The crude product was purified using a Varian Pro-Star preparative SEC system equipped with a manual injector, differential refractive index detector, and single wave-length ultra-violet visible detector. Flow rate was maintained 10 mL min^{-1} and HPLC grade THF was used as the eluent. Separations were achieved using a PL gel 10 mm 1x103 Å, 300 mm x 25 mm preparative SEC column held at 25 ^{o}C. After injection, the expected fraction was collected manually and dried under vacuum gave **15**, with the yield as 4.26 mg (yielding%=23.2%).

SEC: (M_n=2610, M_p=2660, PDI=1.028)

MALDI-ToF MS: [**15**+Na$^+$]=3840.95 (Cal.=3841.09)

Synthesis of 4-arm star-like EG-Lysine conjugate (16).

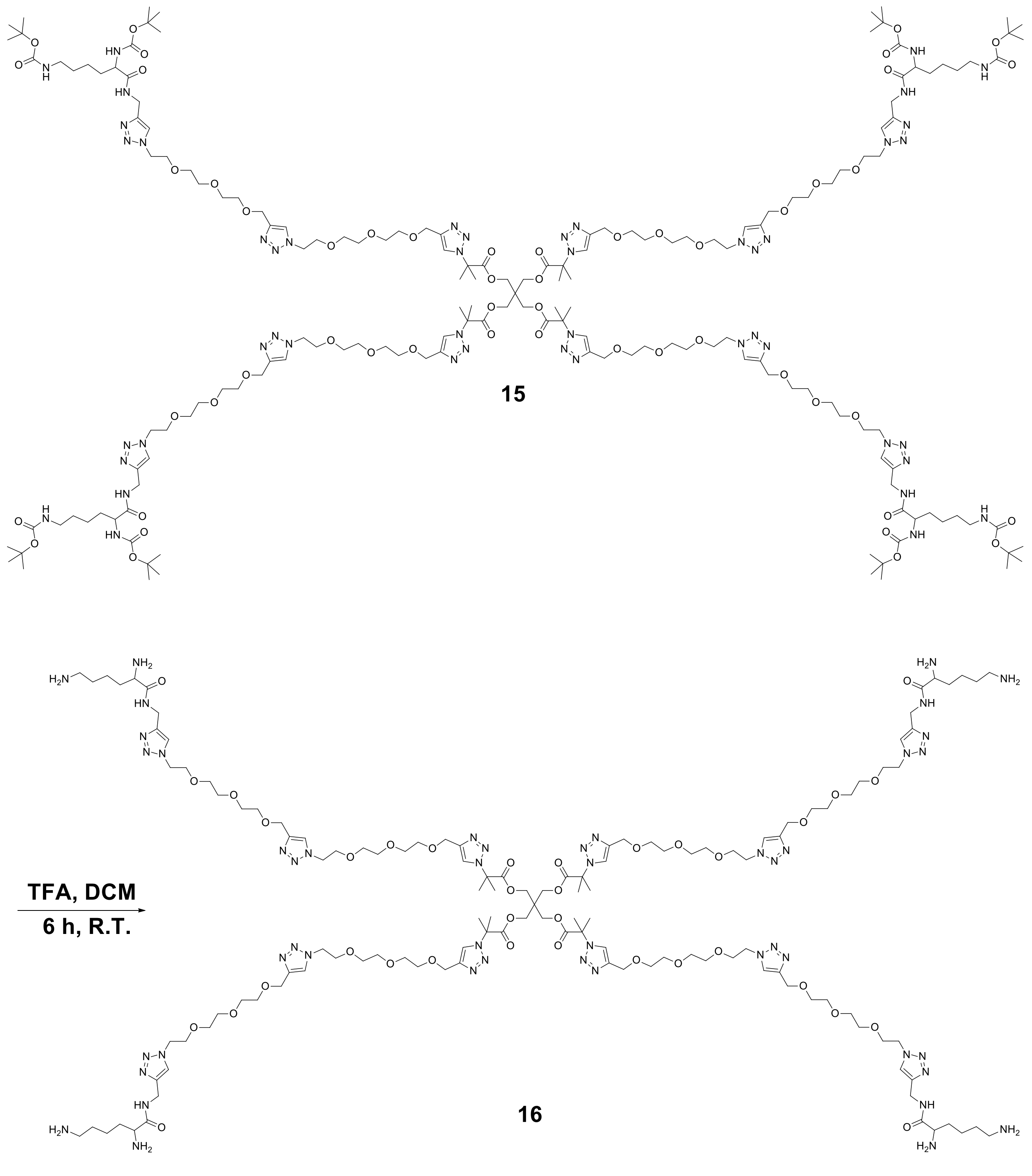

15 (0.015 g) was dissolved in 3 mL mixture of TFA and DCM (1:1, v:v). And the solution was kept stirring for 6 h at room temperature The solvent was remove by reduced pressure, and dried in high vacuum for 24 h, grey yellow oil **16** was produced with near quantitative conversion.

Scheme D. Synthesis of EG-Lysine dendrimer **25**

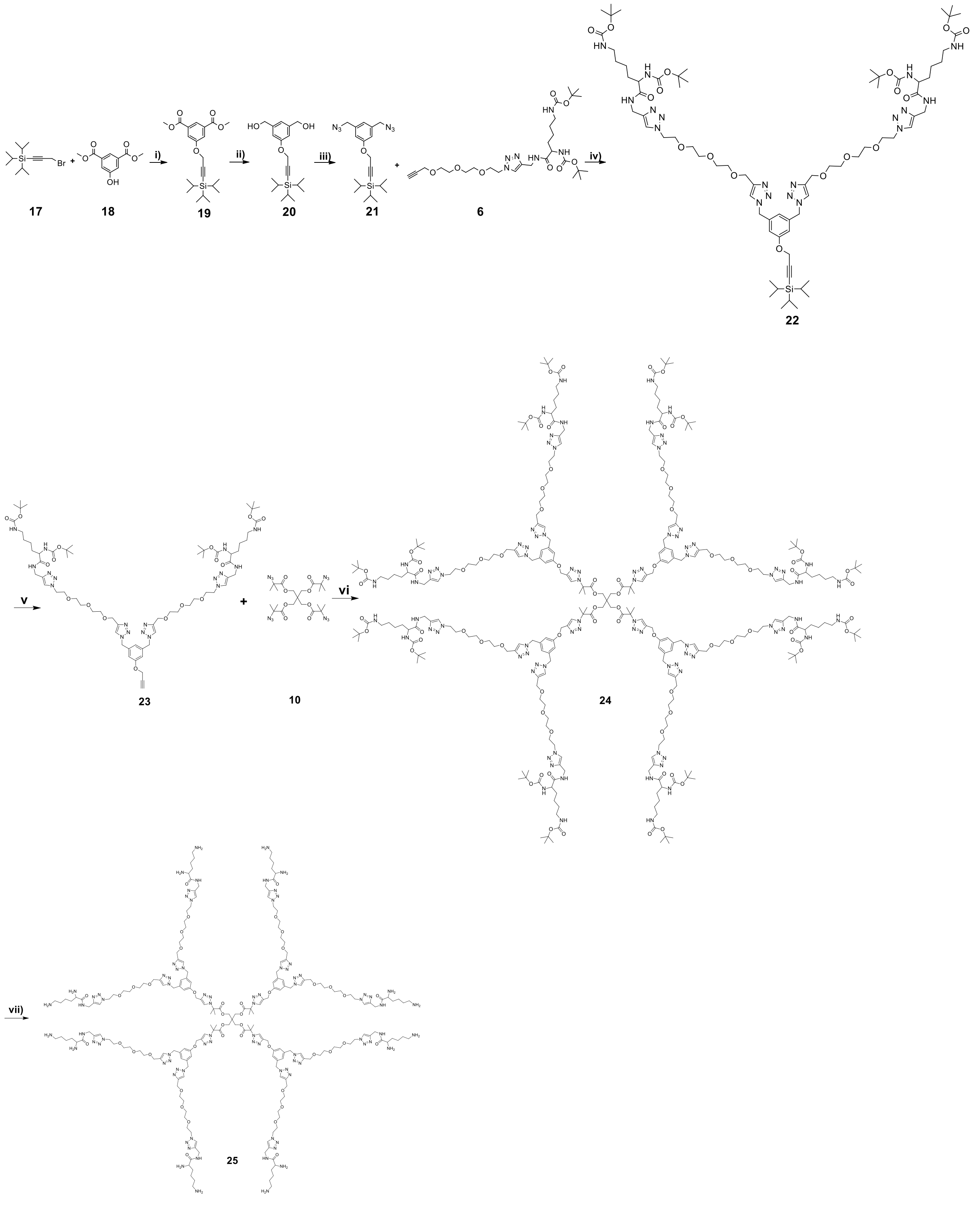

i): 18-C-6, K_2CO_3, Acetone, reflux, 48 h. ii): $LiAlH_4$, THF, 0 ^oC-R.T., 16 h, iii): DPPA, DBU, toluene, 0 ^oC-R.T., dark.(iv) CuBr, PMDETA, Toluene, DMSO, 25 ^oC, 30 min; (v) TBAF, THF, argon, 25 ^oC; (vi) CuBr, PMDETA, Toluene, DMSO 25 ^oC, 120 min; (vii) TFA, DCM, R.T., 6h.

Synthesis of (17)

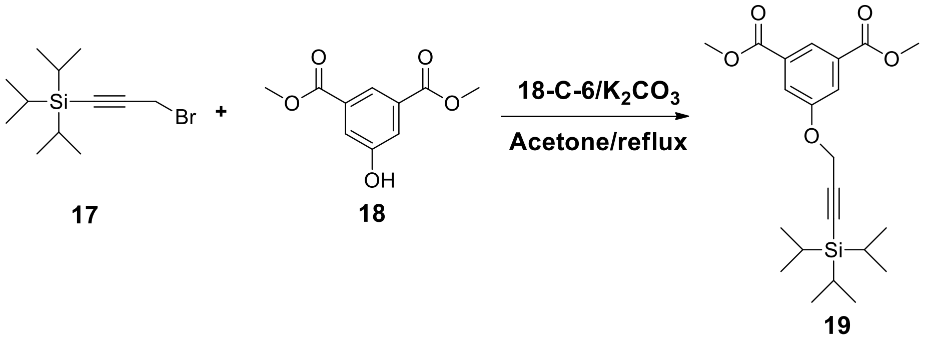

Compound **17** was synthesized according to reference [15]. Dimethyl 5-hydroxyisophthalate (**18**, 3.2 g, 1.53 x 10^{-2} mol), **17** (2.8 g, 1.01 x 10^{-2} mol) and 18-crown-6 ether (0.40 g, 1.53 x 10^{-2} mol) were dissolved in 80 mL of acetone. Anhydrous potassium carbonate (2.1 g, 0.028 mol) was added and the reaction was refluxed at 80 °C for 48 h. The reaction was then cooled to room temperature, filtered, and the filtrate was concentrated by rotary evaporation. The crude product was purified by column chromatography using ethyl acetate/petroleum spirit (1/5, v/v, R_f=0.43) as the eluent. Upon drying the product, **19**, was obtained as white crystals (3.0 g, 62.0 %).

^{1}H NMR ($CDCl_3$, 298K, 500 MHz): δ 8.30 (t, *J*=1.79 Hz, 1H; aromatic proton), 7.83 (d, *J*=1.80 Hz, 2H; aromatic proton), 4.79 (s, 2H; -SiCCC***H***$_2$O-), 3.91 (s, 6H; C***H***$_3$O-), 0.98 (s, 21H; -SiC***H***(CH_3)$_2$- and -SiCH(C***H***$_3$)$_2$-). ^{13}C NMR ($CDCl_3$, 298K, 125 MHz) δ, ppm: 11.10, 18.50, 52.46, 57.16, 90.53, 100.89, 120.77, 123.76, 131.78, 157.63, 166.07.

Synthesis of (20)

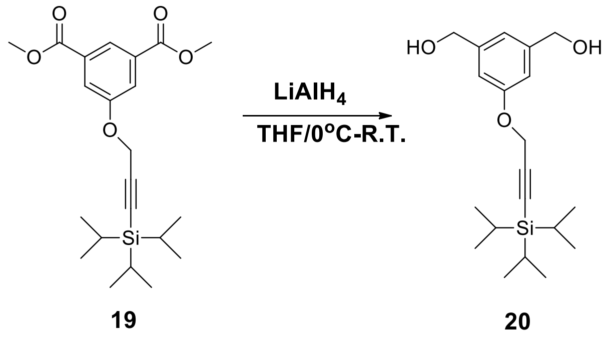

19 (3.0 g, 7.42 x 10^{-3} mol) was dissolved in 60 mL of dry THF at 0 °C under argon. Lithium aluminium hydride (0.65 g, 0.017 mol) was added to the solution portion-wise over 30 min. After stirring for 16 h, the reaction was quenched by slowly adding crushed $Na_2SO_4.10H_2O$. *Caution! Hydrogen is produced.* The mixture was filtered and the filtrate concentrated by rotary evaporation. The product was purified by column chromatography using MeOH/DCM (1/10, v/v, R_f=0.38) as the eluent, and the product, **20**, was obtained as white solid (1.8 g, 70 %).

^{1}H NMR ($CDCl_3$, 298K, 400 MHz): δ 6.92 (s, 1H; aromatic proton), 6.93 (s, 2H; aromatic proton), 4.72 (s, 2H; -SiCCC$\boldsymbol{H}_2$O-), 4.64 (s, 4H; OHC$\boldsymbol{H}_2$C-), 1.01 (s, 21H; -SiC$\boldsymbol{H}$(CH_3)$_2$- and -SiCH(C$\boldsymbol{H}_3$)$_2$-). ^{13}C NMR ($CDCl_3$, 298K, 100 MHz) δ, ppm: 11.05, 18.48, 56.76, 65.04, 89.30, 101.87, 112.86, 118.07, 142.67, 158.10.

Synthesis of (21)

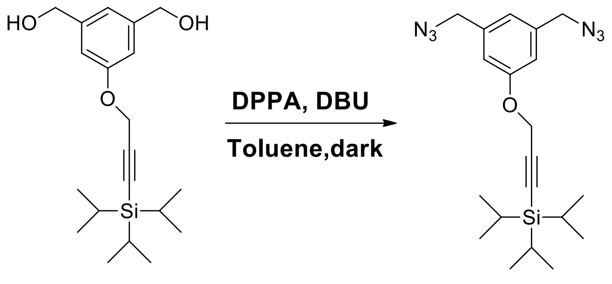

20 (1.8 g, 5.17 x 10^{-3} mol), DPPA (3.55 g, 1.29 x 10^{-2} mol) and DBU (1.96 g, 1.29 x 10^{-2} mol) were added to 35 mL of dry toluene at 0 °C. The flask was wrapped in aluminium foil to avoid light. The solution was stirred for 16 h, and then poured into a separation funnel. The colorless toluene phase was then collected and concentrated by rotary evaporation. The product was purified by column chromatography using ethyl acetate/petroleum spirit (1/6, v/v, R_f=0.48) as the eluent and the product, **21**, was obtained as a colorless oil (1.62 g, 79.0 %).

^{1}H NMR ($CDCl_3$, 298K, 500 MHz): δ 6.92 (s, 2H; aromatic proton), 6.86 (s, 1H; aromatic proton), 4.732 (s, 2H; -SiCCC$\boldsymbol{H}_2$O-), 4.30 (s, 4H; N_3C$\boldsymbol{H}_2$C-), 1.01 (s, 21H; -SiC$\boldsymbol{H}$(CH_3)$_2$- and -SiCH(C$\boldsymbol{H}_3$)$_2$-). ^{13}C NMR ($CDCl_3$, 298K, 125 MHz) δ, ppm: 11.15, 18.58, 54.59, 56.76, 89.94, 101.53, 114.85, 120.50, 137.55, 158.47.

Synthesis of alk-EG-(Lysine-Boc)$_2$ conjugate (23)

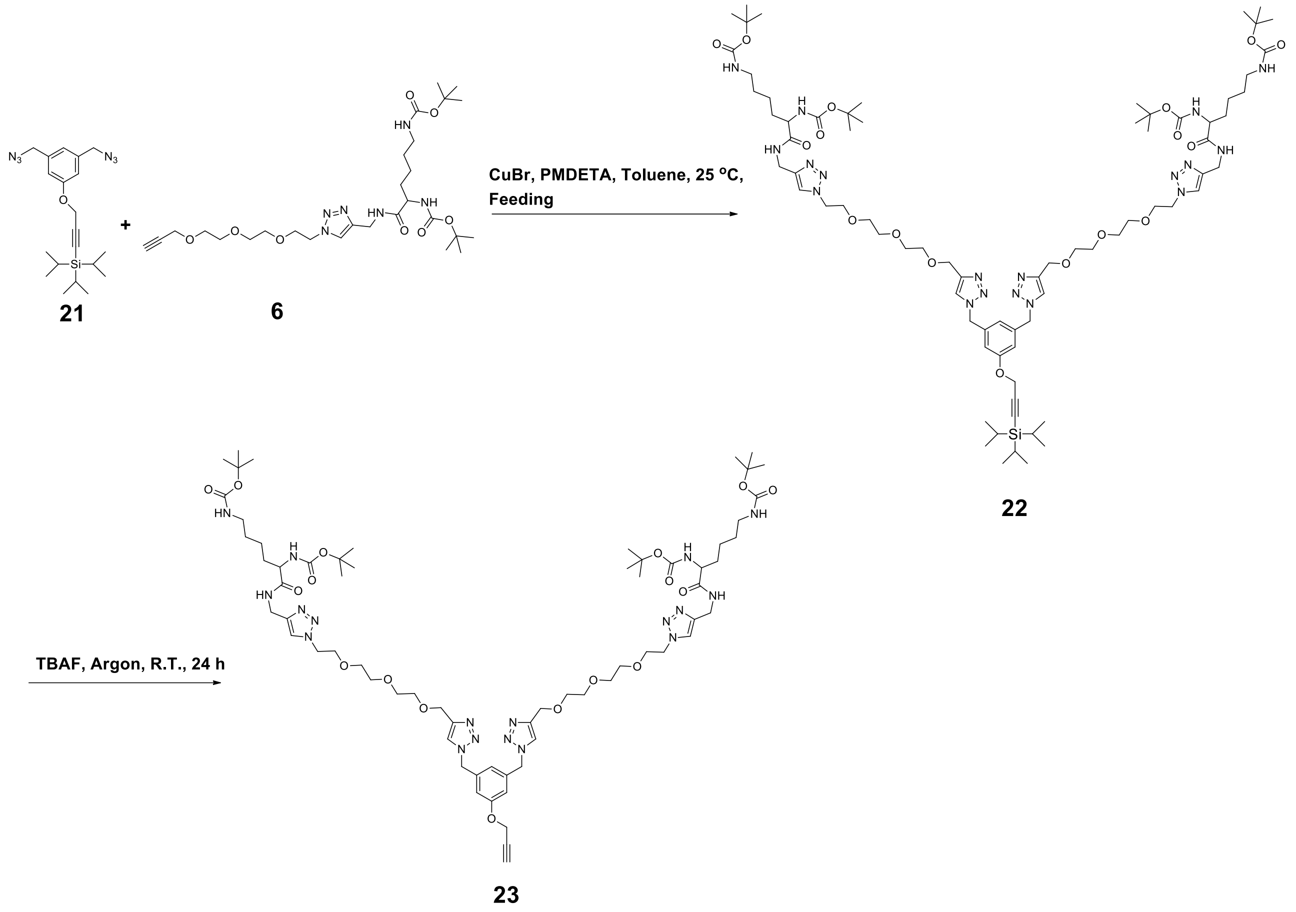

A mixture of **21** (12.2 mg, 3.065 x 10^{-5} mol), PMDETA (5.30 mg, 3.065 x 10^{-5} mol), and toluene (1 mL) was purged with argon for 15 min to remove oxygen. To the deoxygenated mixture above, CuBr (4.51 mg, 3.065 x 10^{-5} mol) was added under positive argon flow. Meanwhile, a solution of **6** (0.046 g, 7.718 x 10^{-5} mol), toluene (2 mL) was degassed by argon for 15 min. The degassed solution of **6** was added via syringe pump, at a flow rate of 0.004 mL min^{-1}, and after feeding the reaction was allowed to react for 2 h. The copper salts were removed by passage through activated basic alumina. The residue was concentrated by rotary evaporation and dried in high vacuum for 24 h gave **22**. Crude product of **22** was directly added in a mixture of TBAF (0.125 mL, 1M in THF) and 0.2 mL THF to remove the Tips protecting group. The reaction was kept stirring for 24 h under protection of argon. The product was concentrated by rotary evaporation and purified by column chromatography using DCM/MeOH/petroleum spirit (10/6/2, v/v/v, R_f=0.24) as the eluent and the product, **23**, was obtained as a colorless oil (17.6 mg, 49.0 %).

^{1}H NMR ($CDCl_3$, 298K, 500 MHz): δ 7.72 (s, 1H, -CH_2NC***H***C-, triazole ring proton), 7.59 (s, 1H, -NC***H***C-, triazole ring proton) 7.30 (bd, 1H, -CCH_2N***H***CO-), 6.85 (s, 1H; aromatic proton), 6.80 (s, 2H; aromatic proton), 5.46 (s, 2H, -C***H***$_2$N-), 5.33 (bd, 1H, -CHN***H***C(O)O-), 4.73 (bd, 1H, -

CH_2N***H***C(O)O-), 4.59-4.57 (m, 4H, CHC***H***$_2$NO-, -CC***H***$_2$O-) 4.60-4.45 (m, 4H, -CH_2C***H***$_2$N-, -CC***H***$_2$NH-), 4.08 (s, 1H, -C(O)C***H***(CH_2)NH-), 3.81 (t, 2H, *J*=4.90 Hz, -COC***H***$_2$$CH_2$N-), 3.81-3.44 (m, 8H, -C*H*$_2$- units on EG backbone), 3.04 (t, 2H, *J*=6.6 Hz, -CH_2C***H***$_2$NH-), 2.57 (t, 1H, *J*=2.3 Hz, C***H***CCH_2-), 1.80-1.37 (m, 24H, C***H***$_2$-Lys and C***H***$_3$-Boc). ^{13}C NMR ($CDCl_3$, 298K, 125 MHz): 22.69, 28.41, 29.65, 29.76, 32.38, 34.96, 40.07, 50.32, 53.65, 54.52, 56.03, 64.53, 69.42, 69.84, 70.47, 70.58, 76.80, 77.98, 79.16, 79.95, 114.87, 120.63, 123.14, 123.50, 137.33, .144.53, 145.46, 155.86, 156.32, 158.45, 172.54.

Synthesis of 4-arm star-like EG-(Lysine-Boc)$_2$ conjugate (24)

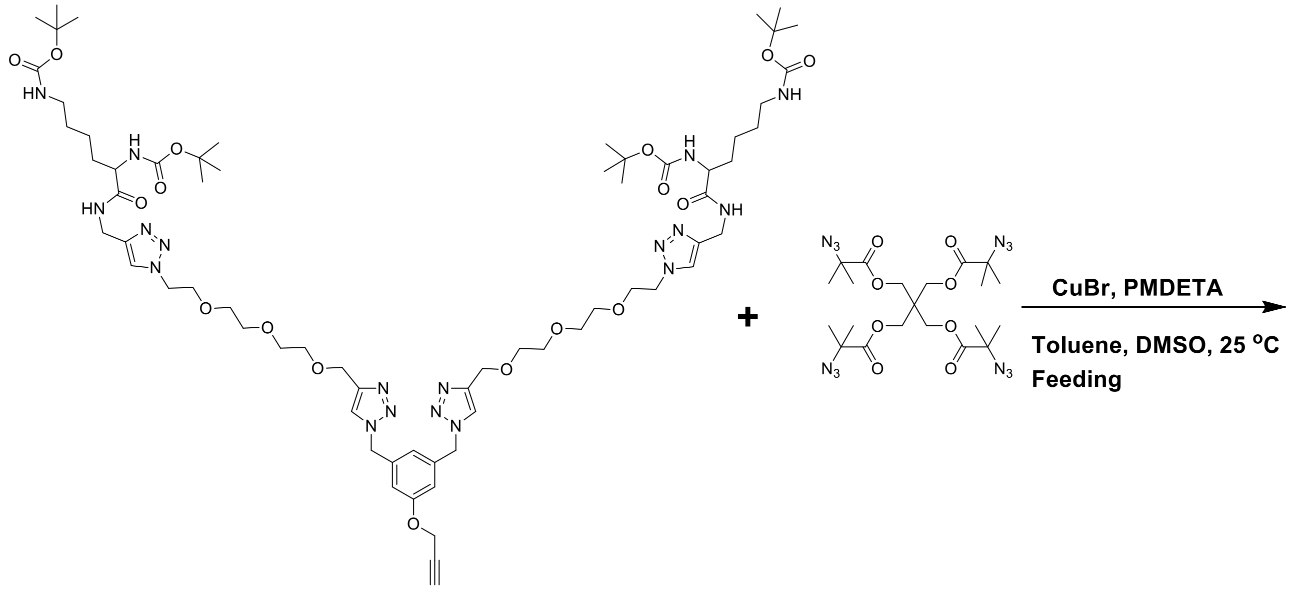

23 **10**

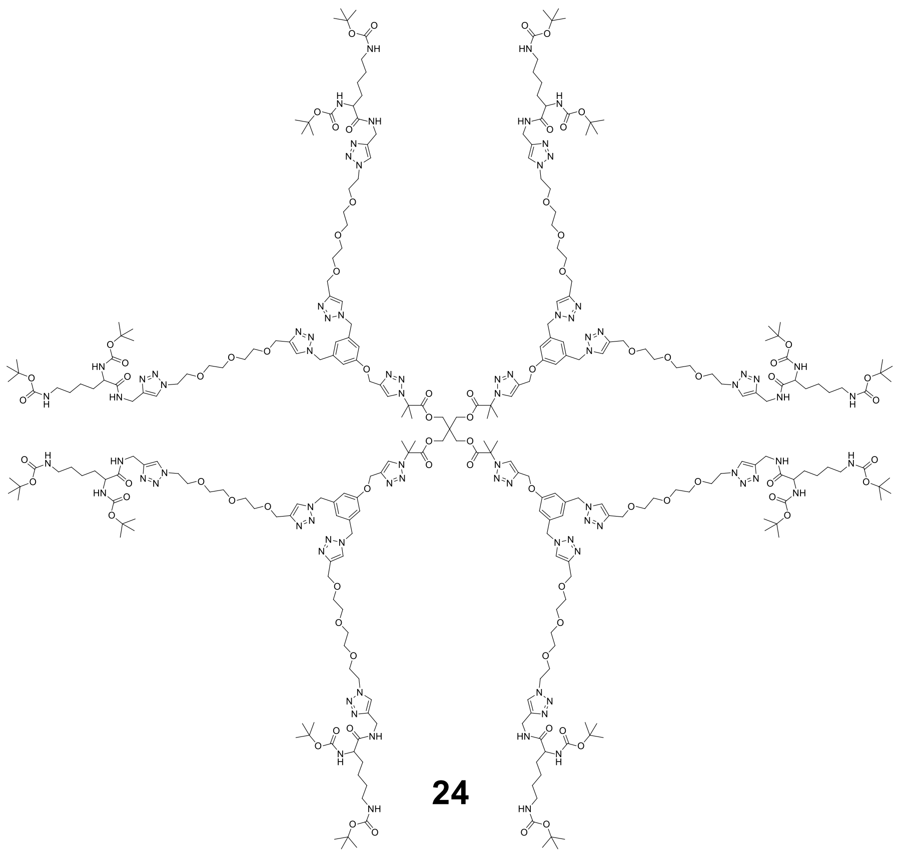

24

A solution of **23** (13.6 mg, 9.482 x 10^{-6} mol) in toluene (2 mL) and DMSO (0.5 mL) was purged with argon for 15 min to remove oxygen. Meanwhile, a mixture of **10** (1.1 mg, 1.896 x 10^{-6} mol), PMDETA (1.64 mg, 9.482 x 10^{-6} mol), toluene (1.0 mL) was degassed by argon for 15 min, to the

deoxygenated mixture above, CuBr (1.36 mg, 9.482 x 10^{-6} mol) was added under positive argon flow. The degassed solution of **24** was added via syringe pump, at a flow rate of 0.008 mL min^{-1}, and after feeding the reaction was allowed to react for 2 h. The copper salts were removed by passage through activated basic alumina. The solvent was removed under reduced pressure. The crude product was purified using a Varian Pro-Star preparative SEC system equipped with a manual injector, differential refractive index detector, and single wave-length ultra-violet visible detector. Flow rate was maintained 10 mL min^{-1} and HPLC grade THF was used as the eluent. Separations were achieved using a PL gel 10 mm 1x103 Å, 300 mm x 25 mm preparative SEC column held at 25 ^{o}C. After injection, the expected fraction was collected manually with the yield as 4.32 mg, (yielding%=29.9 %).

SEC: (M_n=3870, M_p=3930, PDI=1.025)

MALDI-ToF MS: [**24**+Na^+]=6344.96 (Cal.=6345.76)

Synthesis of 4-arm star-like EG-(Lysine)$_2$ conjugate 25.

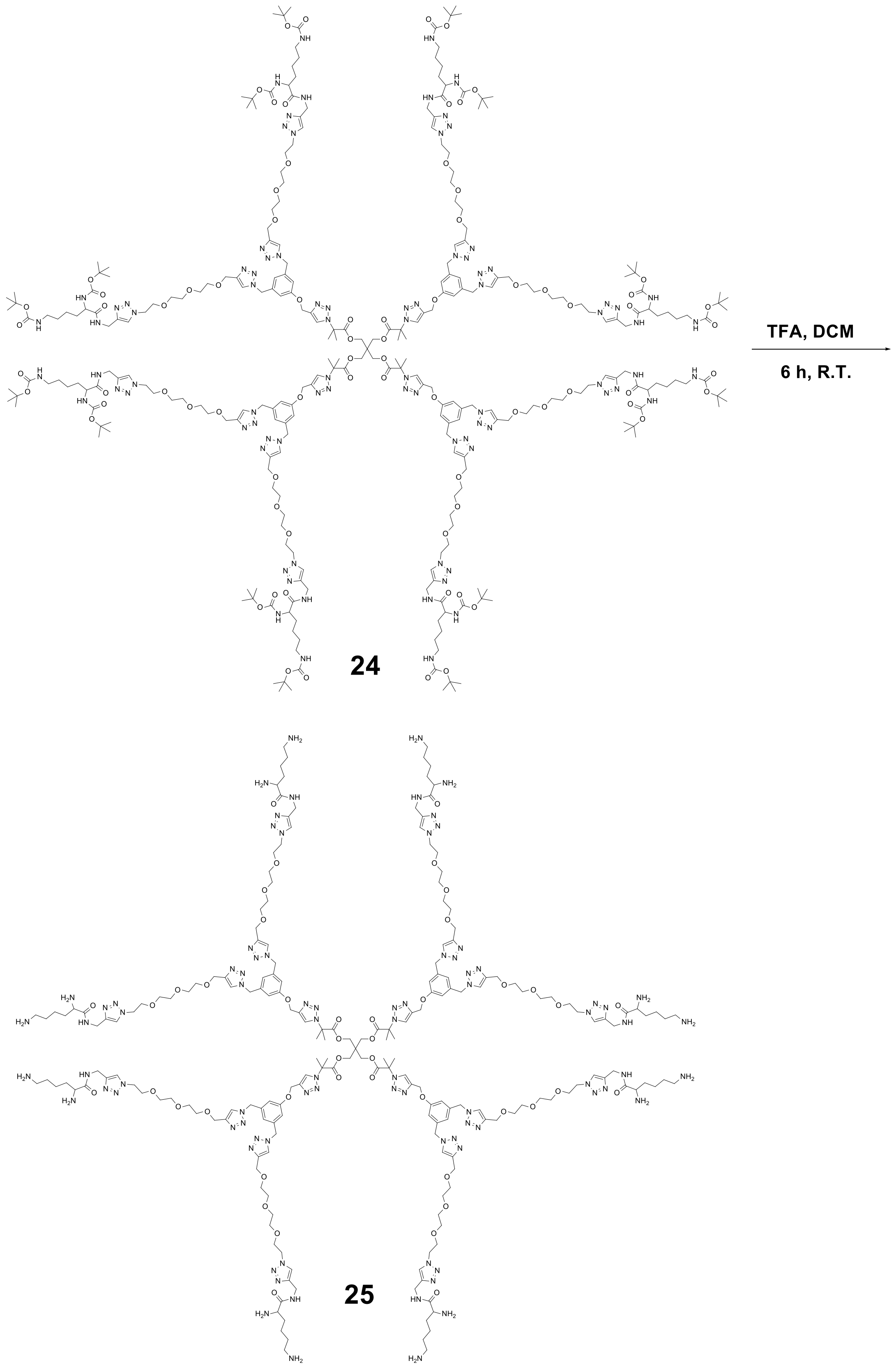

24 (0.0056 g) was dissolved in 2 mL mixture of TFA and DCM (1:1, v:v). And the solution was kept stirring for 6 h at room temperature The solvent was remove by reduced pressure, and dried in high vacuum for 24 h, grey yellow oil **25** was produced with near quantitative conversion.

Scheme E. Synthesis of EG-MKF dendrimer **31**

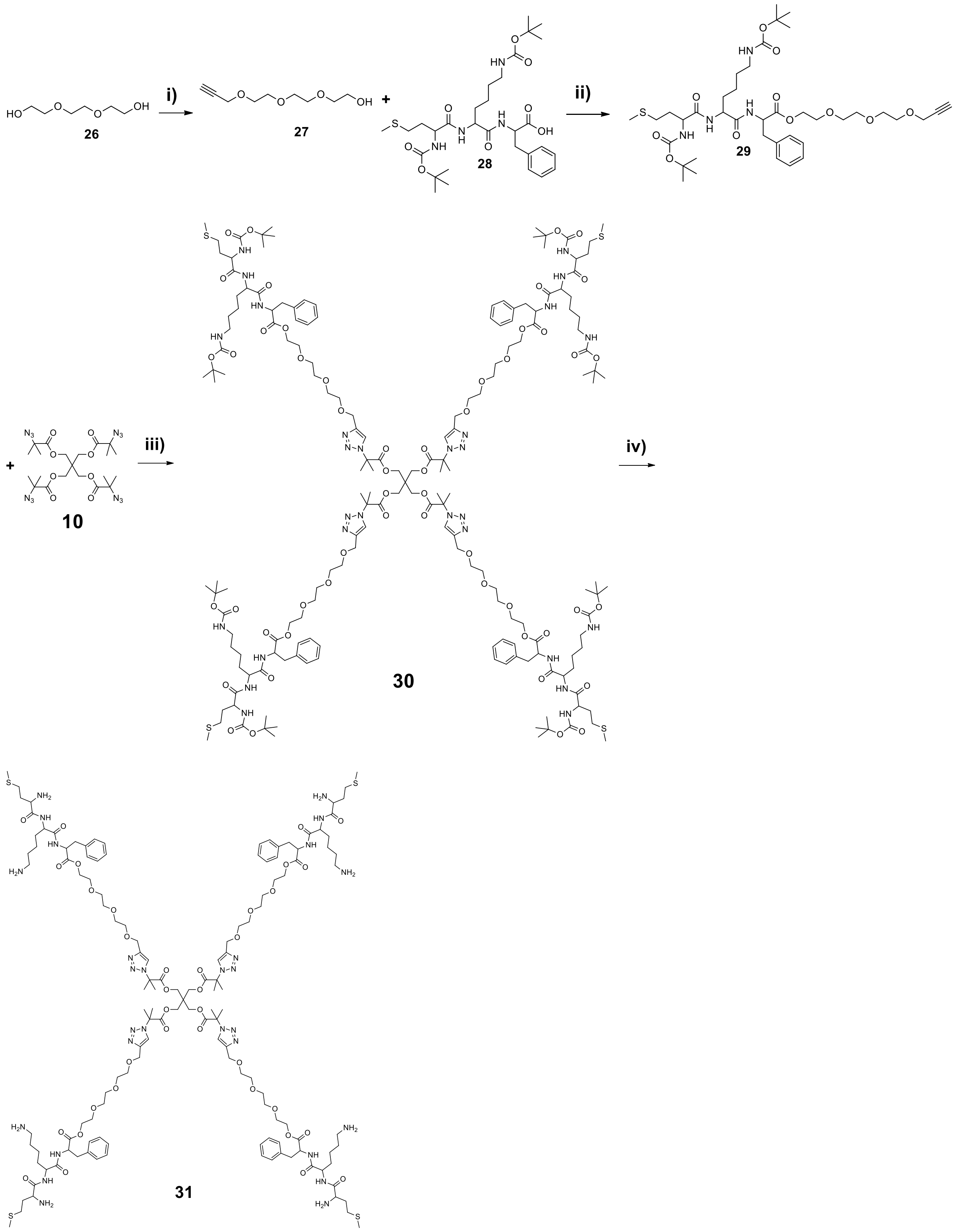

i) THF, NaH, propargyl bromide, -78 ^{o}C, 16 h; ii) DCC, DMAP, DCM, $0^{o}C$-R.T.; iii) CuBr, PMDETA, Toluene, 25 ^{o}C, 120 min; iv) TFA, DCM, R.T., 6h.

Synthesis of 2-(2-(2-(prop-2-yn-1-yloxy)ethoxy)ethoxy)ethanol (27)

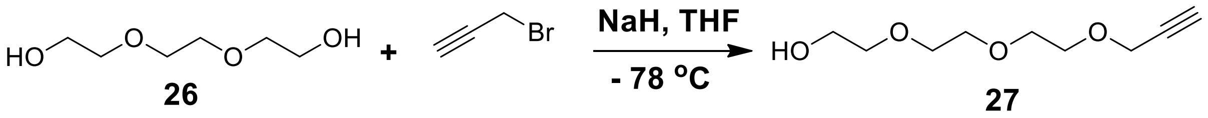

Triethylene glycol (**26**, 15.0 g, 0.1 mol) was dissolved in 10 mL dry THF in a 25 mL two-neck round bottom flask, the flask was connected to the argon line and the solution was cooled to 0°C in an ice-bath. NaH (3 g, 7.50 x 10^{-2} mol) (60 % in mineral oil) was added proportionally in the above solution in 30 min. The reaction was allowed stirring for 1 h and there was no bubbling if stop the argon. The reaction vessel was then cooled down to -78 °C in dry ice/acetone mixture. Propargyl bromide (9.83 g, 6.67 x 10^{-2} mol) (80 wt % in toluene) was added to the solution dropwise in 1 h. The reaction was then kept stirring overnight and warmed to RT. The reaction mixture was filtered to remove the salt and concentrated to remove all the solvent and low b.p impurities at RT. The crude brown liquid product was purified by column chromatography with DCM-MeOH (4:1, v/v, R_f=0.49) as eluent. 6.7 g viscous oil **27** was obtained with the yield as 43 %.

^{1}H NMR ($CDCl_3$, 298K, 500 MHz): δ 4.17 (d, 2H, *J*=2.35 Hz, CHCC***H***$_2$O-), 3.71-3.63 (m, 12H, -C***H***$_2$-), 3.58 (t, 2H, *J*=4.55 Hz, -C***H***$_2$CH$_2$OH), 2.44 (bd, 1H, O***H***CH$_2$-), 2.40 (bd, 1H, *J*=4.55 Hz, -OCH$_2$CC***H***); ^{13}C NMR ($CDCl_3$, 298K, 125 MHz): 58.51, 61.85, 69.17, 70.45, 70.48, 72.73, 72.56, 74.70, 79.67.

NMR analysis of OH-Met-Lys-Phe-Boc (28)

Peptide **28** was purchased from Auspep Pty Ltd and fully characterized by NMR. The data shown as following:

^{1}H NMR ($CDCl_3$, 298K, 500 MHz): 7.30 (bd, 1H, -CH(NH)C(O)N***H***CH(CH$_2$)-), δ 7.28-7.20 (m, 5H, aromatic proton), 6.90-7.03 (dd, 1H, *J*=7.65 Hz, *J*=7.35 Hz -C(O)N***H***CH(CH$_2$)-), 5.52 (bd, 1H, -CHN***H***C(O)O-), 4.91 (bd, 1H, -CH$_2$N***H***C(O)O-), 4.75 (m, 1H, -NHC***H***C(O)CH$_2$-), 4.41 (m, 1H, -NHC***H***C(O)CH$_2$-), 4.24 (m, SCH$_2$CH$_2$C***H***C(O)NH-), 3.19-2.98 (m, 4H, -OC(O)NHC***H***$_2$CH$_2$-, -CHC***H***$_2$C-), 2.45 (m, -SC***H***$_2$CH$_2$-), 2.02 (d, *J*=2.35 Hz, C***H***$_3$S-) 1.98-1.82 (mm, -SCH$_2$C***H***$_2$CH-), 1.70-1.20 (m, 24H, C***H***$_2$-Lys and C***H***$_3$-Boc); ^{13}C NMR ($CDCl_3$, 298K, 125 MHz): 15.44, 22.38, 28.44, 28.55, 30.23, 31.95, 32.18, 33.43,37.36, 40.13, 52.79, 53.09, 53.37, 79.53, 80.38, 127.14, 128.67, 129.50, 136.24, 155.83, 157.74, 171.70, 172.05, 173.76.

Synthesis of alk-EG-Met-Lys-Phe-Boc (29)

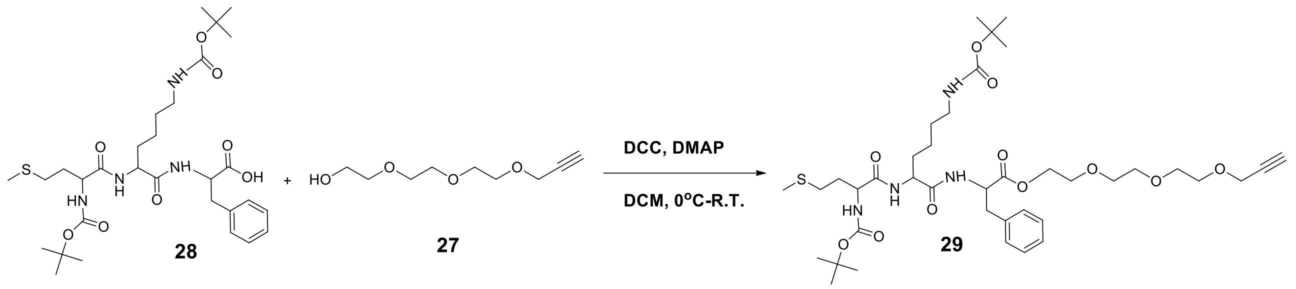

To a 100 mL flask, Boc-Met-Lys(Boc)-Phe-OH (Boc-MKF-OH) **28** (0.5 g, 8.01 x 10^{-4} mol), **27** (0.55 g, 2.80 x 10^{-3} mol), and DMAP (0.015 g, 2.59 x 10^{-3} mol) were dissolved in 20 mL dry DCM and cooled to 0 °C in an ice-bath. A mixture of DCC (0.32 g, 1.58 x 10^{-3} mol) and 10 mL DCM was added dropwise into the solution over 30 min. The mixture was allowed to react for 48 h at room temperature. The solid content was removed by filtration and the filtrate was washed by saturated brine (2 x 50 mL). The organic layer was collected, dried over anhydrous $MgSO_4$, the solvent removed *in vacuo* followed by column chromatography using ethyl acetate/petroleum spirit (5/1, v/v, R_f=0.48) as the eluent. Product **29** was obtained as a white powder (0.31 g, yield%=46.0 %).

^{1}H NMR ($CDCl_3$, 298K, 500 MHz): δ 7.28-7.20 (m, 5H, aromatic proton), 6.74 (bd, 1H, -CH(NH)C(O)N***H***CH(CH$_2$)-), 6.67-6.53 (dd, 1H, *J*=7.65 Hz, *J*=7.35 Hz -C(O)N***H***CH(CH$_2$)-), 5.25 (bd, 1H, -CHN***H***C(O)O-), 4.82 (m, 1H, -NHC***H***C(O)CH$_2$-), 4.71 (bd, 1H, -CH$_2$N***H***C(O)O-), 4.34 (m, 1H, -NHC***H***C(O)CH$_2$-), 4.30-4.16 (m, 5H, -SCH$_2$CH$_2$C***H***C(O)NH-, -OC***H***$_2$CCH, -C(O)OC***H***$_2$CH$_2$-), 3.66-3.62 (m, 10H, -C*H*$_2$- units on EG backbone), 3.19-3.01 (m, 4H, -OC(O)NHC***H***$_2$CH$_2$-, -CHC***H***$_2$C-), 2.50 (m, -SC***H***$_2$CH$_2$-), 2.42 (t, -CH$_2$C***H***), 2.06 (d, *J*=2.35 Hz, C***H***$_3$S-) 2.03-1.88 (mm, -SCH$_2$C***H***$_2$CH-), 2.0-1.2 (m, 24H, C***H***$_2$-Lys and C***H***$_3$-Boc); ^{13}C NMR ($CDCl_3$, 298K, 125 MHz): 15.40, 22.41, 28.42, 28.56, 31.75, 31.93, 34.30, 37.80, 40.13, 52.79, 52.98, 53.37, 58.47, 64.59, 68.87, 69.17, 70.50, 70.65, 70.68, 74.78, 79.21, 79.67, 80.40, 127.25, 128.72, 129.46, 135.24, 155.75, 156.14, 170.94, 171.27, 171.32.

Synthesis of 4-arm star-like EG-MKF-Boc conjugate (30)

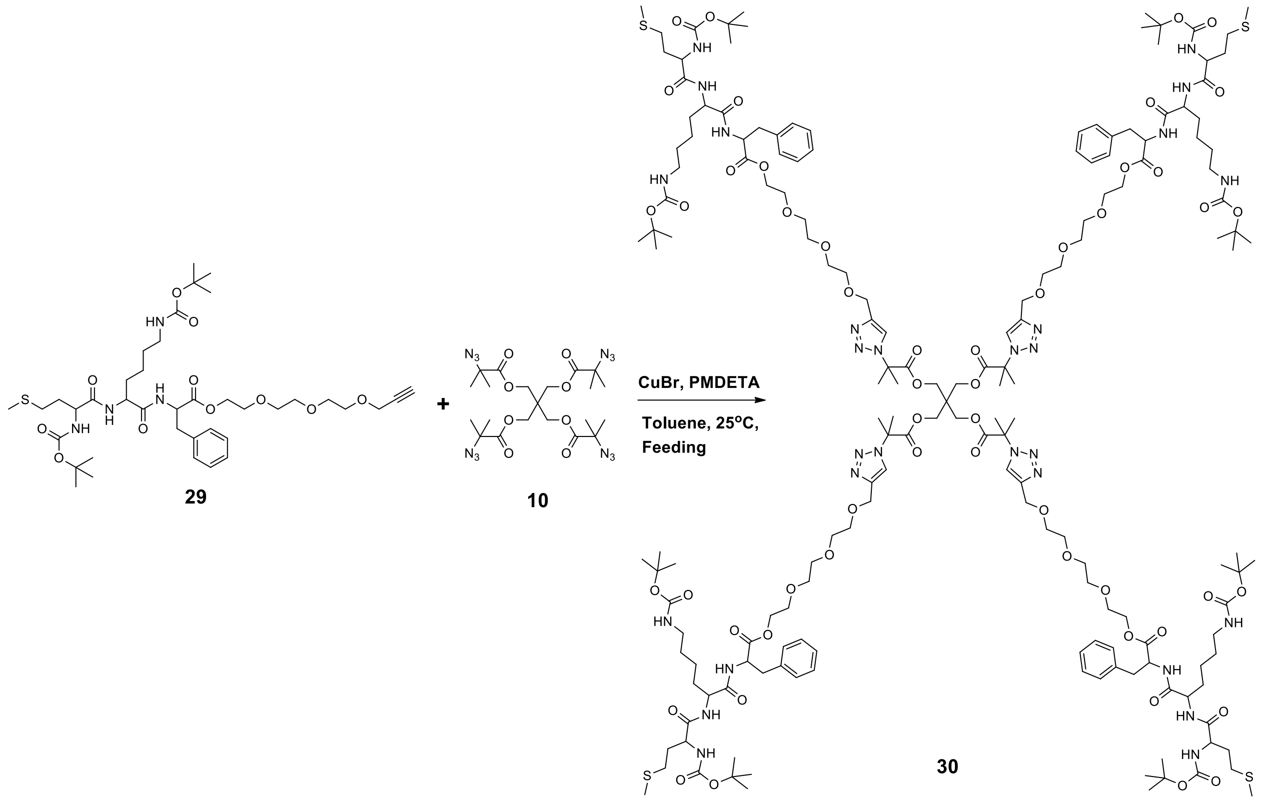

A solution of **29** (0.184 g, 2.327 x 10^{-4} mol) in toluene (20 mL) was purged with argon for 60 min to remove oxygen. Meanwhile, a mixture of **10** (0.027 g, 4.655 x 10^{-5} mol), PMDETA (5.3 mg, 3.007 x 10^{-5} mol), toluene (20 mL) was degassed by argon for 60 min, to the deoxygenated mixture above, CuBr (4.3 mg, 3.007 x 10^{-5} mol) was added under positive argon flow. Then, the solution of **30** was added *via* syringe pump, at a flow rate of 0.06 mL min^{-1}, and after feeding the reaction was allowed to react for 2 h. The copper salts were removed by passage through activated basic alumina. The solvent was removed under reduced pressure. The crude product was purified using a Varian Pro-Star preparative SEC system equipped with a manual injector, differential refractive index detector, and single wave-length ultra-violet visible detector. Flow rate was maintained 10 mL min^{-1} and HPLC grade THF was used as the eluent. Separations were achieved using a PL gel 10 mm 1x103 Å, 300 mm x 25 mm preparative SEC column held at 25 °C. After injection, the expected fraction was collected manually with the yield as 39 mg. (yielding%=13.8 %).

SEC: (M_n=3380, M_p=3475, PDI=1.023)

MALDI-ToF MS: [**30**+Na^+]=3782.59 (Cal.=3782.89); [**30**+Na^++4O]=3846.06 (Cal.= 3846.87); [**30**+K^++4O]=3862.71 (Cal.= 3862.06)

Synthesis of 4-arm star-like EG-MKF conjugate 31

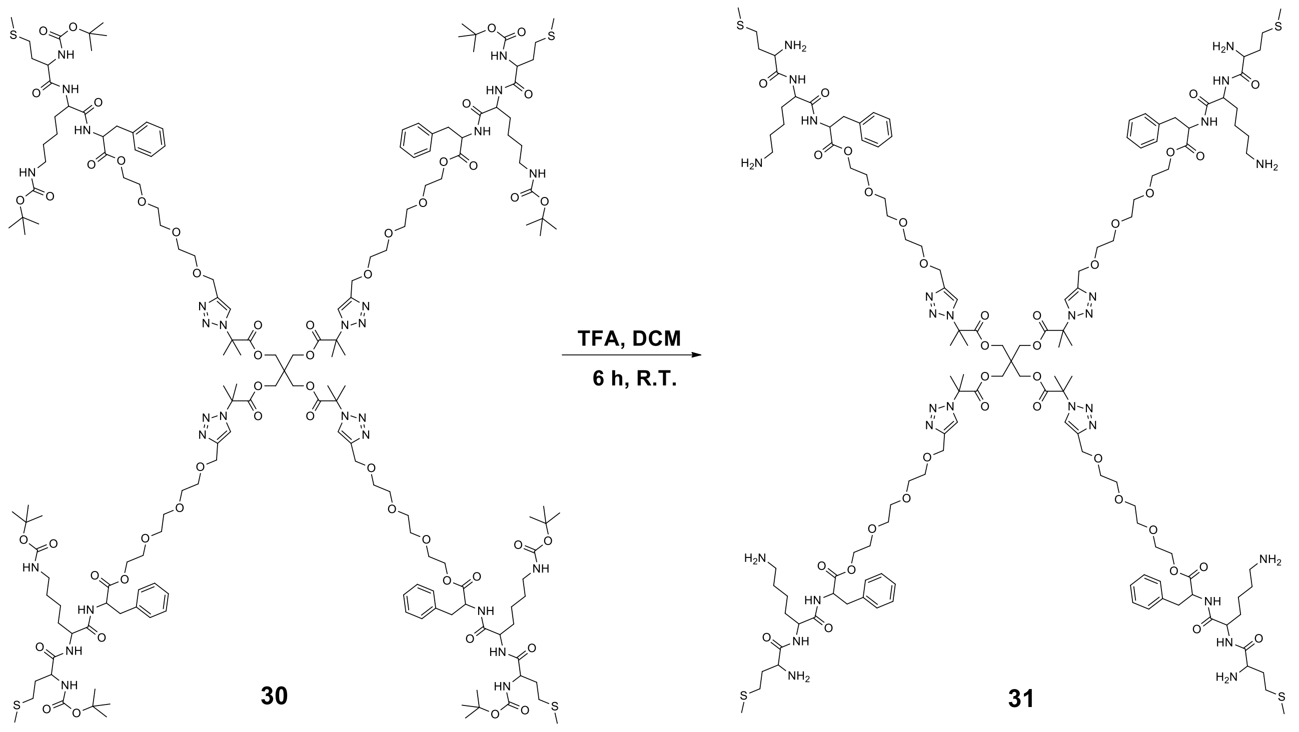

30 (0.015 g) was dissolved in 3 mL mixture of TFA and DCM (1:1, v:v). And the solution was kept stirring for 6 h at room temperature The solvent was remove by reduced pressure, and dried in high vacuum for 24 h, grey yellow oil **31** was produced with near quantitative conversion.

Supplementary Figures

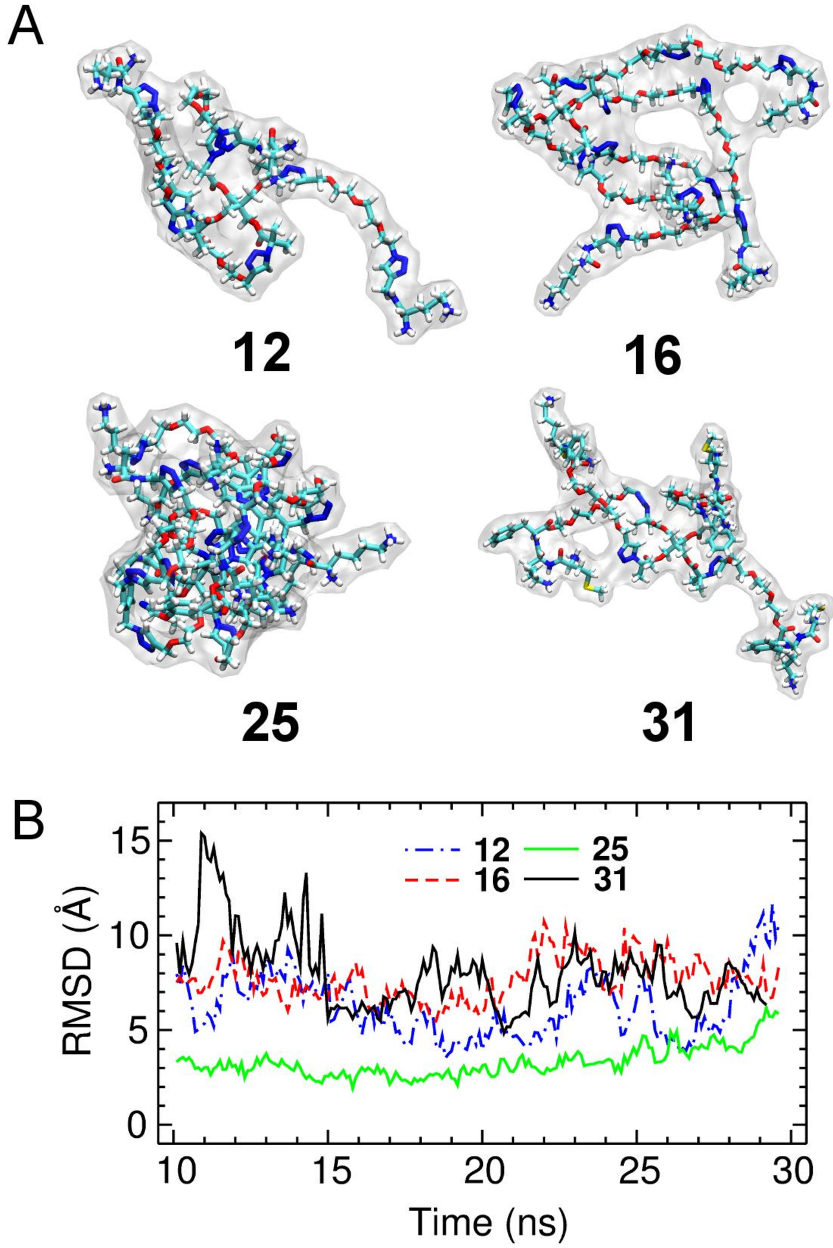

Fig A. Structure of four star molecules: 12, 16, 25 and 31. (A) Snapshot of **12**, **16**, **25** and **31** predicted from molecular dynamics. (B) Root mean square deviation (RMSD) of the heavy atoms of the four compounds in water with reference to their average structure over a simulation period of 30 ns. The first 10 ns was considered as equilibration and excluded from analysis.

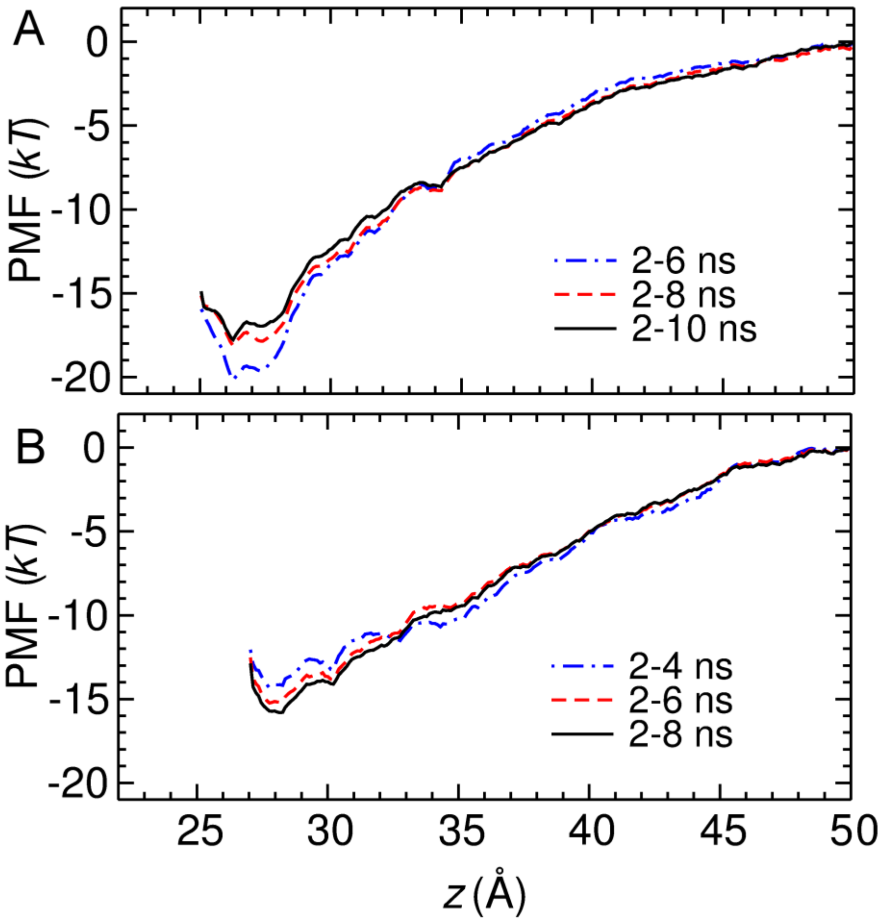

Fig B. **Block analysis showing the convergence of the the PMF profiles for the binding of 12 (A) and 31 (B) to Kv1.3.**

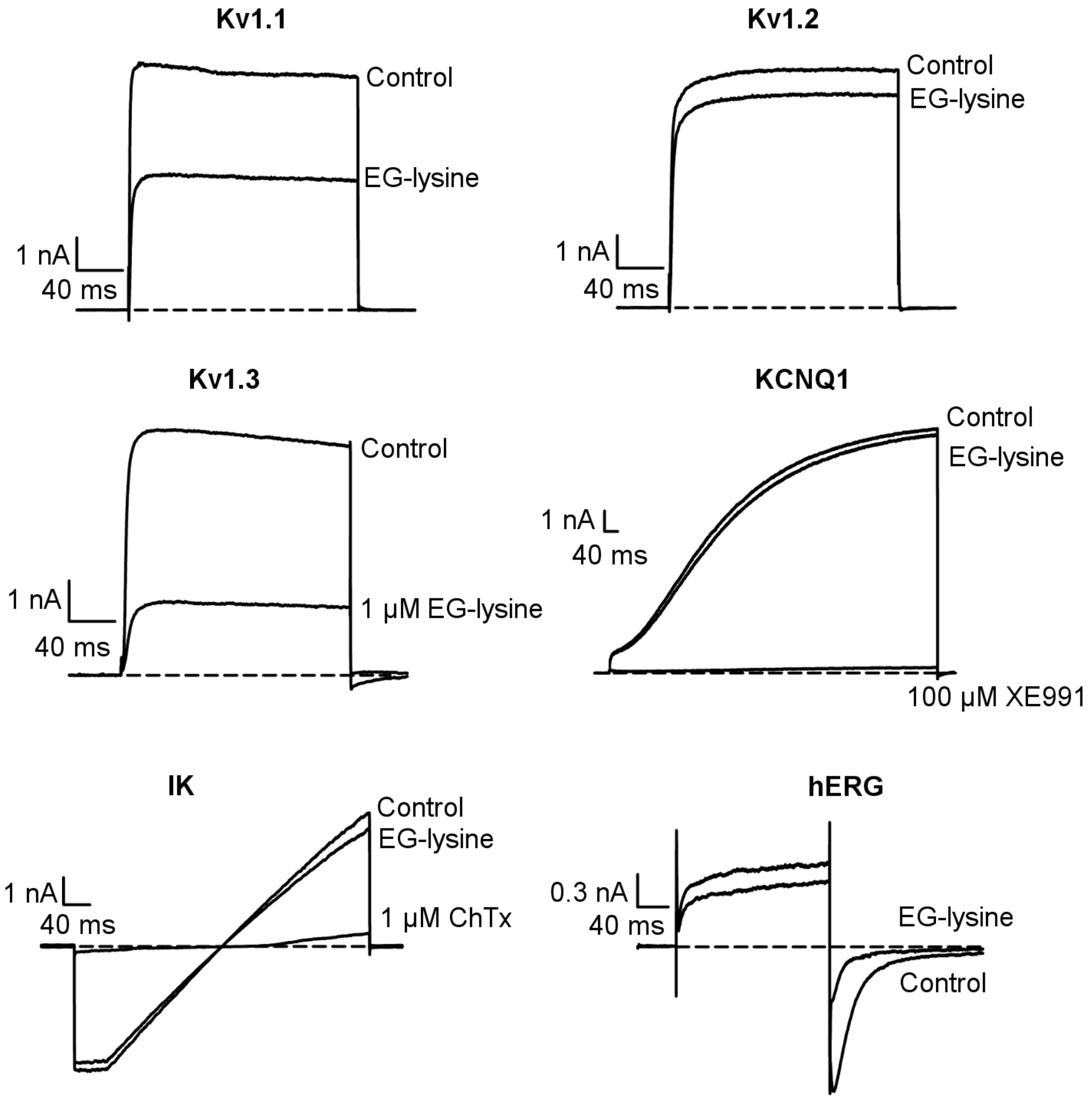

Fig C. Effect of EG-lysine 12 on the currents of six K$^+$ channels. The concentration of EG-lysine is 10 μM unless otherwise indicated. For KCNQ1 and IK, their known blockers XE991 and charybdotoxin (ChTx) are used as positive control, respectively.

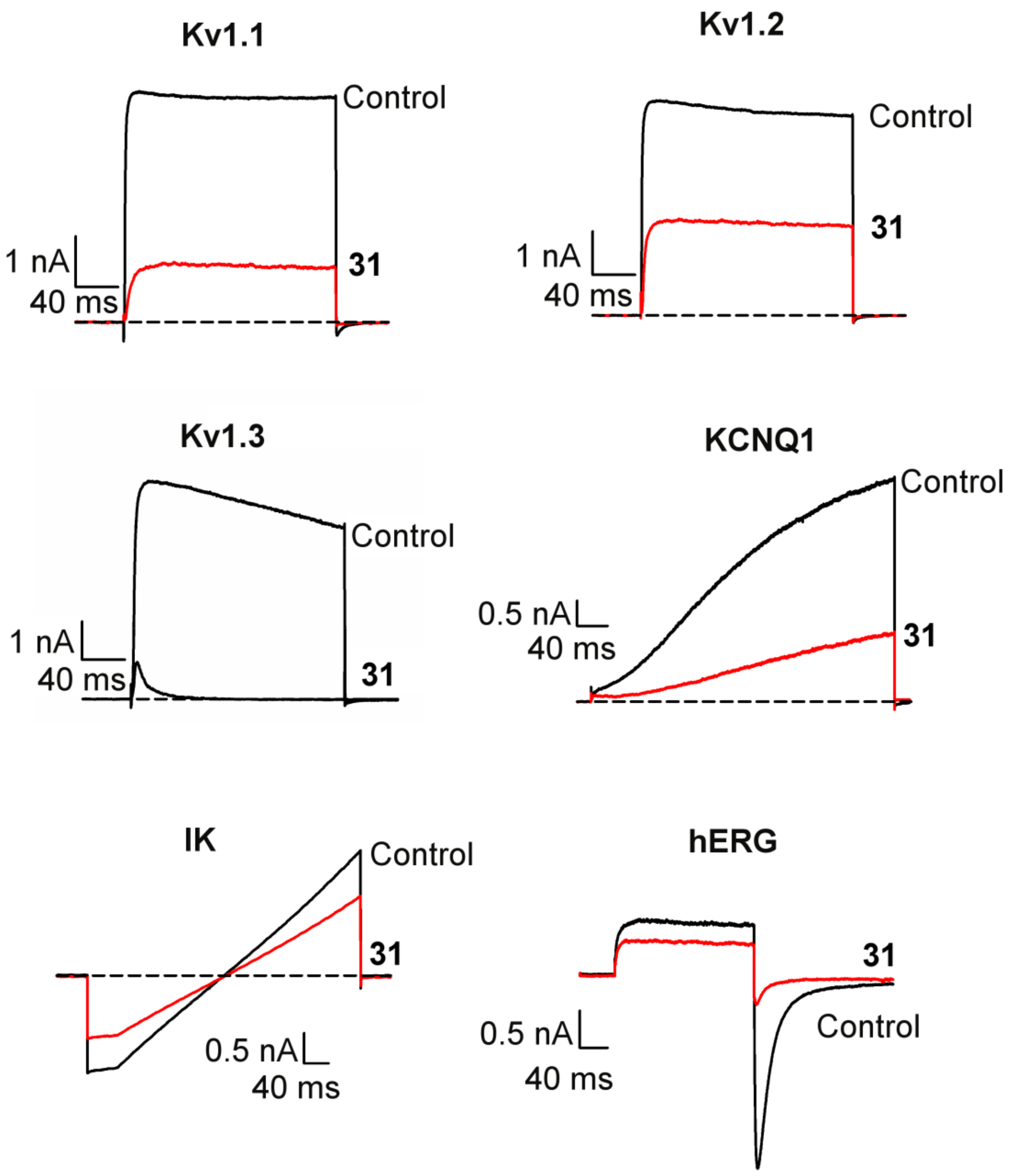

Fig D. **Representative current traces of six K^+ channel isoforms showing the block by 1 mM of 31.**

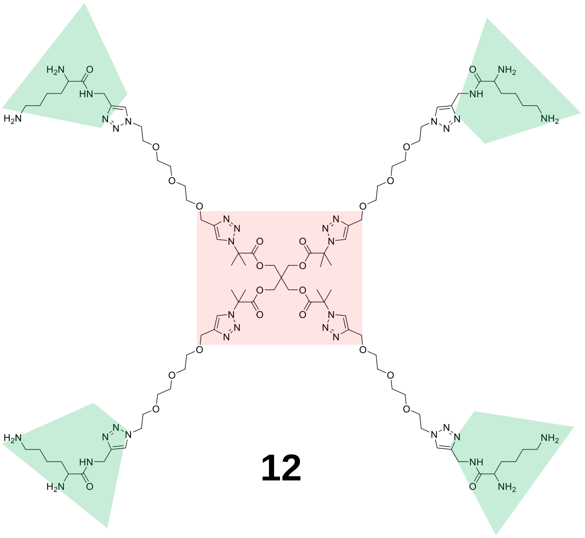

12

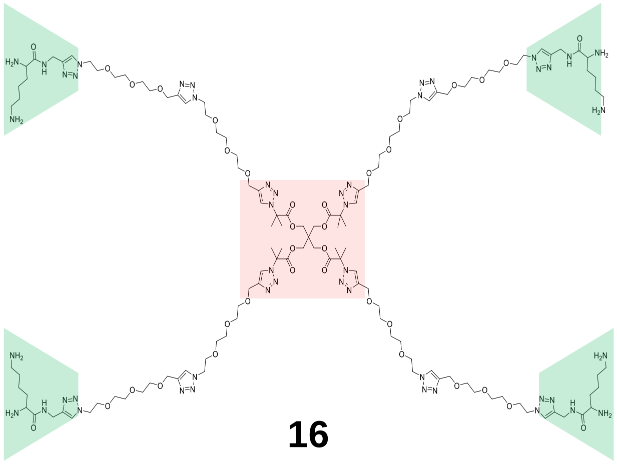

16

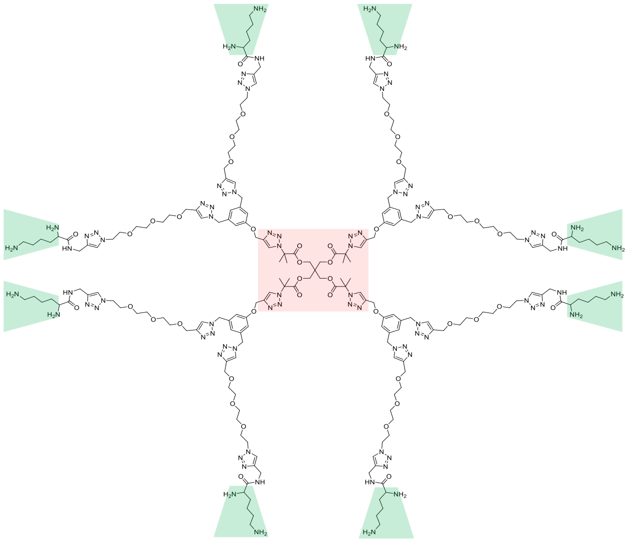

25

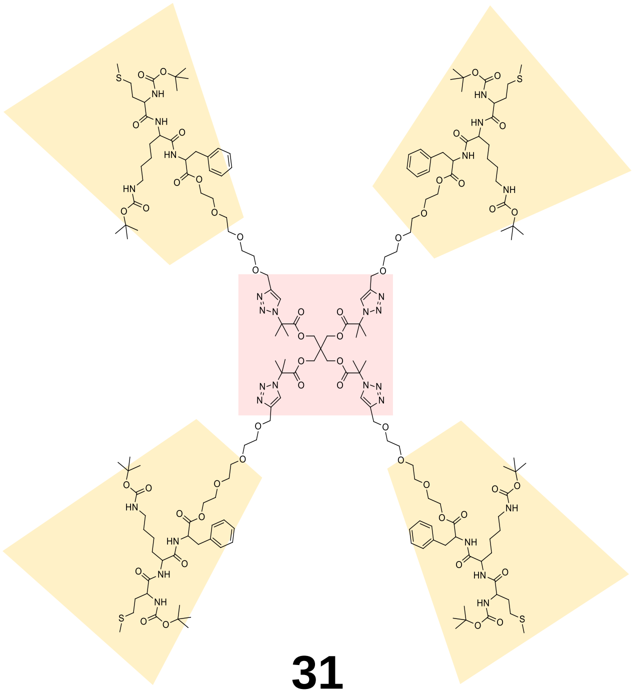

31

Fig E. **Chemical structures of four EG-peptide conjugates: 12, 16, 25 and 31.** The core common to the four conjugates is highlighted in purple.

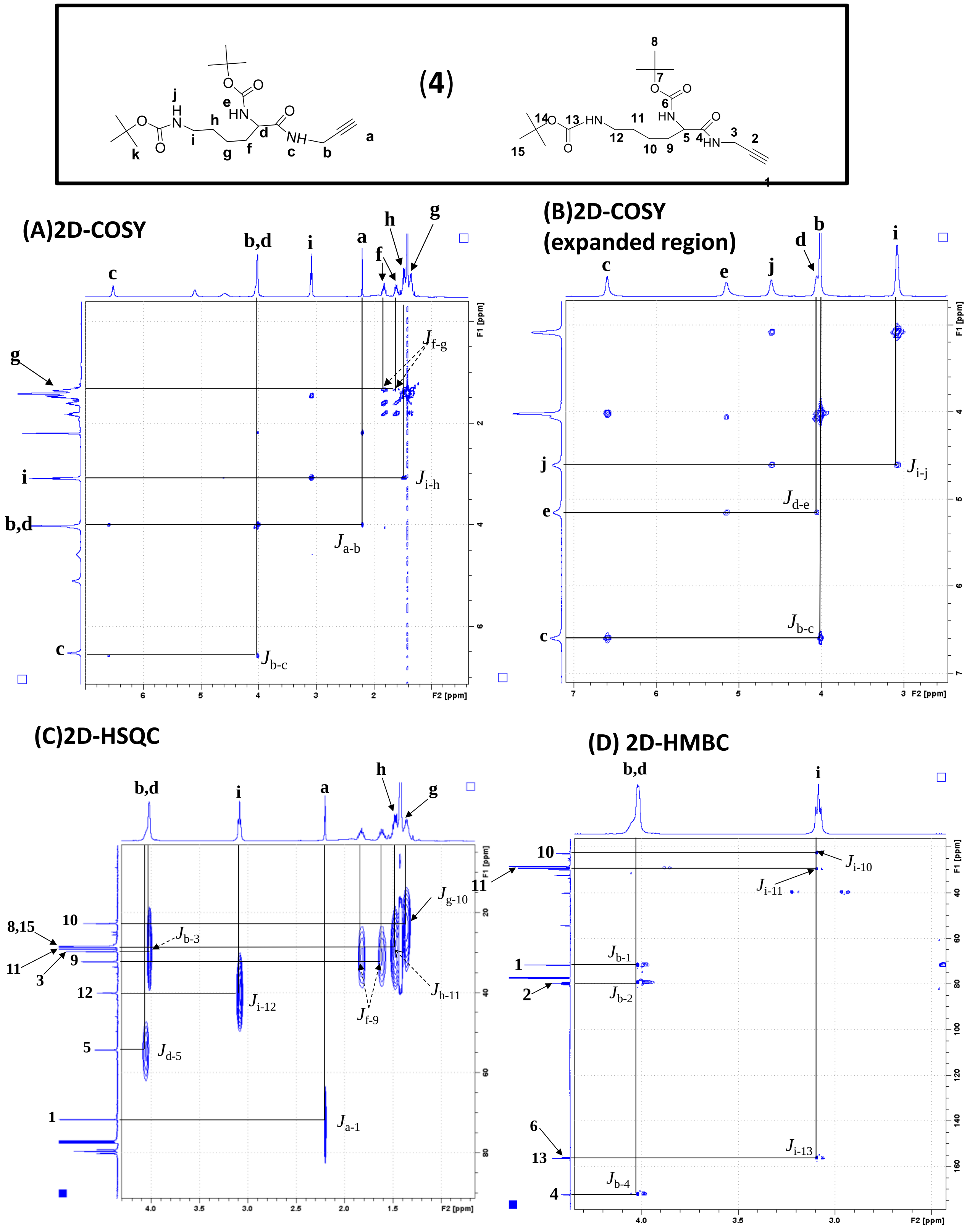

Fig F. NMR of 4 recorded in $CDCl_3$ at 298K on 500MHz spectrometer.

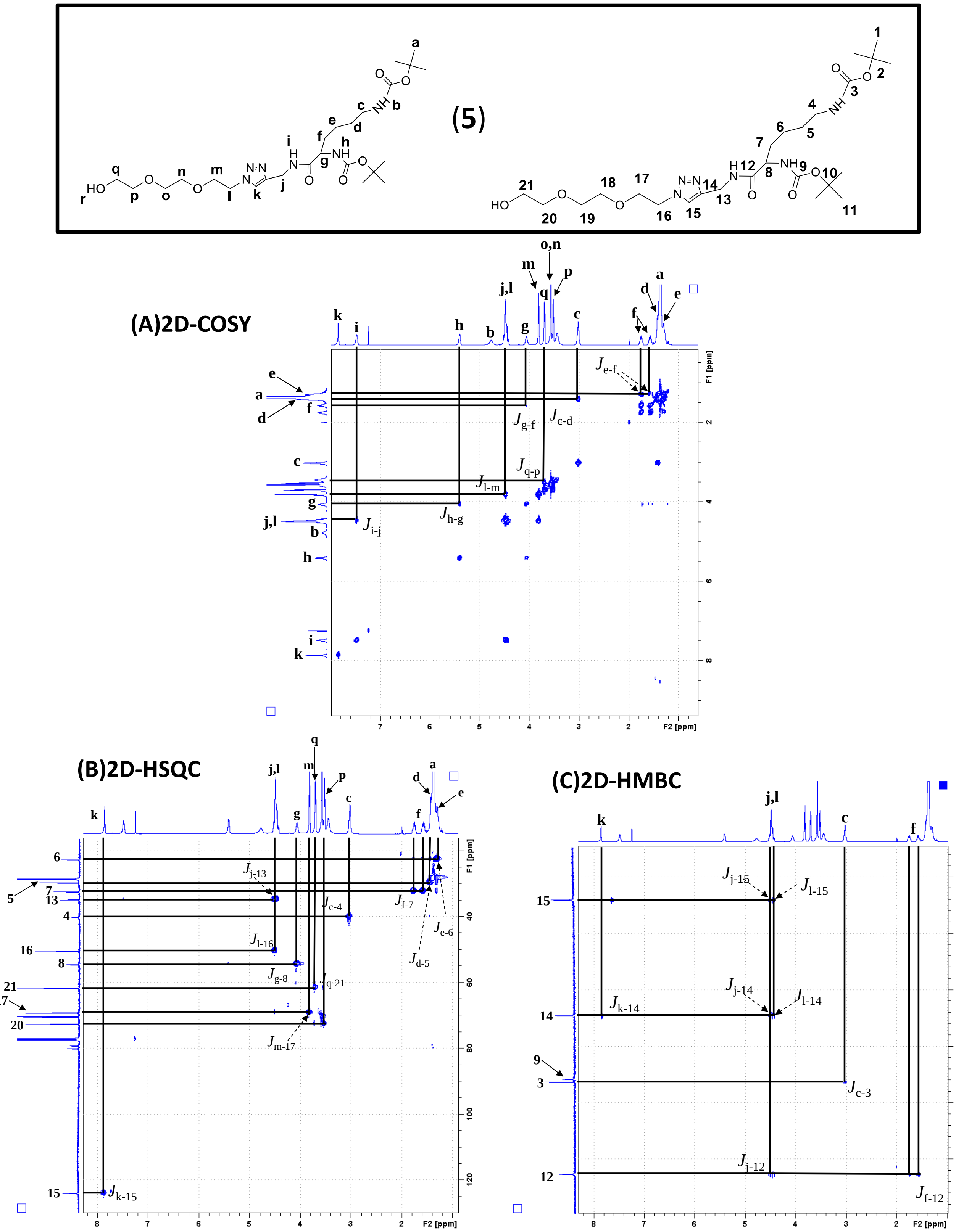

Fig G. NMR of 5 recorded in $CDCl_3$ at 298K on 500MHz spectrometer.

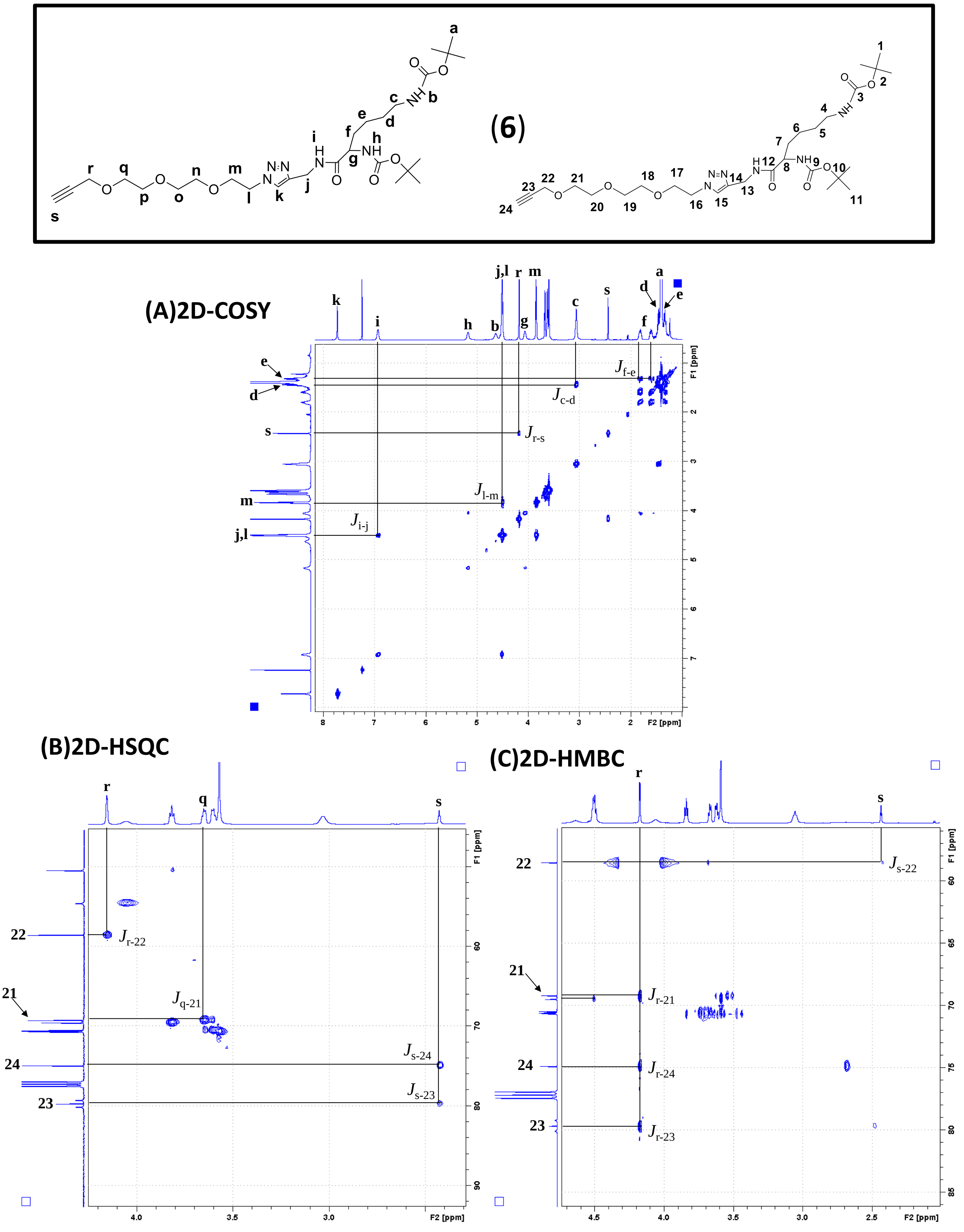

Fig H. NMR of 6 recorded in $CDCl_3$ at 298K on 500MHz spectrometer.

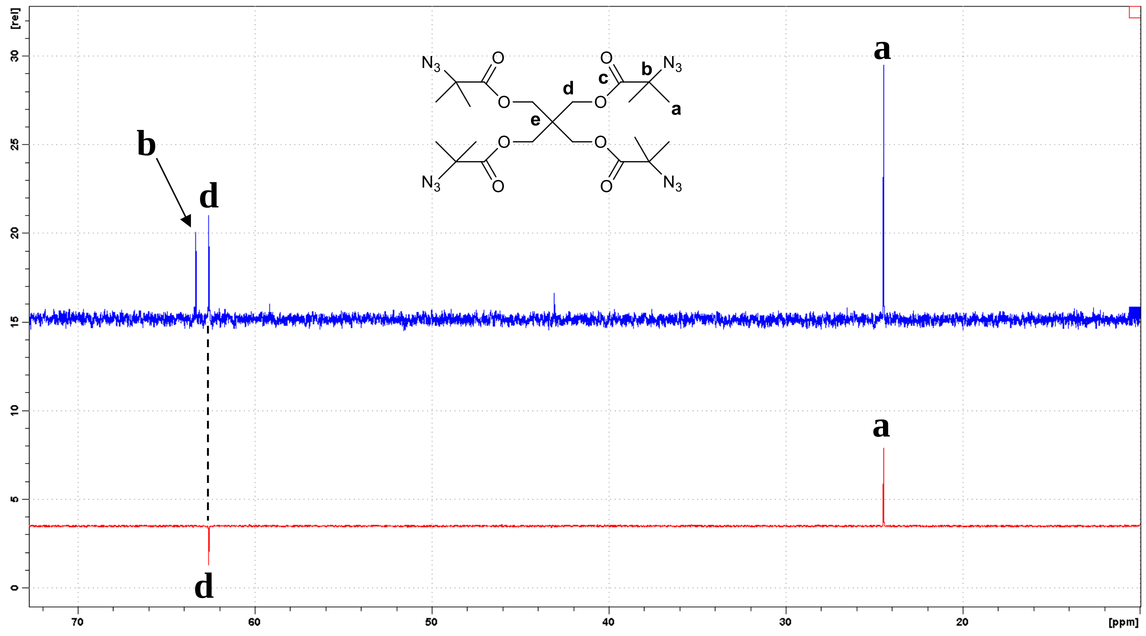

Fig I. NMR spectra of 10 recorded in $CDCl_3$ at 298 K, 125 MHz. (A) ^{13}C NMR, and (B) DEPT-135° NMR.

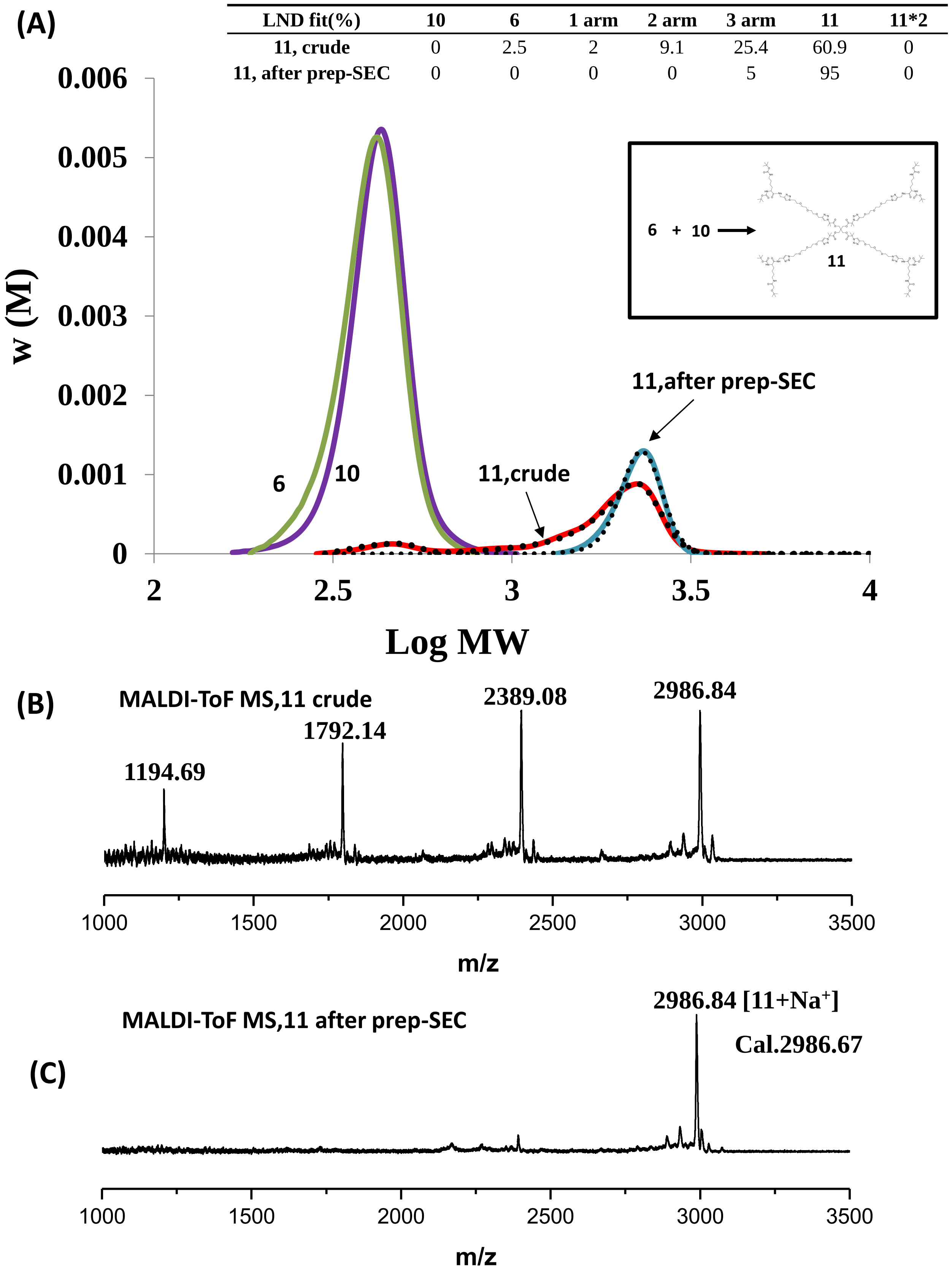

LND fit(%)	10	6	1 arm	2 arm	3 arm	11	11*2
11, crude	0	2.5	2	9.1	25.4	60.9	0
11, after prep-SEC	0	0	0	0	5	95	0

Fig J. (A) SEC traces of **6**, **10, 11** (crude and after prep) in THF and LND simulation of **11** (black dots). (B) MALDI-ToF MS of **11** crude, and (C) MALDI-ToF MS of **11** after prep. The spectrum was recorded in reflection mode using DCTB as the matrix and $NaCF_3COO$ as the cation source.

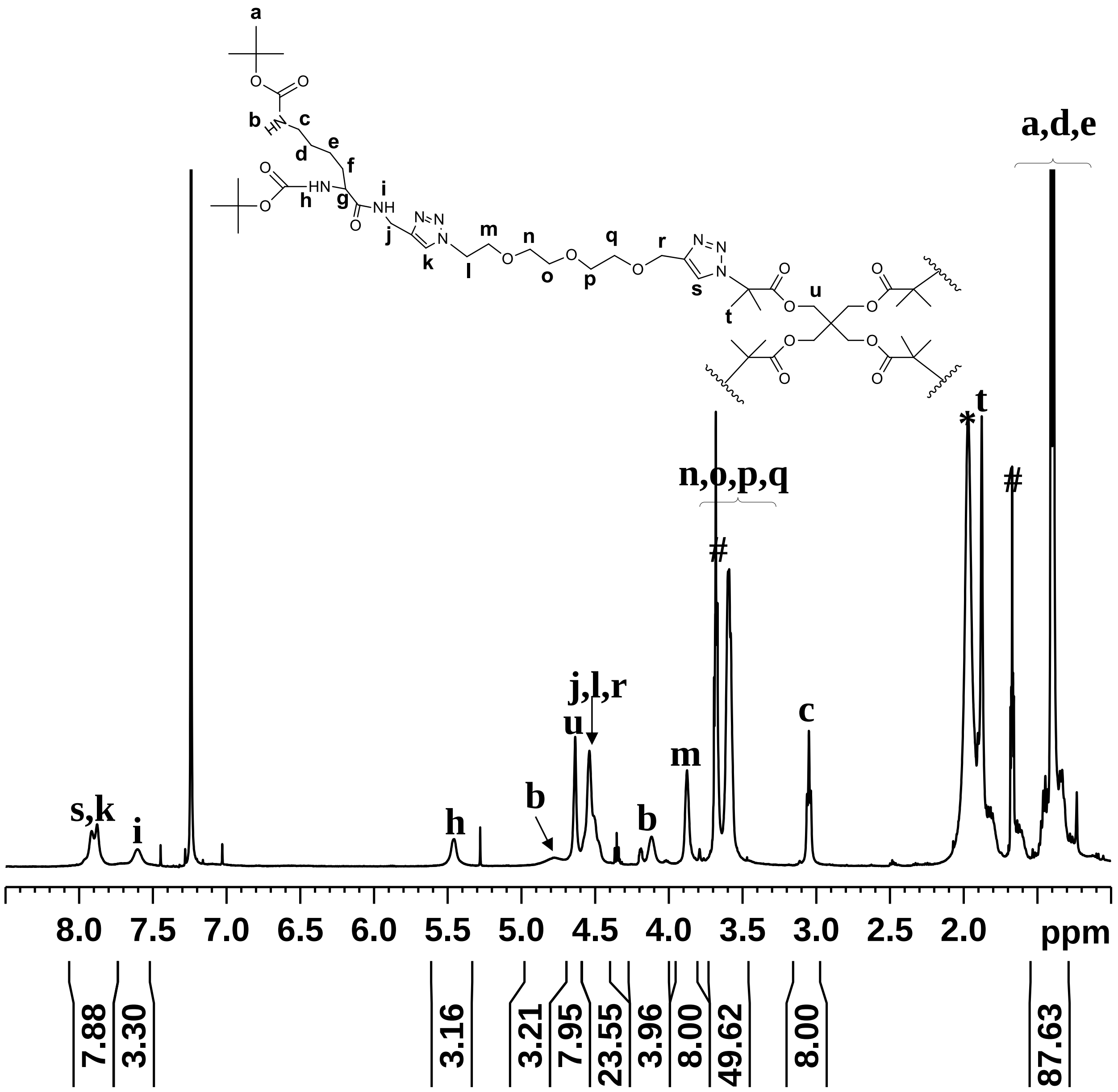

Fig K. ^{1}H NMR spectrum of 11, recorded in $CDCl_3$ at 298 K, 500 MHz. *=H_2O,#=THF.

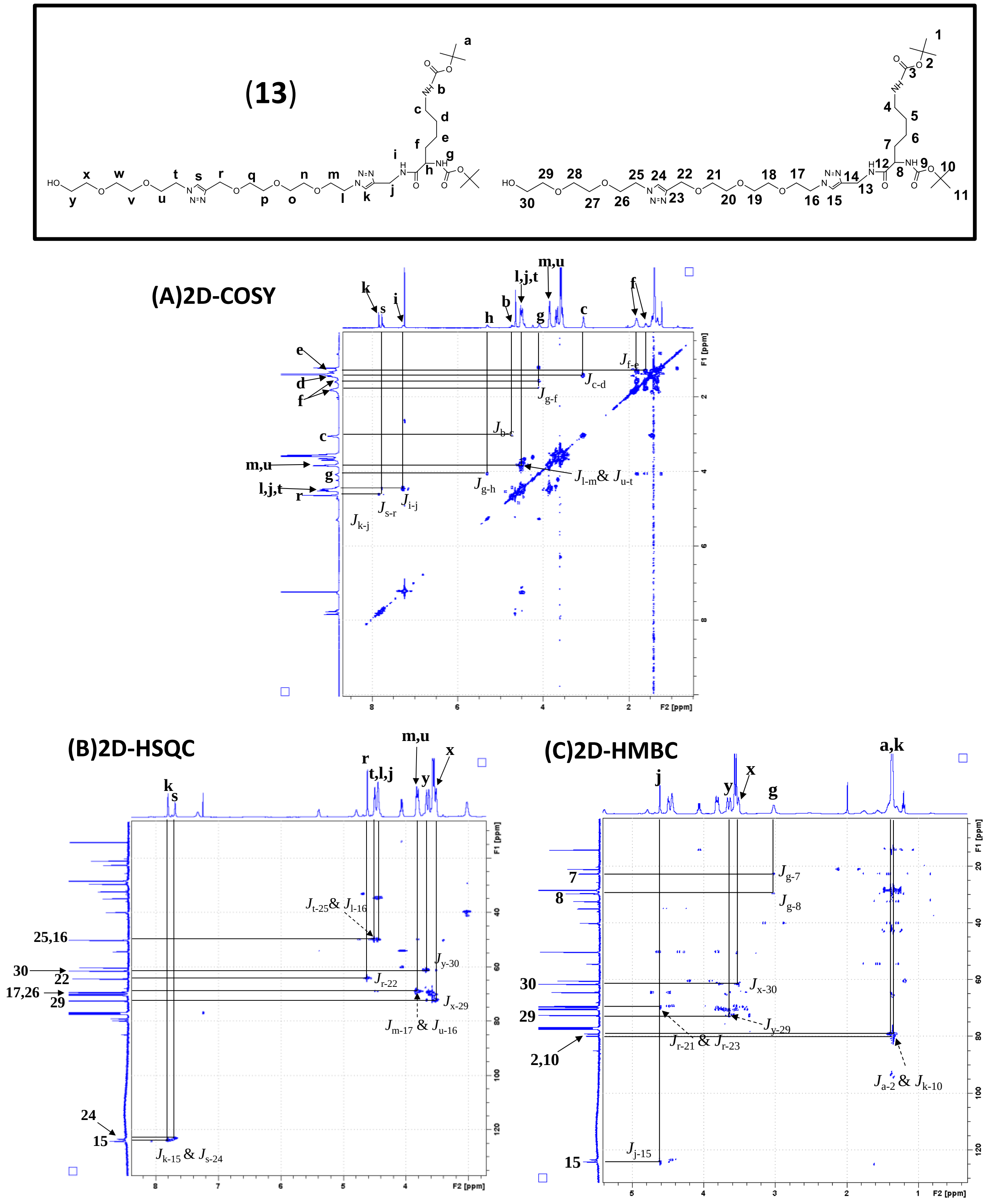

Fig L. NMR of 13 recorded in $CDCl_3$ at 298K on 500MHz spectrometer.

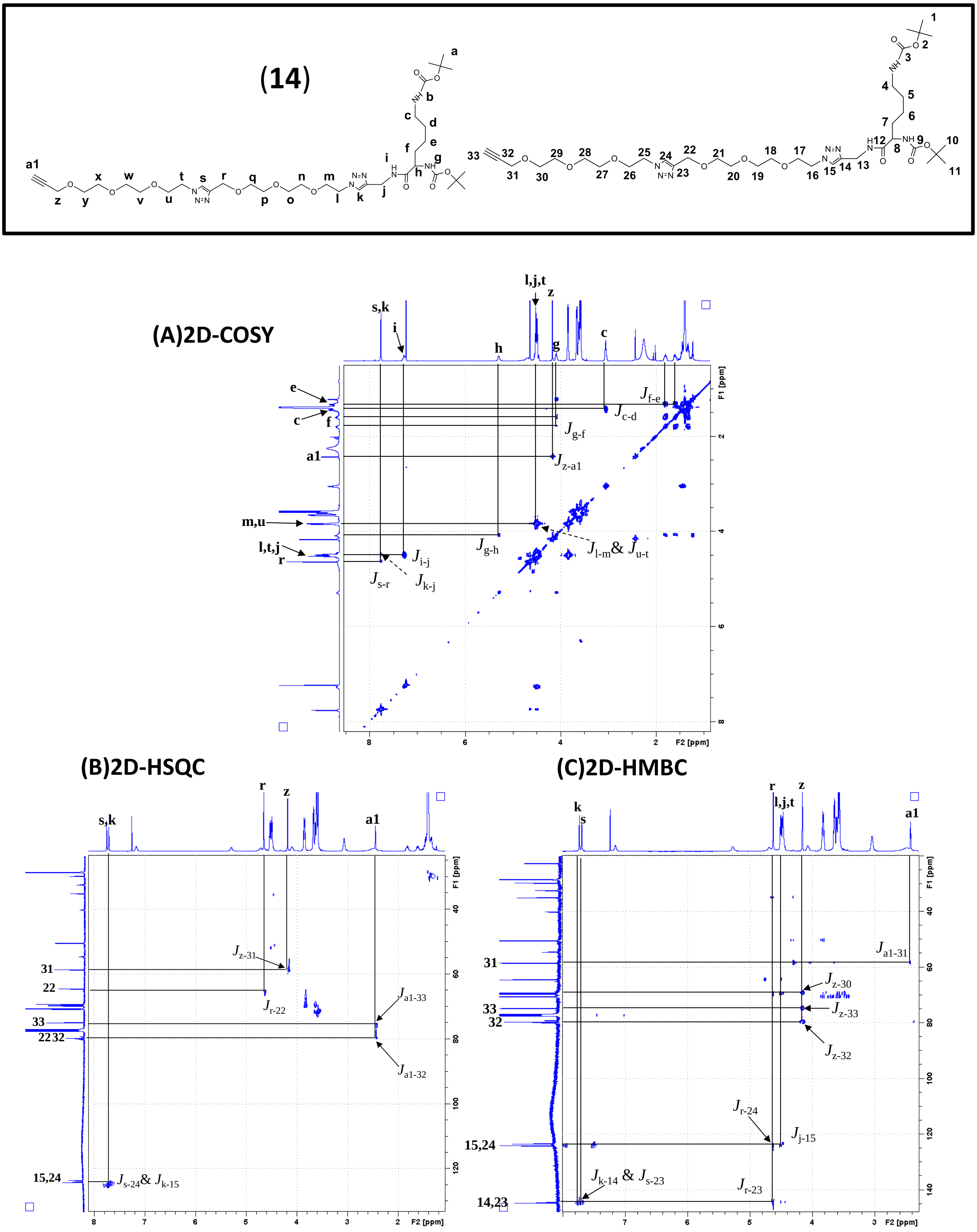

Fig M. NMR of 14 recorded in $CDCl_3$ at 298K on 500MHz spectrometer.

LND fit(%)	10	14	1 arm	2 arm	3 arm	15	15*2
**15, crude**	6.3	45	6.3	3.6	3.1	35.6	0
**15, after prep-SEC**	0	0	0	0	3	97	0

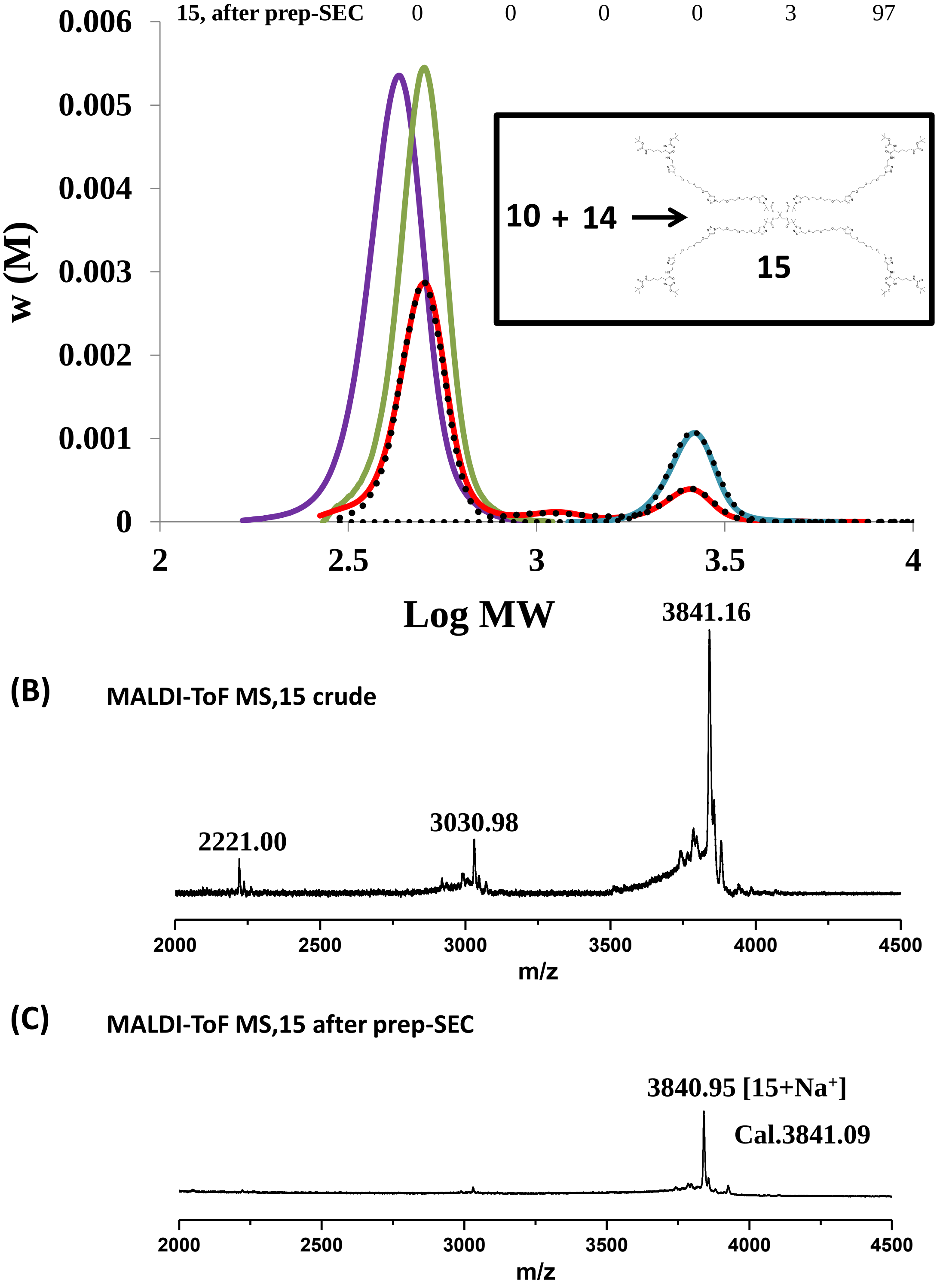

Fig N. (A) SEC traces of **10**, **14, 15** (crude and after prep) in THF and LND simulation of **15** (black dots), (B) MALDI-ToF MS of **15** crude, (C) MALDI-ToF MS of **15** after prep-SEC. The spectrum was recorded in reflection mode using DCTB as the matrix and $NaCF_3COO$ as the cation source.

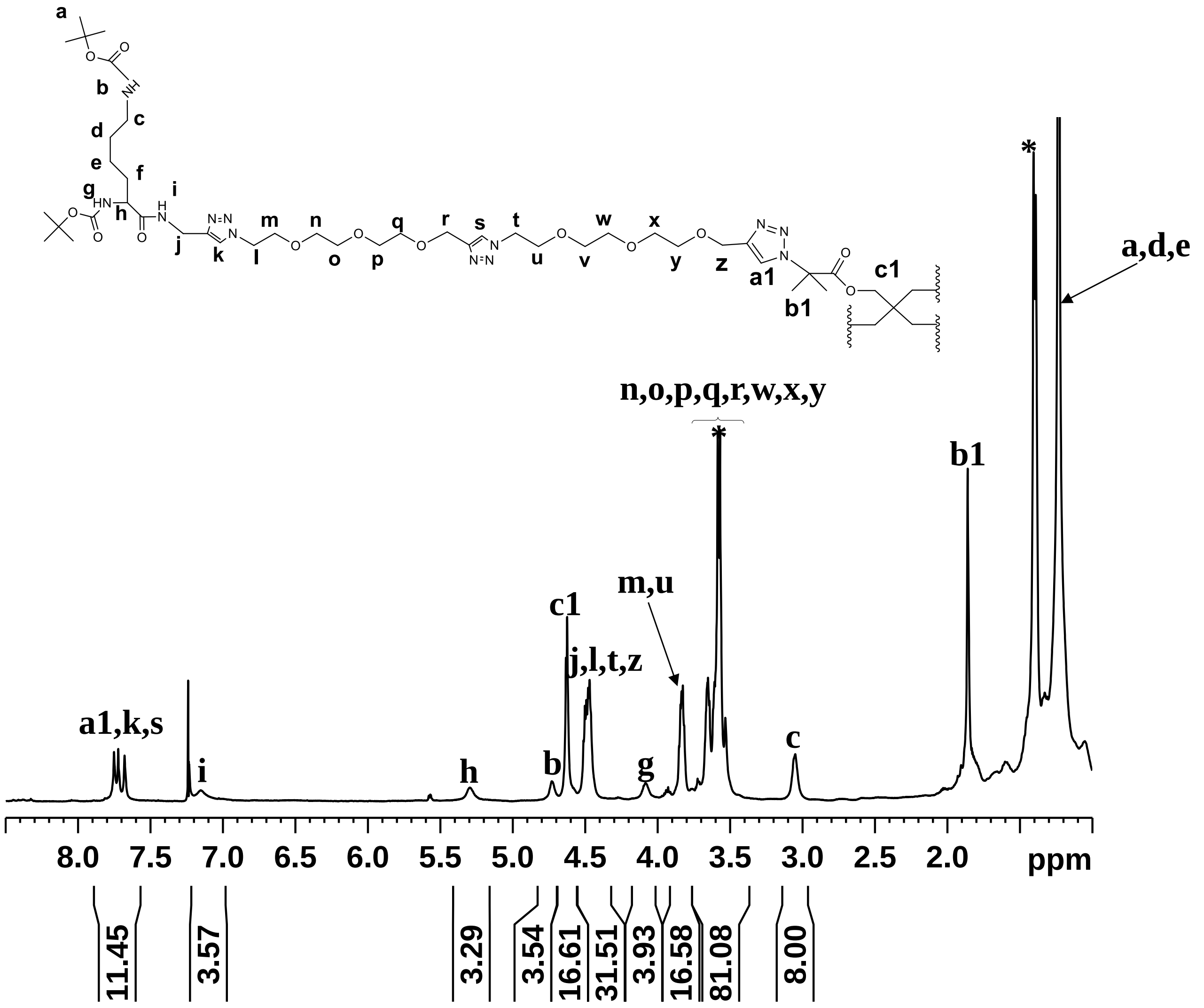

Fig O. ^{1}H NMR spectrum of 15, recorded in $CDCl_3$ at 298 K, 500 MHz. *THF.

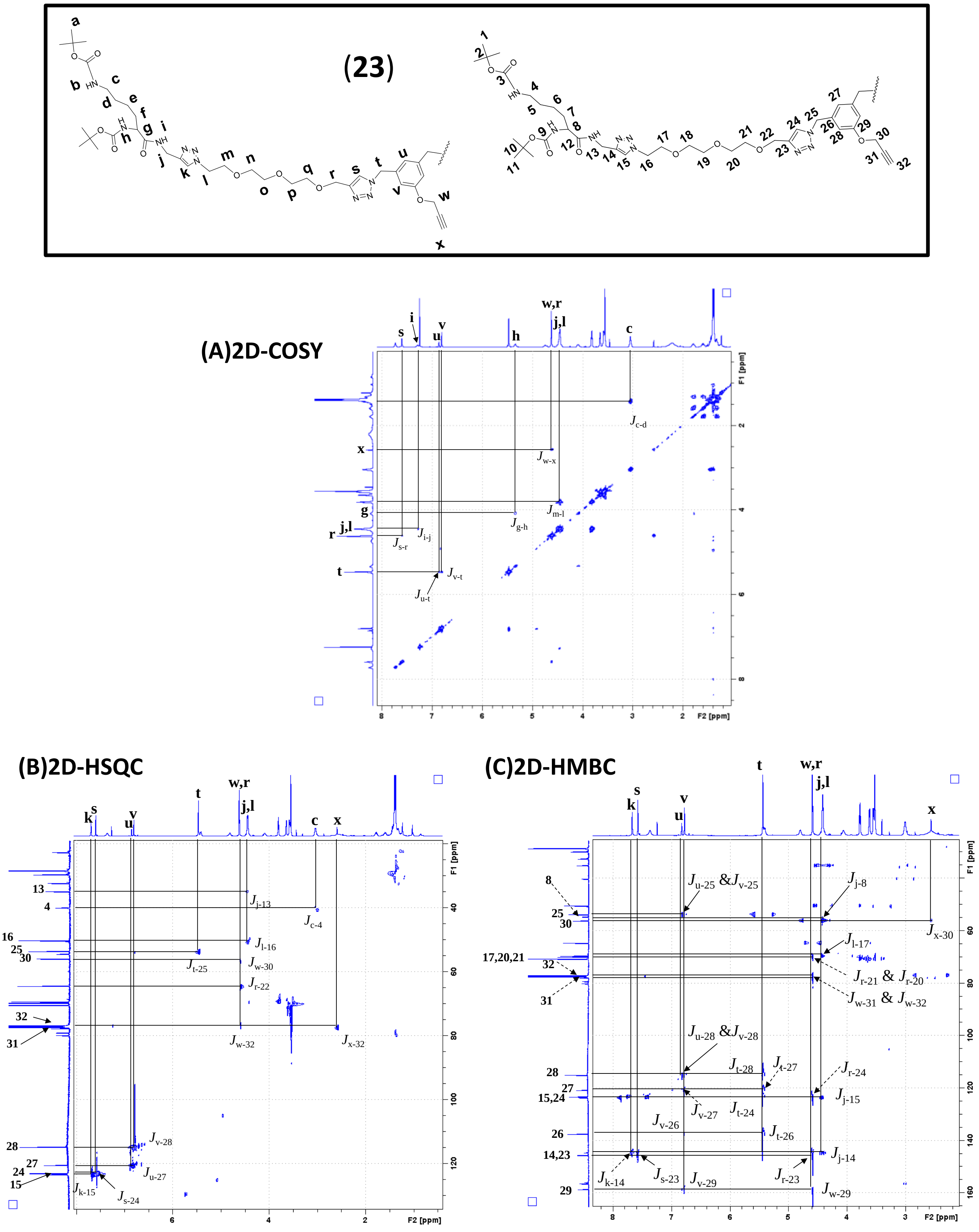

Fig P. NMR spectra of 23, recorded in $CDCl_3$ at 298K on 500MHz spectrometer.

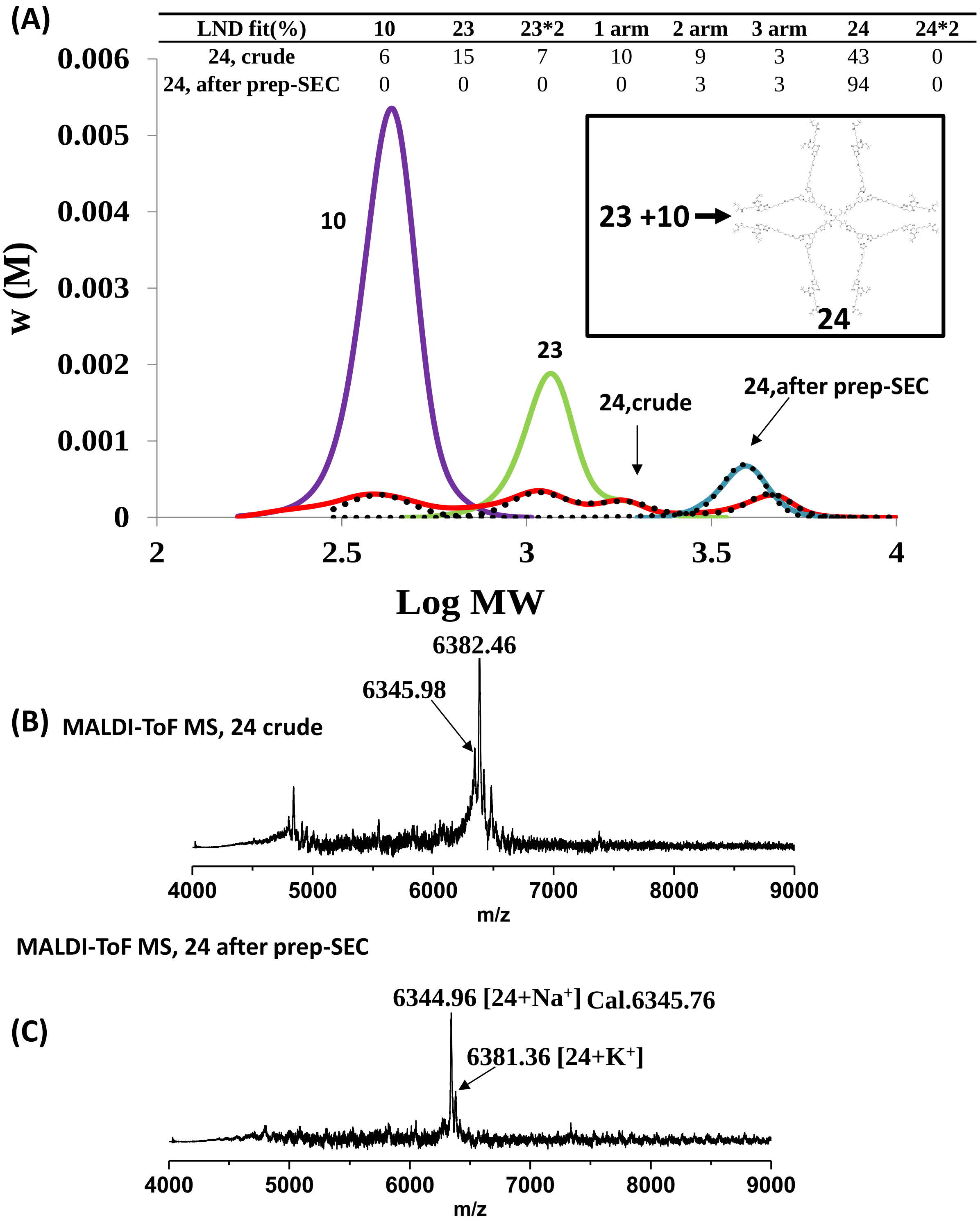

Fig Q. (A) SEC traces of **10**, **23, 24** (crude and after prep) in THF and LND simulation of **24** , PSTY as standard, (B) MALDI-ToF MS of **24** crude, (C) MALDI-ToF MS of **24** after prep-SEC. the spectrum was recorded in linear mode using DCTB as the matrix and $NaCF_3COO$ as the cation source.

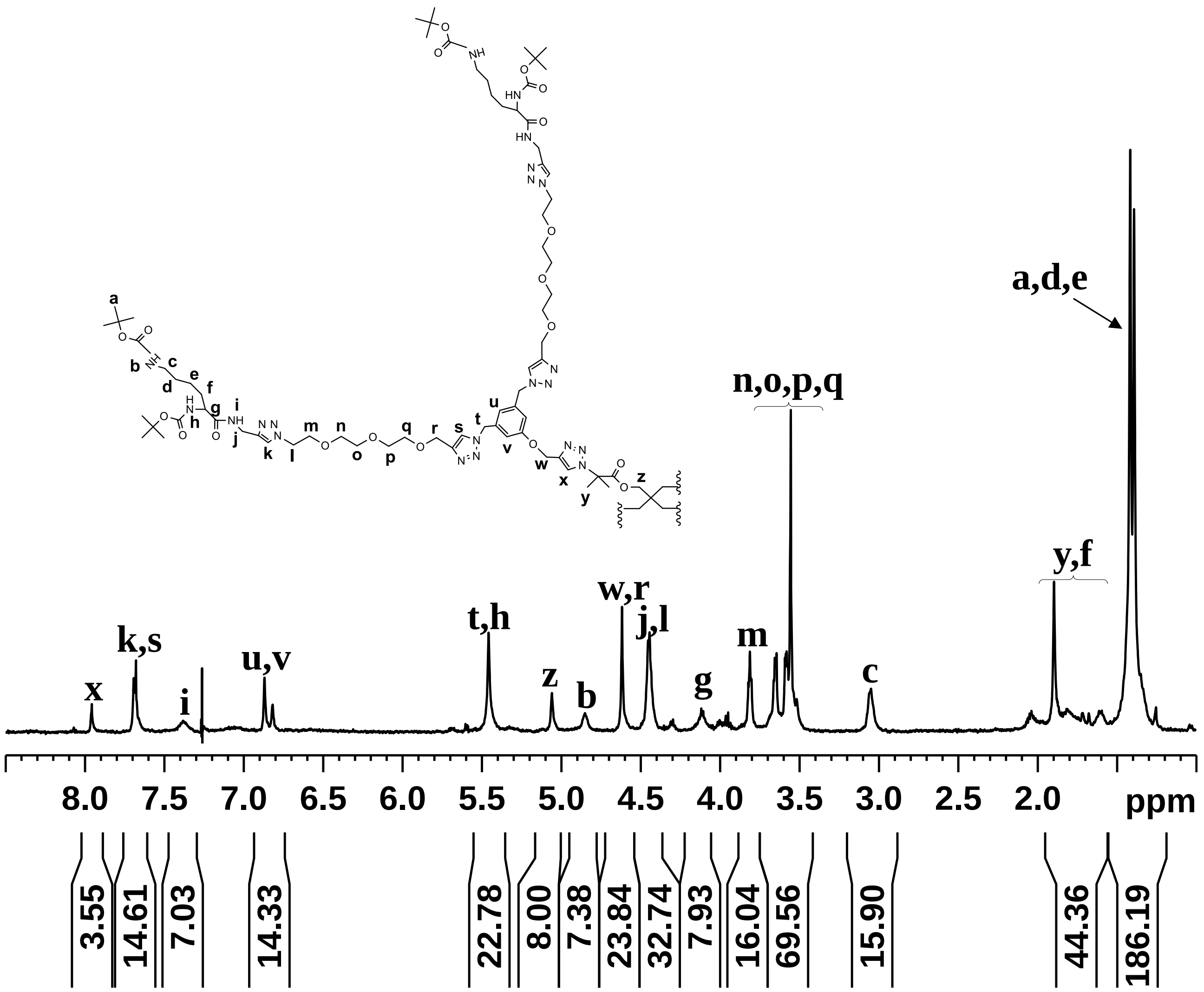

Fig R. ^{1}H 1D DOSY NMR spectrum of 24, recorded in $CDCl_3$ at 298 K, 500 MHz.

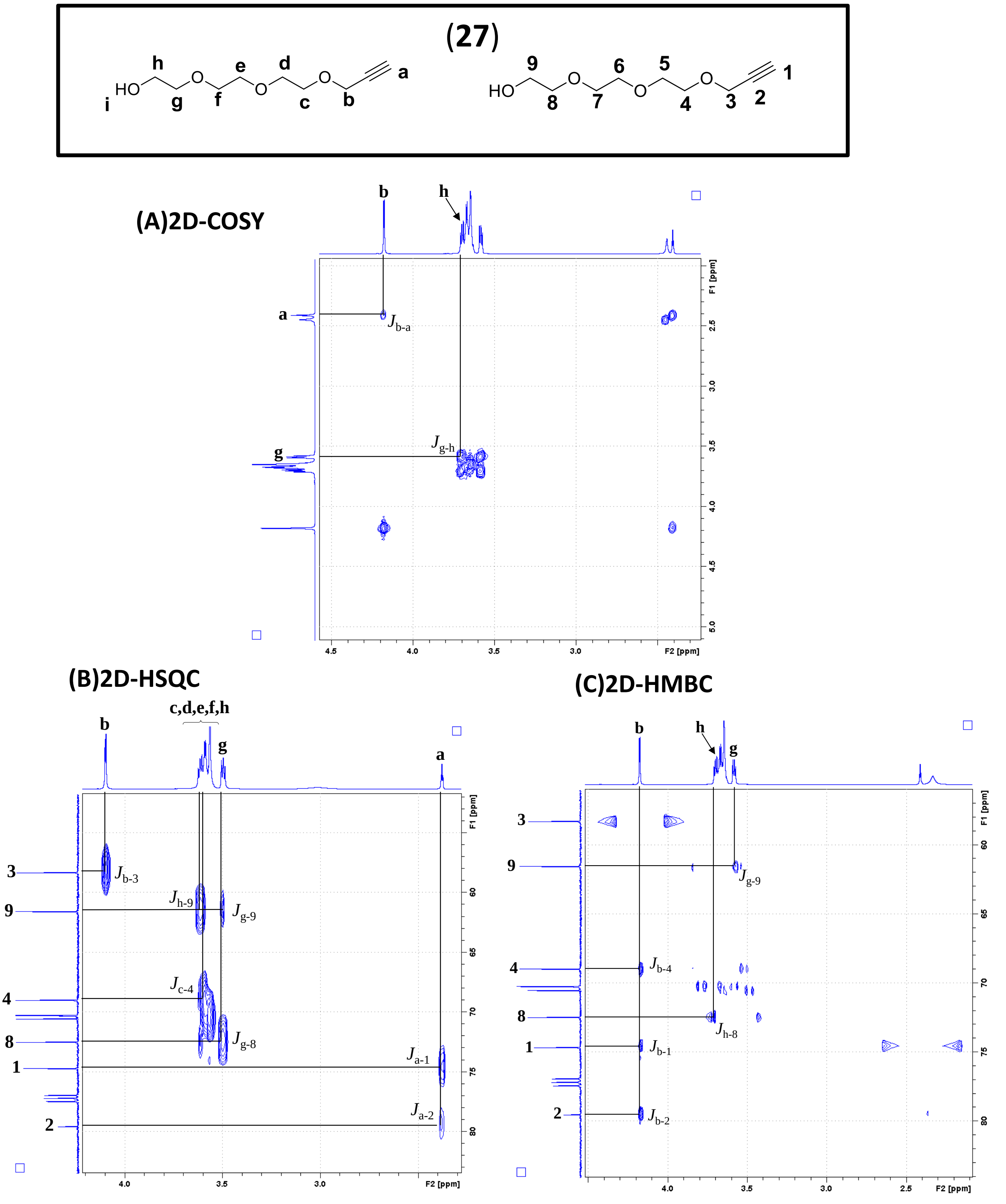

Fig S. NMR spectra of 27, recorded in CDCl3 at 298K on 500MHz spectrometer.

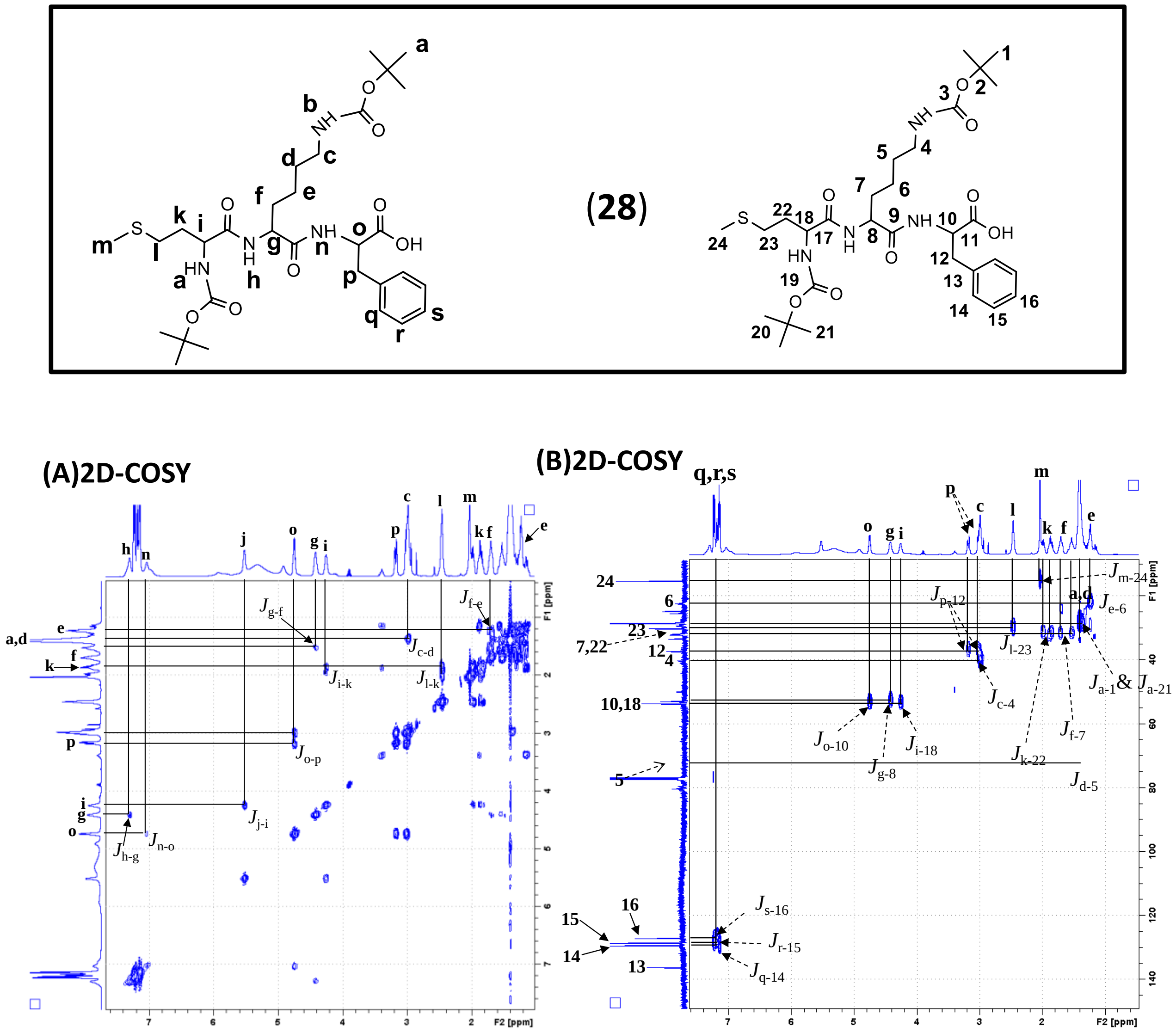

Fig T. NMR spectra of 28, recorded in CDCl3 at 298K on 500MHz spectrometer.

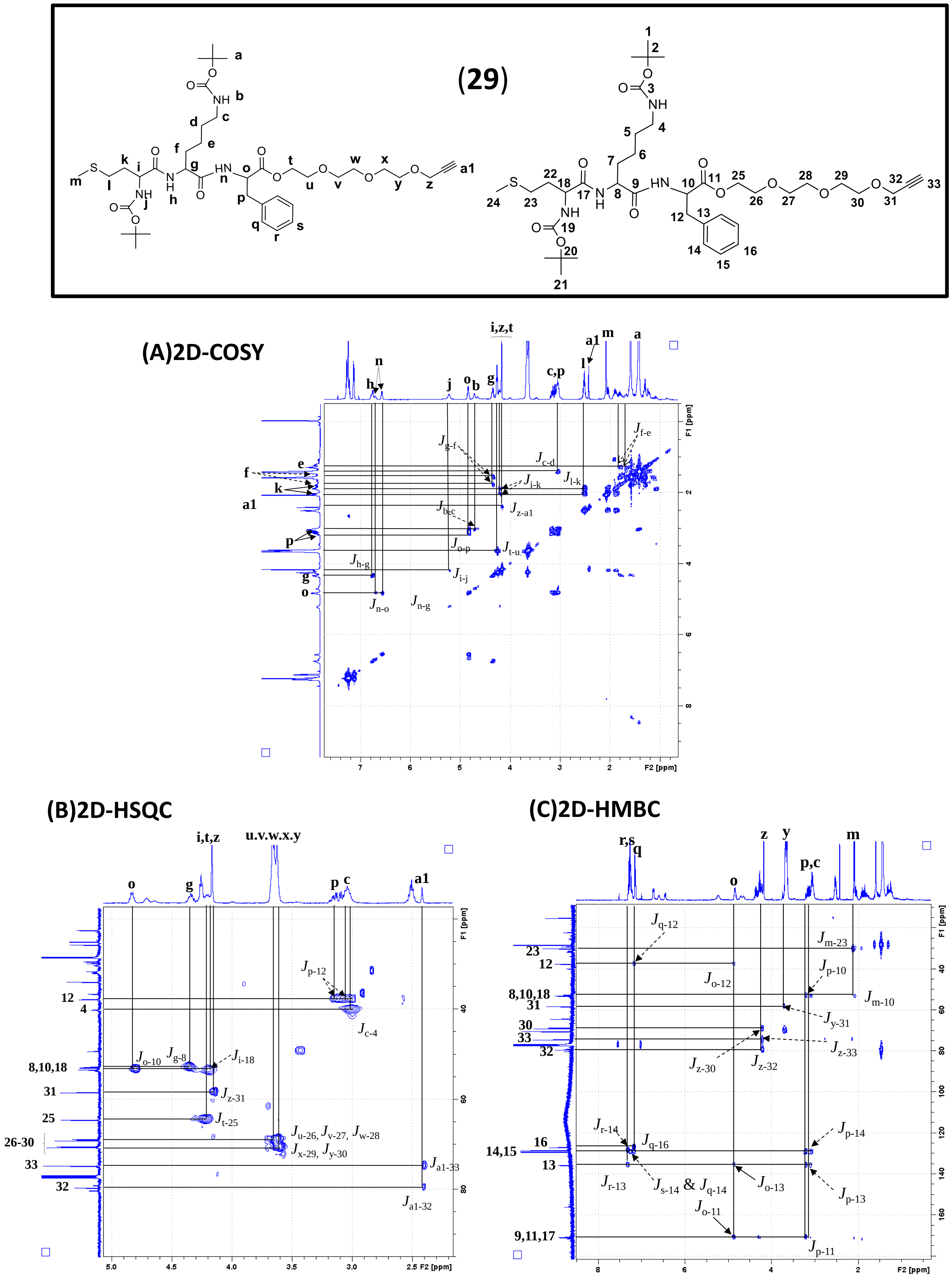

Fig U. NMR spectra of 29, recorded in CDCl3 at 298K on 500MHz spectrometer.

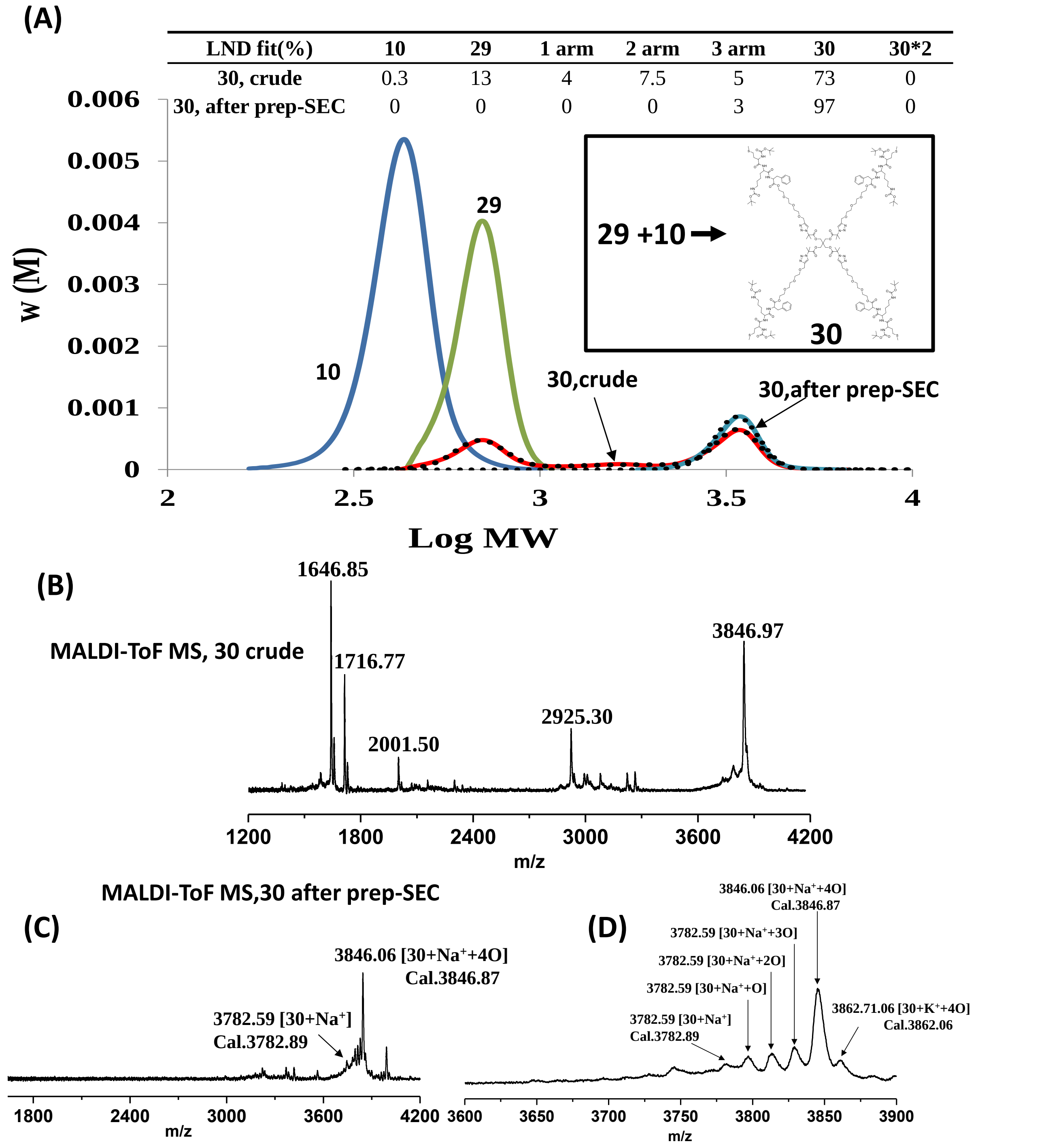

LND fit(%)	10	29	1 arm	2 arm	3 arm	30	30*2
30, crude	0.3	13	4	7.5	5	73	0
30, after prep-SEC	0	0	0	0	3	97	0

Fig V. (A) SEC traces of **10**, **29,** **30** (crude and after prep) in THF and LND simulation of **30** (black dots), (B) MALDI-ToF MS of **30** crude, (C) MALDI-ToF MS of **30** after prep-SEC, and (D) expanded region of (C). The spectrum was recorded in reflection mode using DCTB as the matrix and $NaCF_3COO$ as the cation source.

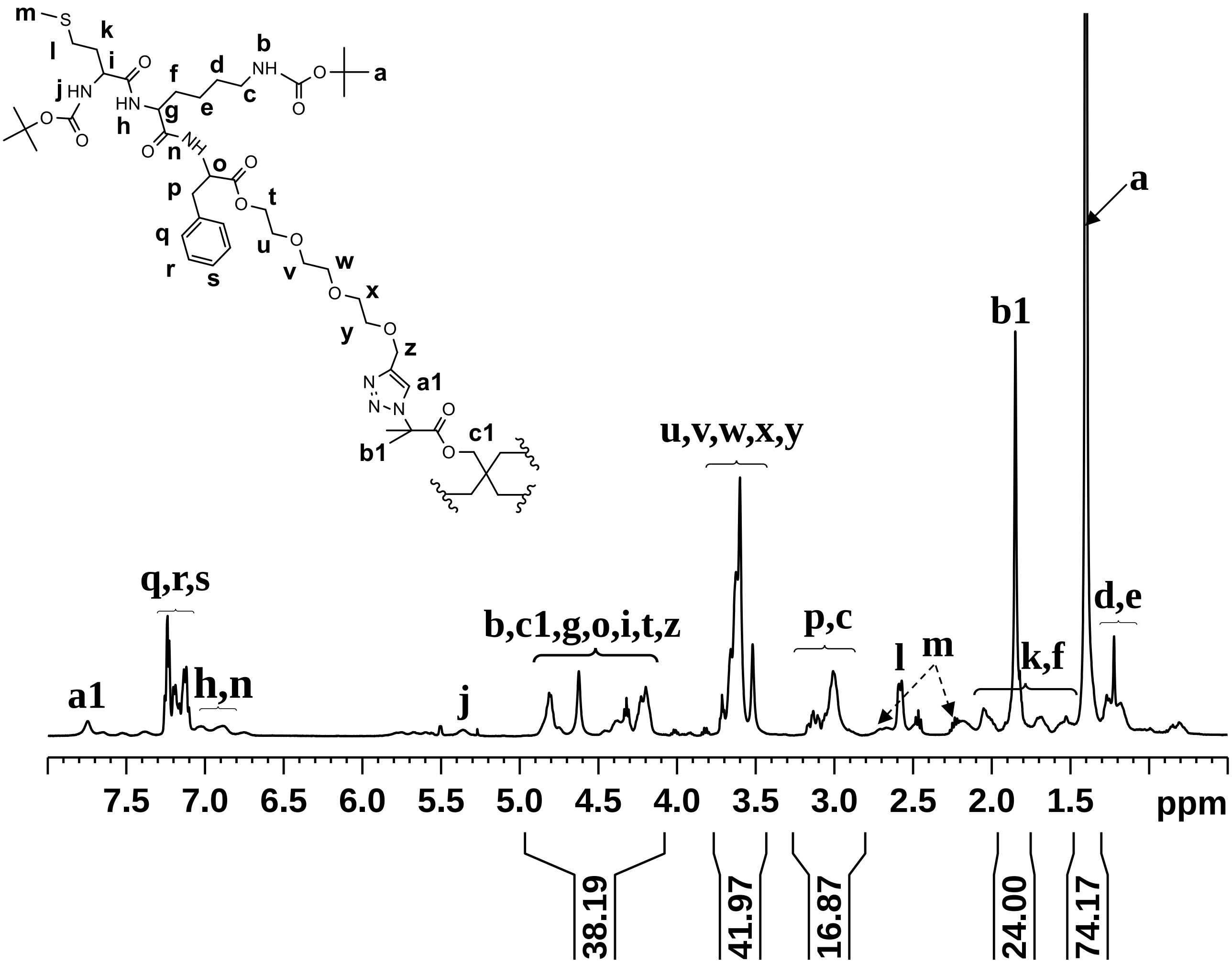

Fig W. ^{1}H NMR spectrum of 30, recorded in $CDCl_3$ at 298 K, 500 MHz.

References

1. Phillips JC, Braun R, Wang W, Gumbart J, Tajkhorshid E, Villa E, et al. Scalable molecular dynamics with NAMD. J Comput Chem. 2005;26:1781-802.

2. Klauda JB, Venable RM, Freites JA, O'Connor JW, Tobias DJ, Mondragon-Ramirez C, et al. Update of the CHARMM all-atom additive force field for lipids: validation on six lipid types. J Phys Chem B. 2010;114:7830-43.

3. MacKerell AD, Bashford D, Bellott M, Dunbrack RL, Evanseck JD, Field MJ, et al. All-atom empirical potential for molecular modeling and dynamics studies of proteins. J Phys Chem B. 1998;102:3586-616.

4. Jorgensen WL, Chandrasekhar J, Madura JD, Impey RW, Klein ML. Comparison of simple potential functions for simulating liquid water. J Chem Phys. 1982;79:926-35.

5. Ryckaert JP, Ciccotti G, Berendsen HJC. Numerical integration of the cartesian equations of motion of a system with constraints: molecular dynamics of *n*-alkanes. J Comput Phys. 1977;23:327-41.

6. Miyamoto S, Kollman PA. SETTLE: An analytical version of the SHAKE and RATTLE algorithm for rigid water models. J Comput Chem. 1992;13:952-62.

7. Martyna GJ, Tobias DJ, Klein ML. Constant pressure molecular dynamics algorithms. J Chem Phys. 1994;101:4177-89.

8. Gavrilov M, Monteiro MJ. Derivation of the molecular weight distributions from size exclusion chromatography. Eur Polym J. 2015;65:191-6.

9. Monteiro MJ. Fitting molecular weight distributions using a log-normal distribution model. Eur Polym J. 2015;65:197-201.

10. Chen R, Robinson A, Gordon D, Chung SH. Modeling the binding of three toxins to the voltage-gated potassium channel (Kv1.3). Biophys J. 2011;101:2652-60.

11. Chen R, Chung SH. Binding modes of μ-conotoxin to the bacterial sodium channel (Na_VAb). Biophys J. 2012;102:483-8.

12. Finol-Urdaneta RK, Glavica R, McArthur JR, French RJ. Polymodal, high affinity actions of μ-conotoxins on a bacterial voltage-gated sodium channel. Biophys J. 2013;104:136a-7a.

13. Chen R, Chung SH. Conserved functional surface of anti-mammalian scorpion β-toxins. J Phys Chem B. 2012;116:4796-800.

14. Lu DR, Hossain MD, Jia ZF, Monteiro MJ. One-Pot Orthogonal Copper-Catalyzed Synthesis and Self-Assembly of L-Lysine-Decorated Polymeric Dendrimers. Macromolecules. 2015;48:1688-702.

15. Hossain MD, Jia ZF, Monteiro MJ. Complex Polymer Topologies Built from Tailored Multifunctional Cyclic Polymers. Macromolecules. 2014;47:4955-70.
